# Hybrid Genome and Clinical Impact of Emerging Extensively Drug-Resistant Priority Bacterial Pathogen *Acinetobacter baumannii* in Saudi Arabia

**DOI:** 10.3390/life15071094

**Published:** 2025-07-12

**Authors:** J. Francis Borgio

**Affiliations:** 1 Department of Genetic Research, Institute for Research and Medical Consultations (IRMC), Imam Abdulrahman Bin Faisal University, Dammam 31441, Saudi Arabia; fbalexander@iau.edu.sa; Tel.: +966-56739198; 2 Department of Epidemic Diseases Research, Institute for Research and Medical Consultations (IRMC), Imam Abdulrahman Bin Faisal University, Dammam 31441, Saudi Arabia

**Keywords:** *Acinetobacter*, Saudi Arabia, extensively drug resistant, bacterial genome, long-read sequencing, mobile genetic elements, resistome

## Abstract

*Acinetobacter baumannii* is listed by the World Health Organization as an emerging bacterial priority pathogen, the prevalence and multidrug resistance of which have been increasing. This functional genomics study aimed to understand the drug-resistance mechanisms of an extensively drug-resistant (XDR) *A. baumannii* strain (IRMCBCU95U) isolated from a transtracheal aspirate sample from a female patient with end-stage renal disease in Saudi Arabia. The whole genome of IRMCBCU95U (4.3 Mbp) was sequenced using Oxford Nanopore long-read sequencing to identify and compare the antibiotic-resistance profile and genomic features of *A. baumannii* IRMCBCU95U. The antibiogram of *A. baumannii* IRMCBCU95U revealed resistance to multiple antibiotics, including cefepime, ceftazidime, ciprofloxacin, imipenem, meropenem and piperacillin/tazobactam. A comparative genomic analysis between IRMCBCU95U and *A. baumannii* K09-14 and ATCC 19606 identified significant genetic heterogeneity and mosaicism among the strains. This analysis also demonstrated the hybrid nature of the genome of IRMCBCU95U and indicates that horizontal gene transfer may have occurred between these strains. The IRMCBCU95U genome has a diverse range of genes associated with antimicrobial resistance and mobile genetic elements (ISAba1 and IS26) associated with the spread of multidrug resistance. The presence of virulence-associated genes that are linked to iron acquisition, motility and transcriptional regulation confirmed that IRMCBCU95U is a priority human pathogen. The plasmid fragment IncFIB(pNDM-Mar) observed in the strain is homologous to the plasmid in *Klebsiella pneumoniae* (439 bp; similarity: 99.09%), which supports its antimicrobial resistance. From these observations, it can be concluded that the clinical *A. baumannii* IRMCBCU95U isolate is an emerging extensively drug-resistant human pathogen with a novel combination of resistance genes and a plasmid fragment. The complex resistome of IRMCBCU95U highlights the urgent need for genomic surveillance in hospital settings in Saudi Arabia to fight against the spread of extensively drug-resistant *A. baumannii*.

## 1. Introduction

*Acinetobacter baumannii* has emerged as a priority pathogen in healthcare settings around the world [1,2,3]. *A. baumannii* is a significant nosocomial bacterial pathogen whose prevalence and multidrug resistance (MDR) have been increasing [1,4]. This Gram-negative *coccobacillus* poses a notable threat to patients who are immunocompromised and those who require prolonged hospitalization [1,4,5]. *A. baumannii* easily colonizes the skin and is found in high concentrations within the respiratory and oropharyngeal secretions of infected patients, which contributes to the widespread prevalence of nosocomial *A. baumannii* infections [2,6]. The rise in MDR *A. baumannii* infections is a major concern due to the limited options for treatment, high mortality and poor prognoses [1,2,3]. The World Health Organization (WHO) has recognized antimicrobial resistance as one of the most important challenges for human health [2,7]. *A. baumannii* is a key member of the ESKAPE pathogens (‘*Enterococcus faecium*, *Staphylococcus aureus*, *Klebsiella pneumoniae*, *A. baumannii*, *Pseudomonas aeruginosa* and *Enterobacter* group’) due to its antibiotic resistance [2,8]. In the early 1970s, *A. baumannii* was susceptible to most antibiotics; however, it has recently been shown to exhibit extensive resistance to numerous first-line antibiotics [9]. *A. baumannii* has various resistance mechanisms including carbapenem enzyme production [10]. One of the key challenges is overcoming the ability of *A. baumannii* to acquire and utilize antimicrobial-resistance mechanisms. Resistance to antibiotics like tigecycline and polymyxins is being increasingly seen in clinical isolates. Understanding antibiotic-resistance mechanisms is critical for the use of antibiotics in clinics and for developing novel therapeutics. The ability of *A. baumannii* to rapidly develop various resistance mechanisms to antibiotics has increased the challenge of treating infected patients [4]. Beyond antibiotic resistance, *A. baumannii* has various virulence factors that contribute to its pathogenicity, including those involved in its ability to resist desiccation, form biofilms in various environments and express surface-adhesion proteins and secretion systems. These factors support the environmental persistence and host colonization of *A. baumannii* and contribute to its success as a nosocomial agent [1,2,3].

Genomic studies have revealed the genetic diversity within the clinical isolates of *A. baumannii* and their significant genetic divergence from laboratory strains [11]. These differences are clinically relevant as they can influence the expression of virulence factors and resistance genes in *A. baumannii*. The evolution of the pAB3 plasmid to pAB04, which has a higher number of resistance cassettes, is one of the best examples of adaptation in *A. baumannii*. Comparative genomics has unveiled significant genetic diversity among *A. baumannii* strains, with >2000 publicly available genomes with a genome size of nearly 12,000 genes. The accumulation of resistance mechanisms coupled with virulence factors contribute to the prevalence of *A. baumannii* as a nosocomial pathogen. In addition, the potential for *A. baumannii* to acquire toxin-related virulence factors similar to those found in other bacterial pathogens such as *Vibrio cholerae* and *Clostridium difficile* still remains a concern [3,12].

Recent studies have revealed that multidrug-resistant *A. baumannii* is prevalent across the Arabian Peninsula [13]. Studies from Saudi Arabia reported high mortality rates associated with MDR microbes, with *A. baumannii* being among the top three pathogenic microorganisms associated with high mortality [13]. In Saudi Arabia, *A. baumannii* has been isolated from various human samples including urine, blood and respiratory specimens [14,15,16]. There is an urgent need for alternative treatments against MDR *A. baumannii* such as phage therapy, metabolic interference, antimicrobial peptides and vaccines [3,17,18]. Recent clinical isolates of *A. baumannii* exhibit significant genetic differences from the type strains such as ATCC 17978 and ATCC 19606. These strains have served as primary research models for decades. However, these older strains were isolated nearly 70 years ago and may not accurately reflect the characteristics of clinical isolates recovered in the past three decades. Therefore, the utilization of modern techniques to assess clinical isolates is essential for obtaining more accurate and updated details of the molecular mechanisms underlying the survival and adaptation of *A. baumannii* in recent years. The use of advanced molecular and bioinformatics techniques may lead to the identification of novel and relevant virulence factors beyond the current knowledge on resistance mechanisms [3,19]. In the current study, the phenome and resistome of the clinical isolate *A. baumannii* IRMCBCU95U were characterized. This isolate was isolated from a transtracheal aspirate from a Saudi Arabian female patient with end-stage renal disease who was admitted to a tertiary care hospital in the Eastern Province of Saudi Arabia due to an acute condition requiring intubation.

## 2. Materials and Methods

### 2.1. Ethical Approval

The current study received ethical approval from the Institutional Review Board (IRB) of Imam Abdulrahman Bin Faisal University (Ref No.: IRB-2022-13-462). All experimental procedures were conducted in accordance with the principles outlined in the 1964 Declaration of Helsinki.

### 2.2. Bacterial Strain and DNA Isolation

A pathogenic bacterial strain was isolated from a transtracheal aspirates collected by the treating medical team at King Fahd Hospital of the University, Imam Abdulrahman Bin Faisal University, as part of a standard clinical investigation. The transtracheal aspirate was obtained from a 31-year-old female patient of Arab origin. The patient presented with end-stage renal disease requiring hemodialysis and was hospitalized with an acute condition necessitating intubation at the time of sampling. The pathogenic strain (IRMCBCU95U) was isolated as elaborated in [20]. Antimicrobial susceptibility profiling of the clinical isolate IRMCBCU95U was performed using the VITEK^®^ 2 automated system. This standardized assay enabled a quantitative assessment of the isolate’s resistance phenotypes for a panel of clinically relevant antibiotics and allowed for elucidation of the potential resistance mechanisms based on established criteria from the manufacturers. Genomic DNA was extracted from the isolate using the Puregene Yeast/Bact. Kit B (Qiagen, Hilden, Germany), following the manufacturer’s recommended protocol. This kit employs a salting-out procedure to selectively precipitate proteins and yield high-molecular-weight genomic DNA that is suitable for downstream sequencing applications. The purity and concentration of the extracted genomic DNA of IRMCBCU95U were evaluated using a Nanodrop 2000 spectrophotometer (Thermo Scientific, Waltham, MA, USA). The 260/280 nm and 260/230 nm absorbance ratios were assessed to determine the concentrations of protein and other contaminants. The DNA was quantified by measuring the absorbance at 260 nm, and was then used for the subsequent genomic analyses.

### 2.3. Nanopore Whole-Genome Sequencing and Data Processing

Whole-genome sequencing of the *Acinetobacter* clinical isolate IRMCBCU95U was performed using Oxford Nanopore long-read technology at the IRMC Genetics Laboratory. High-quality genomic DNA meeting the purity criteria of a 260/280 nm ratio between 1.8 and 2.0, a 260/230 nm ratio between 2.0 and 2.2, and a concentration of 100–500 ng in 50 µL was subjected to DNA repair and end preparation using NEBNext reagents. Following AMPure XP bead-based cleanup, the end-prepped DNA (>700 ng) was barcoded using the native barcoding kit and ligated. The barcoded samples were pooled to create a sample with a total of 700 ng of DNA and adapter ligation was performed using the NEBNext Quick Ligation Reaction Buffer and Adapter Mix II (Oxford Nanopore Technologies, Oxford, UK). The resulting library that was recovered contained approximately 430 ng of DNA and was cleaned using AMPure XP beads and optimized using long fragment buffer (LFB). The prepared library was loaded onto a primed Oxford Nanopore SpotON flow cell (Oxford Nanopore Technologies, Oxford, UK). Sequencing was conducted using the MinION Mk1C device (Oxford Nanopore Technologies, Oxford, UK) with the real-time data acquisition and base calling performed using the MinKNOW software (version 23.04.6) (Oxford Nanopore Technologies, Oxford, UK). The initial quality and coverage metrics were determined using the EPI2ME platform (EPI2ME Agent 3.5.7). Raw signal data were base called and converted into fastQ format for the downstream genomic analyses.

### 2.4. Bacterial Genome Analysis

The raw sequencing reads from the IRMCBCU95U sample were processed using the NanoForms pipeline. This pipeline included quality-assessment modules to evaluate various metrics of the sequencing run [21]. The long-read sequencing data for the *Acinetobacter* clinical isolate IRMCBCU95U were processed using several bioinformatics tools. Fastp [22], Nanoforms [21] and NanoFilt [23] were used for quality control and filtering and trimming of the reads, while Quast [24], Flye [25], the PATRIC (BV-BRC 3.28.5) software [26] and NanoGalaxy [27] were employed for de novo assembly, assembly quality assessment and genome annotation [28,29]. The taxonomic classification of the *Acinetobacter* isolate IRMCBCU95U was determined using a whole-genome phylogenetic tree. The average G+C content and the number of contigs were calculated. The predicted proteins were functionally annotated according to enzyme commission (EC) numbers [30], metabolic pathways [31], gene ontology (GO) [32], protein family classifications [33] and protein complex subsystems [34]. The genome of *Acinetobacter* IRMCBCU95U was also analyzed to identify genes associated with antibiotic-resistant genes [35], drug targets [36,37], virulence factors [38,39] and transport systems [40]. Established plasmid types were identified through plasmid typing [41]. K-mer-based methods [26] were employed to identify antimicrobial-resistance (AMR) genes. The phylogenetic analysis utilized 100 genes retrieved from the NCBI reference database supplemented with representative genomes selected via the Mash/MinHash algorithm (Mash v2.3) [42]. Genome alignment was performed using MUSCLE [43], with the statistical support assessed using bootstrapping [44,45]. The metagenomic reads were mapped against reference sequences in the VFDB (2019) and CARD (2020) databases [46].

The sequence of the IRMCBCU95U genome was queried against the VFDB core dataset using BLASTN (2.16.0). The BLASTN search was performed using the online interface provided by the VFDB database with the default parameters to identify homologous sequences to those of known virulence factors. Significant hits were defined as those with an E-value of 0.0. The results were analyzed and the identity percentage, score (bits), length, number of identities, gaps and strand information were extracted and compiled [47]. The genome sequence of IRMCBCU95U was analyzed using the PlasmidFinder 2.0 server to identify plasmid sequences. PlasmidFinder 2.0 is a tool designed for the in silico detection and typing of plasmids in bacterial genome sequences. The analysis was performed using the default parameters of the PlasmidFinder 2.0 server [41]. ResFinderFG (Version 2.0) was employed to predict the resistance phenotypes of IRMCBCU95U. Linezolid resistance determinants in this isolate were further investigated by identifying mutations in relevant genes using the LRE-Finder software (Version 1.0) [46,48]. Mobile genetic elements and pathogenic protein families related to antibiotic resistance were detected using PathogenFinder (Version 1.1) [49] and MGE [50]. The genome of the IRMCBCU95U strain was also subjected to an analysis for the presence of mycotoxin biosynthesis genes using the ToxFinder-1.0 tool. The input file was in FASTA format. ToxFinder-1.0 was used to screen for genes associated with the production of ochratoxin, fumonisin, trichothecene, citrinin, aflatoxin, ergot and patulin [46,51].

## 3. Results

This functional genomics study isolated a pathogenic bacterial strain that was named IRMCBCU95U. The isolate was obtained from a transtracheal aspirate specimen from a 31-year-old female patient of Saudi Arabian origin. The clinical history of the patient included end-stage renal disease managed with hemodialysis and, at the time of sampling, the patient had been admitted to a tertiary care hospital in the Eastern Province of Saudi Arabia with an acute condition requiring intubation.

### 3.1. Phenomics of A. baumannii Strain IRMCBCU95U

The antibiogram of the extensively drug-resistant *A. baumannii* strain IRMCBCU95U was characterized through antibiotic susceptibility testing (AST) and resistance mechanism detection utilizing the VITEK^®^ 2 system. Table 1 provides the minimum inhibitory concentrations (MICs) and their corresponding interpretations (Resistant or Sensitive) for each antibiotic. The antibiotic susceptibility profile of *A. baumannii* strain IRMCBCU95U revealed that the isolate is resistant to several of the tested antibiotics including cefepime, ceftazidime, ciprofloxacin, imipenem, meropenem and piperacillin/tazobactam. The isolate is also sensitive to tigecycline (MIC = 2) and trimethoprim/sulfamethoxazole. The data indicate that ciprofloxacin-resistant isolates are likely to also be levofloxacin resistant.

### 3.2. Genomics of IRMCBCU95U

#### Quality of the Sequencing Data for IRMCBCU95U Genome

**Figure 1:**
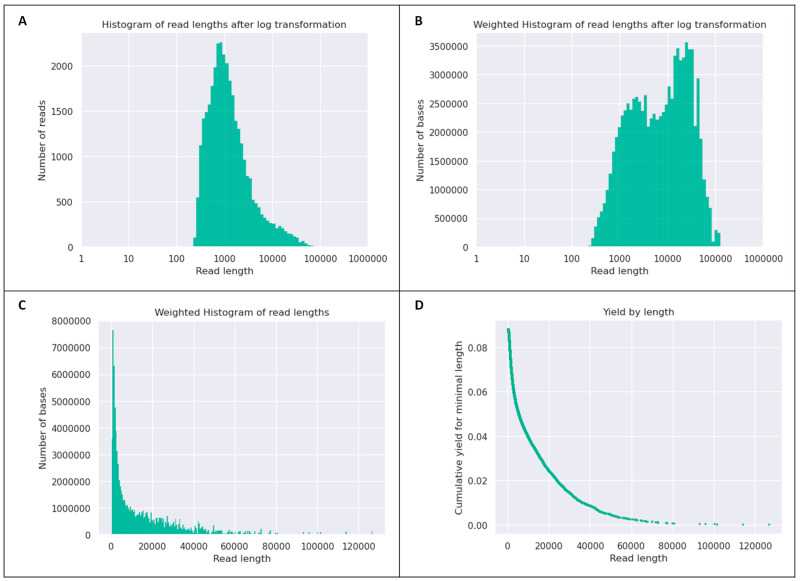
Characterization of Nanopore read lengths from the *A. baumannii* IRMCBCU95U sequencing run. (**A**) Frequency distribution of different read lengths from the IRMCBCU95U sequencing run (logarithmic scale), highlighting the range and most common lengths of the raw reads. (**B**) Total number of bases contributed by reads with a specific length, providing insight into the overall IRMCBCU95U sequencing yield for different read sizes. (**C**) Total base yield per read length of the IRMCBCU95U sequencing run, with the *x*-axis transformed logarithmically to better visualize the distribution across a wide range of read lengths. (**D**) Proportion of the total sequencing yield (bases) as a function of increasing minimum read length of the IRMCBCU95U sequencing run, indicating how many data were retained when filtering for longer reads.

The NanoForms analysis indicated the successful processing of the sequencing genome data using long-read Nanopore sequencing, with the workflow completing without any errors. A total of 33,004 reads were generated for the IRMCBCU95U genomic DNA. The mean and median read lengths were 2665.9 and 1040.0, respectively, with an N50 read length of 7677, indicating a substantial proportion of longer reads. The total yield of the genome sequencing run was 87.98 million bases. The read quality assessment revealed that all the reads exceeded a quality threshold and 100% of the reads had a quality score above Q5. While 65.2% of the reads had a quality score above Q10, only 24.1% had a score exceeding Q12 and 0.3% had a quality score above Q15. This suggests a moderately high read quality with a reduction in the number of high-confidence base calls with more stringent quality filters (Supplementary Table S1). Figure 1 shows a detailed characterization of the read length distribution in the sequencing data and presents various metrics illustrating the quality and distribution of sequencing reads obtained for the IRMCBCU95U isolate.

### 3.3. Assembly of the IRMCBCU9SU Dataset

The assembly of the IRMCBCU9SU genome sequence using NanoForms resulted in a successful workflow (Supplementary Table S2). A total of 40 contigs were identified during the consensus assembly of the fastq files of the IRMCBCU9SU genome sequence. The N50 improved from the initial assembly (167,919 bp) to the polished genome (807,720 bp), but decreased in the consensus genome (405,948). This indicates that polishing initially increased the contig length, but the consensus step might have fragmented some contigs to improve accuracy. The largest contig was substantial, exceeding 1.4 Mbp in the initial assembly and polished genome. The total assembly length was approximately 4.3 Mbp across all versions. The GC content was around 40%. There were zero mismatches, suggesting a high degree of agreement within the assembly process.

### 3.4. Phylogenetic Tree Based on Whole Genomes of IRMCBCU95U and Other Acinetobacter Species

**Figure 2:**
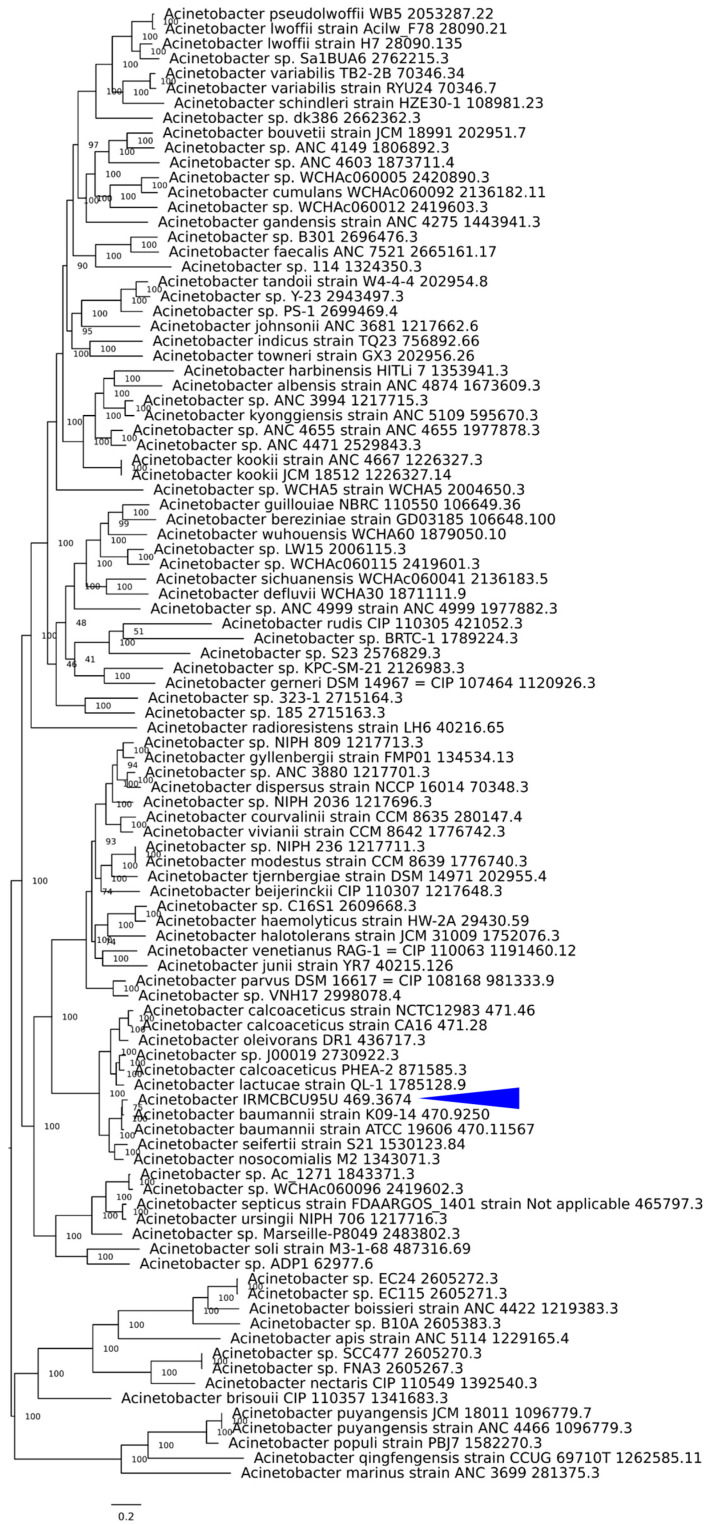
Phylogenetic tree based on whole genomes of *Acinetobacter* IRMCBCU95U (pointed with blue square) and 98 additional *Acinetobacter* strains (see Supplementary Table S3 for a comprehensive list of the genomes and corresponding strain designations). The alignment of 100 core single-copy orthologous genes comprising 31,071 amino acid residues (equivalent to 93,213 nucleotide bases) was generated using the MAFFT multiple sequence alignment program (Supplementary Table S4). The resulting alignment served as the basis for the phylogenetic tree construction employing the randomized accelerated maximum likelihood algorithm with rapid bootstrap analysis to assess branch support.

The phylogenetic tree was constructed using 99 whole reference genomes (Supplementary Table S3). The tree was used to elucidate the evolutionary relationships between *Acinetobacter* IRMCBCU95U and other *Acinetobacter* species. The tree was built using alignments of 100 single-copy orthologous genes, which are found in all 98 reference genomes and the genome of the clinical isolate (Supplementary Table S4). The amino acid sequences were aligned using MAFFT and the final tree was constructed using randomized accelerated maximum likelihood. The best-fit model for protein evolution was determined to be the LG substitution model. Branch support was assessed using randomized accelerated maximum likelihood fast bootstrapping. It was confirmed that the constructed phylogenetic tree includes various *Acinetobacter* species with *Acinetobacter* IRMCBCU95U positioned among them. The tree illustrates the close relationships between IRMCBCU95U and species such as *A. baumannii* strains K09-14 and ATCC 19606, and its divergence and relatedness to other *Acinetobacter* species (Figure 2).

A bootstrap value of 75 on a branch connected to *Acinetobacter* IRMCBCU95U was observed in the phylogenetic tree, which indicates moderate support for the evolutionary relationship. This suggests that, in 75% of the bootstrap replicates, *Acinetobacter* IRMCBCU95U and the *A. baumannii* strains K09-14 and ATCC 19606 clustered together. This provides reasonable evidence that the evolutionary relationship shown is likely correct.

### 3.5. Genomic Overview of A. baumannii IRMCBCU95U

**Figure 3:**
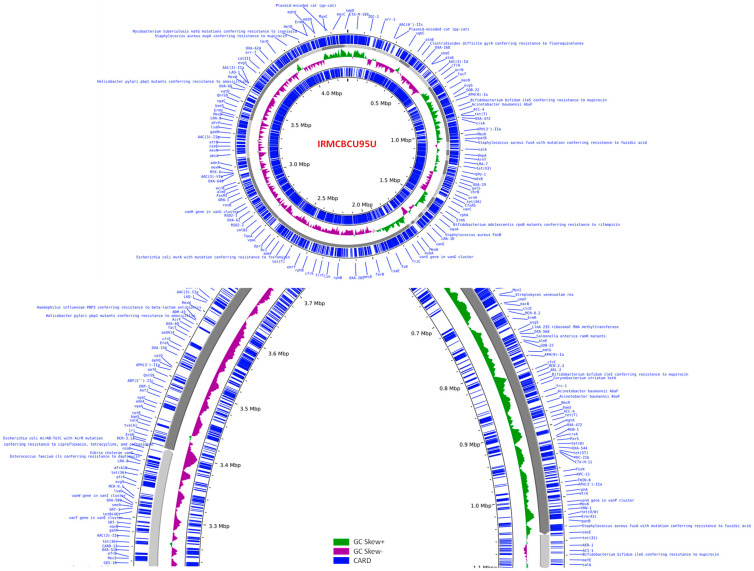
Graphical representation of the annotated *A. baumannii* IRMCBCU95U genome. The outermost ring illustrates the drug-resistance genes identified through the Comprehensive Antibiotic Resistance Database (CARD). Top panel: circular map of the *A. baumannii* IRMCBCU95U genome. Bottom panel: selected regions of the *Acinetobacter* IRMCBCU95U genome projected to indicate the location of mutated genes.

The genome of *A. baumannii* IRMCBCU95U was comprehensively annotated, revealing a genome length of 4,340,742 base pairs (bp) distributed across 40 contigs. The average GC content of the genome was 40.46%. The assembly statistics indicated a contig N50 of 404,531 bp and a contig L50 of 4. Annotation of the genome identified a total of 7437 protein-coding sequences (CDSs). Additionally, the genome contains 89 transfer RNA (tRNA) genes and 17 ribosomal RNA (rRNA) genes. The annotation analysis also predicted 1737 hypothetical proteins and 5700 proteins with functional assignments. The functional annotation included the assignment of 1896 enzyme commission numbers, 1634 gene ontology terms and KEGG pathways for 1464 proteins. The initial analysis of the specialty genes within the genome revealed the presence of antibiotic-resistance genes. In addition, 50 and 10 genes were identified as potential drug targets (by DrugBank and TTD, respectively), 139 and 9 were identified as transporter genes (by TCDB and PATRIC_VF, respectively), and 14 (VFDB) and 40 (Victors) were identified as virulence factors. The annotated genome of *A. baumannii* IRMCBCU95U is displayed in Figure 3. The outer ring of the genome map displays the drug-resistance genes that were identified using a comprehensive antibiotic-resistance database (Figure 3, Figures S1 and S2). Based on the cgMLST analysis of the IRMCBCU95U genome and the *A. baumannii* reference genome, the sequence type (cgST) was determined to be ST436. Out of the 2133 loci examined, 631 alleles were called, which represent 29.58% of all the loci. When compared to the cgST, 624 alleles matched, indicating a 29.25% allele match with the cgST.

### 3.6. Genome Comparison

**Figure 4:**
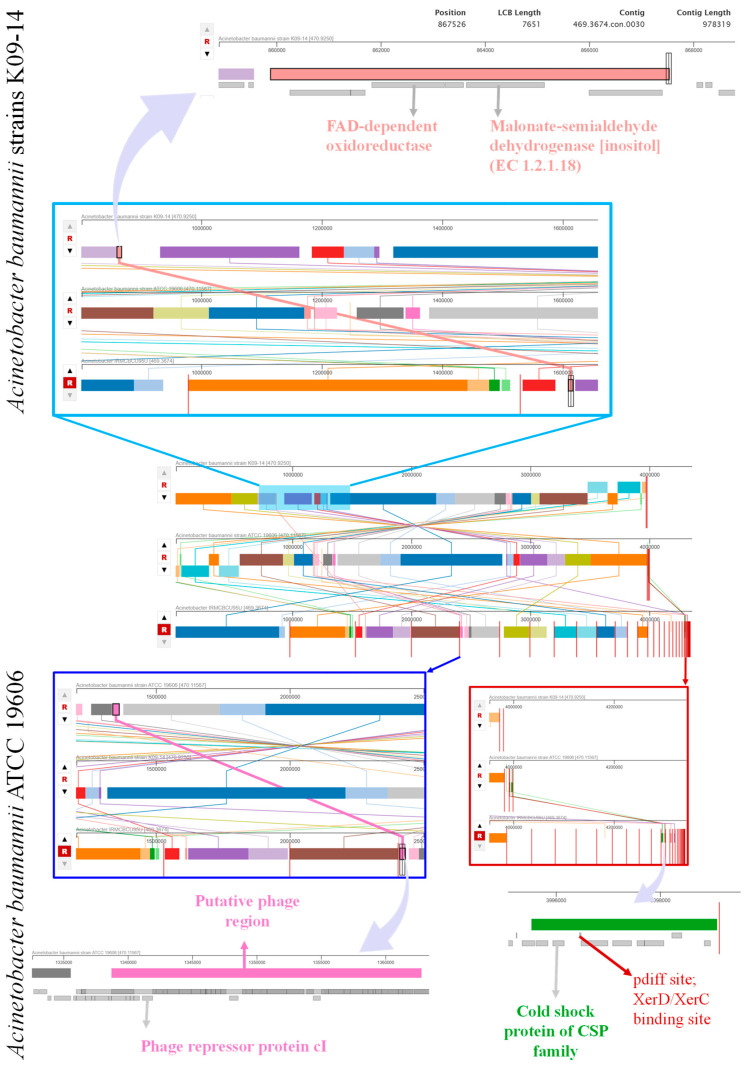
Genome alignment of *A. baumannii* IRMCBCU95U, K09-14 and ATCC 19606.

The genome alignment was performed using three *A. baumannii* strains: IRMCBCU95U, K09-14 and ATCC 19606. The analysis revealed the presence of specific genes from both the *A. baumannii* K09-14 and ATCC 19606 genomes within the IRMCBCU95U isolate genome (Figure 4). Specifically, the genes encoding FAD-dependent oxidoreductase and malonate-semialdehyde dehydrogenase (inositol) (EC 1.2.1.18) that are present in the *A. baumannii* K09-14 genome were also observed in the *Acinetobacter* IRMCBCU95U genome. However, these genes were absent in the *A. baumannii* ATCC 19606 genome. Genes in the *A. baumannii* ATCC 19606 genome, including those encoding a putative phage region, the phage repressor protein cI, a cold shock protein of the CSP family and the *pdiff* site/*XerD/XerC*-binding site, were found in the *A. baumannii* IRMCBCU95U genome but not in the *A. baumannii* K09-14 genome. The alignment revealed a mosaic pattern of the presence and absence of different genes across these strains, suggesting genetic diversity and potential horizontal gene transfer events.

### 3.7. Genome-Assisted Resistant Phenotype

Resistance Gene Identification

The resistome analysis of *A. baumannii* IRMCBCU95U revealed a genetic basis for its multidrug-resistant phenotype (Table 2). This clinical isolate harbors several acquired antimicrobial-resistance genes that confer resistance to multiple antibiotic classes. The *armA* gene was detected, which mediates resistance to aminoglycosides such as gentamicin, tobramycin, amikacin, isepamicin and netilmicin. The *aph*(*3′*)-*Ia* gene confers resistance to other aminoglycosides, including kanamycin, neomycin, lividomycin, paromomycin and ribostamycin. Beta-lactam resistance is attributed to the presence of bla_TEM-1D_, bla_OXA-23_, bla_OXA-66_, bla_ADC-25_ and bla_OXA-422_ genes. Bla_TEM-1D_ is associated with resistance to amoxicillin, ampicillin, piperacillin, ticarcillin and cephalothin. The presence of bla_OXA-23_ is particularly concerning as it confers resistance to carbapenems (imipenem and meropenem). The strain also carries *msr*(*E*) and *mph*(*E*) genes that contribute to macrolide resistance—specifically, to erythromycin and azithromycin—as well as resistance to streptogramin B antibiotics (quinupristin, pristinamycin Ia and virginiamycin S). The identification of these resistance genes aligns with the observed resistance phenotypes, confirming the genomic basis of the extensively drug-resistant profile of *A. baumannii* IRMCBCU95U (Table 2 and Table S5).

The IRMCBCU95U genome was also analyzed using KmerResistance-2.2 to identify antimicrobial-resistance genes (Table 3). The analysis revealed the presence of several genes associated with resistance to multiple classes of antibiotics. Genes conferring resistance to beta-lactams (bla_OXA-23_, bla_OXA-66_, bla_TEM-1D_ and bla_ADC-25_), aminoglycosides (*aph*(*3′*)-*la* and *armA*) and macrolides (*msr*(*E*) and *mph*(*E*)) were also detected using this method. The genome also showed significant similarity to the *A. baumannii* strain AB217-VUB. The findings using KmerResistance suggest that IRMCBCU95U is a multidrug-resistant strain and could pose a clinical challenge.

### 3.8. Virulence Factors

The BLASTN analysis using a virulence factor database revealed the presence of several genes in the IRMCBCU95U genome with high homology to known virulence factors. The top ten hits are listed in Table 4. A variety of virulence-associated genes were identified, including those involved in iron acquisition, motility and transcriptional regulation. Genes related to iron acquisition were prominently represented with several hits matching TonB-dependent receptors (VFG037497), porin family proteins (VFG037513), FecR domain-containing proteins (VFG037490) and transferrin-binding protein-like proteins (VFG037505). The identified factors are vital for the survival and proliferation of *A. baumannii* within a host. Iron is an essential nutrient and its availability is limited in hosts. Genes associated with Type IV pilus biogenesis and twitching motility (*pilU* (VFG050400) and *pilT* (VFG050386)) were also identified in the IRMCBCU95U genome. Type IV pili have significant roles in the adherence of bacteria to host cells and biofilm formation. The presence of genes encoding transcriptional regulators such as the LysR family transcriptional regulator (VFG037470) and the sigma-70 family RNA polymerase sigma factor (VFG037482) suggest that *A. baumannii* IRMCBCU95U possesses complex mechanisms that regulate the expression of virulence genes based on environmental signals. A gene involved in heme metabolism and biliverdin-producing heme oxygenase (*hemO*, VFG037529) was also identified in the *A. baumannii* IRMCBCU95U genome.

### 3.9. Mobile Elements in A. baumannii IRMCBCU95U

The genome of *A. baumannii* IRMCBCU95U was analyzed for the presence of mobile genetic elements (MGEs) using the MobileElementFinder tool. The analysis revealed a variety of insertion sequences (ISs) and composite transposons, highlighting the dynamic nature of its genome (Supplementary Table S6). Several distinct IS families were identified with the most prevalent being the IS4 family. Multiple copies of ISAba1 were found across different contigs. ISAba1 is known to be associated with the mobilization of antibiotic-resistance genes in *A. baumannii*. The results showed several entries for ISAba1 with varying positions in the contigs and a high sequence identity (generally > 99%) and coverage. Multiple copies of IS26 were also detected. IS26 is frequently associated with the formation of composite transposons and the spread of resistance genes. ISAba24, an insertion sequence belonging to the IS66 family, was also detected. The analysis also identified several putative composite transposons, which are formed when two IS elements flank a region of DNA. These are of particular concern due to their ability to mobilize larger DNA fragments, often including antibiotic-resistance genes. Composite transposons such as cn_3222_IS26, cn_5210_IS26, cn_2898_IS26, cn_45174_ISAba1, cn_11212_ISAba1, cn_20460_ISAba1 and cn_26891_ISAba1 were identified in the genome of *A. baumannii* strain IRMCBCU95U. The composite transposons cn_3222_IS26, cn_5210_IS26 and cn_2898_IS26 contain IS26 elements, while the composite transposons cn_45174_ISAba1, cn_11212_ISAba1, cn_20460_ISAba1 and cn_26891_ISAba1 contain ISAba1 elements.

### 3.10. Pathogenic Proteins

*A. baumannii* strain IRMCBCU95U was also analyzed using PathogenFinder 1 and PathogenFinder 2 to predict its pathogenic potential. The analysis from PathogenFinder 1 predicted *Acinetobacter* IRMCBCU95U to be a human pathogen (probability of 0.918). The input proteome coverage was 6.64% with 494 matched pathogenic families and zero matches with non-pathogenic families (Supplementary Table S7). Supplementary Table S7 details the identified genes associated with antimicrobial resistance and virulence determinants within the *A. baumannii* IRMCBCU95U genome, including the protein functions and genomic locations. While the table primarily focuses on gene identification and function, the protein ID and protein product of these genes is also provided to help understand the pathogenic potential of IRMCBCU95U. A total of 7437 sequences were analyzed with an average sequence length of 155.0 bp. The analysis identified several proteins with 100% identity to known proteins in other *Acinetobacter* strains including the exodeoxyribonuclease V beta chain, the two-component system sensor histidine kinase, an uncharacterized protein conserved in bacteria, an outer membrane component of the TAM transport system, an outer membrane autotransporter barrel domain, DNA internalization-related competence protein ComEC/Rec2, outer membrane receptor proteins, a predicted exporter, general secretion pathway protein D, an outer membrane receptor for monomeric catechols, an outer membrane receptor for ferric coprogen and ferric-rhodotorulic acid, a diguanylate cyclase/phosphodiesterase, a probable site-specific recombinase, a putative membrane protein, a Zn-dependent oligopeptidase, outer membrane vitamin B12 receptor BtuB, DNA primase DnaG, hypothetical proteins, a dipeptide ABC transporter, substrate-binding protein DppA, an uncharacterized siderophore S biosynthesis protein, an AcsD-like aminopeptidase N family protein, a poly (glycerol-phosphate) alpha-glucosyltransferase, a putative permease, a SAM-dependent methyltransferase, cytoskeleton protein RodZ, a putative threonine efflux protein, a lytic transglycosylase, transcriptional regulators, 5-formyltetrahydrofolate cyclo-ligase, outer membrane protein W precursor, UPF0301 protein YqgE, a conserved hypothetical protein, NADH ubiquinone oxidoreductase chain A, a predicted 3-hydroxylacyl-(acyl carrier protein) dehydratase, a signal peptide, protein GlcG, DNA-binding protein Fis, phospholipid ABC transporter-binding protein MlaB and Z-ring-associated protein ZapA. PathogenFinder 2 also predicted *Acinetobacter* IRMCBCU95U to be a human pathogen. The mean prediction score was 0.8737 ± 0.1079 based on the predictions from four neural networks: neural network 1 (0.8276), neural network 2 (0.7188), neural network 3 (0.9619) and neural network 4 (0.9863).

### 3.11. Mycotoxin-Producing Genes

The genome of IRMCBCU95U was analyzed using the ToxFinder-1.0 tool to identify potential mycotoxin-producing genes. The analysis revealed no significant hits for the genes involved in the biosynthesis of ochratoxin, fumonisin, trichothecene, citrinin, aflatoxin, ergot and patulin. This suggests that IRMCBCU95U does not possess the genetic potential to produce these mycotoxins.

### 3.12. Plasmid Analysis in IRMCBCU95U

The PlasmidFinder 2.0 analysis of the *A. baumannii* strain IRMCBCU95U genome revealed the presence of a plasmid fragment identified as IncFIB(pNDM-Mar) (Supplementary Table S8). This plasmid fragment showed a high identity (100%) over a query/template length of 439 bp. The 439 bp fragment in the IRMCBCU95U genome is identical to *Klebsiella pneumoniae* plasmid pNDM-MAR (GenBank accession number JN420336.1). This fragment was located on the plus strand at position 29,199 to 29,637. The 439 bp fragment in the IRMCBCU95U genome partially encodes a RepB family plasmid replication initiator protein, a protein that can initiate the replication of certain plasmids.

## 4. Discussion

The prevalence of multidrug-resistant *A. baumannii* is a significant concern across the nine countries of the Arabian Peninsula where it is frequently the most prevalent MDR bacteria, demonstrating its ability to persist in regions with hot and dry summers [13]. The initial identification of *A. baumannii* and other reports from various regions of the Kingdom of Saudi Arabia reported the presence of this pathogen in various human samples such as urine, blood cultures, respiratory specimens, skin and soft tissue specimens, sterile body fluids, wound swabs, rectal swabs and sputum [14,15,16]. Studies from Saudi Arabia have reported high mortality rates associated with MDR microbes, with *A. baumannii*, *Mycobacterium tuberculosis* and *Pseudomonas aeruginosa* being the top three organisms [13]. Analyses of *A. baumannii* strains isolated from Saudi Arabia have revealed the presence of diverse antimicrobial-resistance genes [52,53]. Core genome multilocus sequence typing (cgMLST) of the IRMCBCU95U isolate yielded a cgMLST436 profile, which is distinct from that of the previously characterized *A. baumannii* isolate AB-JZ67 (cgMLST785) from the same country [53] and other strains characterized that were as cgMLST785, cgMLST909, cgMLST1295, cgMLST906, cgMLST779, cgMLST556, cgMLST406 [54], cgMLST604, cgMLST45 and cgMLST2 [55].

The presence of multiple IS elements (ISAba1 and IS26) and composite transposons in the IRMCBCU95U genome is significant as the abundance of MGEs clearly indicates a high degree of genome plasticity in the IRMCBCU95U genome. This enables *A. baumannii* to rapidly adapt to changing environments. These MGEs and composite transposons are key factors in the accumulation of antibiotic-resistance genes in the IRMCBCU95U genome. There may be a high risk of horizontal transfer of resistance genes and antibiotic resistance spread due to the presence of MGEs in IRMCBCU95U. MGEs carry genes involved in virulence and could potentially contribute to the pathogenicity of IRMCBCU95U. An earlier study from a healthcare facility in the Western Region of Saudi Arabia that reported the detection of *A. baumannii* isolates, including the Ab27-HEnv isolate, and the present study on *A. baumannii* IRMCBCU95U highlight the significant presence of mobile genetic elements associated with antimicrobial resistance [56]. The previous study from Saudi Arabia identified seven MGEs in the Ab27-HEnv isolate, which are composed of five insertion sequences and two composite transposons with resistance genes for aminoglycosides (*aph(6)-Id*, *aph(3″)-Ib*, *aph(3′)*-Via and *armA*) and macrolides (*mphE*) and are present on the contigs with MGEs. *IS26*, *ISVsa3*, *ISAba24* and *ISAba26* were also identified in the clinical isolates of a Saudi strain from the previous study [56]. Similarly, the isolate studied in the present study (IRMCBCU95U) revealed a variety of insertion sequences and composite transposons in its genome, confirming the resistance *A. baumannii* strains. Insertion sequences *IS26* and *ISAba24* that were found in a previously reported isolate (Ab27-HEnv) were also detected in IRMCBCU95U. *ISAba1* is associated with the mobilization of antibiotic-resistance genes in *A. baumannii*; there were multiple copies of this sequence in different contigs from the IRMCBCU95U genome. The prevalence of the IS4 family and the identification of multiple copies of *ISAba1* and *IS26* in the present study suggest that these elements may play a particularly important role in genome plasticity and resistance gene dissemination for the IRMCBCU95U strain. Consistent with the observations in a previous study from a healthcare facility in the Western Region of Saudi Arabia, the present study conducted in the Eastern Region of Saudi Arabia also detected the aminoglycoside-resistance genes *aph*(*3′*)-*Ia* and *armA* in an *A. baumannii* strain. This concordance underscores the importance of nationwide genomic surveillance in Saudi Arabia. A significant finding in the two studies was the identification of composite transposons that are capable of mobilizing larger DNA fragments containing antibiotic-resistance genes. The presence of both *IS26-* and *ISAba1*-containing composite transposons strengthens the importance of IS elements in the spread of resistance genes in Saudi Arabia [54,56]. The identification of similar MGEs and the prevalence of *ISAba1* suggest common mechanisms of resistance genes and their spread in this species.

Isolates from a neighboring country (Yemen) contain naturally occurring bla_OXA-51_ and carbapenemase-encoding bla_OXA-23_ genes. An *A. baumannii* isolate was reported to have the acetyltransferase *aac(6′-Ib)* gene associated with aminoglycoside resistance. All isolates from the study had the fluoroquinolone-resistance-associated *GyrA* gene with a Ser83Leu substitution [57]. These regional findings align with the comparative genomic analysis of IRMCBCU95U that revealed significant genetic heterogeneity and mosaicism among *A. baumannii* strains. The hybrid genome observed in the studied IRMCBCU95U isolate contains genes from both K09-14 and ATCC 19606, indicating the importance of horizontal gene transfer during the evolution of *A. baumannii*. The detection of diverse antimicrobial-resistance genes in IRMCBCU95U is consistent with the extensive resistance profiles reported in clinical isolates from Yemen, which may be contributing to the challenges in treating *A. baumannii* infections in the region [13,57].

The genome alignment revealed a mosaic pattern of the presence and absence of difference genes across the K09-14, ATCC 19606 and IRMCBCU95U strains, suggesting genetic diversity and potential horizontal gene transfer events. The hybrid genome of the IRMCBCU95U strain contains genes specific to both K09-14 and ATCC 19606, indicating that IRMCBCU95U has acquired genetic material from both strains. IRMCBCU95U shares genes encoding FAD-dependent oxidoreductase and malonate-semialdehyde dehydrogenase (inositol) with K09-14 but these are absent in ATCC 19606, suggesting potential differences in metabolic capabilities between the strains. IRMCBCU95U also shares genes related to a putative phage repressor protein cI, cold shock protein (CSP family) and the *pdiff* site/*XerD/XerC*-binding site with ATCC 19606 which are absent in K09-14.

The identification of a broad range of virulence-associated genes in IRMCBCU95U using the VFDB database provides valuable insights into the mechanisms underlying its pathogenicity [47]. The presence of multiple iron’ systems supports its survival and pathogenesis. Similarly, the genes related to pilus formation contribute to host cell interactions and biofilm development. The identification of transcriptional regulators emphasizes the adaptability of *A. baumannii* to different host environments. It is important to note that this in silico analysis only provides a prediction of potential virulence factors. Further experimental studies are necessary to confirm the expression and functional roles of these genes in the pathogenesis of *A. baumannii* IRMCBCU95U. The genome data of IRMCBCU95U lay a solid foundation for future investigations into the molecular mechanisms of virulence in this clinically relevant isolate.

The KmerResistance analysis of the IRMCBCU95U genome provides evidence for its potential multidrug-resistant nature, which was reported in earlier studies [46,58]. The identification of several key antimicrobial-resistance genes highlights the ability of IRMCBCU95U to withstand the effects of multiple antibiotics and has significant implications for its clinical management and potential spread. The detection of beta-lactamase genes including *bla_OXA-23_, bla_OXA-66_, bla_TEM-1D_* and *bla_ADC-25_* are particularly concerning for Saudi Arabia [54,56]. The *bla_OXA_* genes frequently found in *A. baumannii* are associated with resistance to carbapenems. The identification of the aminoglycoside-resistance genes *aph(3′)-la* and *armA* indicate that IRMCBCU95U is resistant to aminoglycoside antibiotics, which was observed in a previous study in Saudi Arabia [56]. The presence of the macrolide-resistance genes *msr(E)* and *mph(E)* adds another layer of complexity to IRMCBCU95U. The high similarity of the *A. baumannii* IRMCBCU95U and AB217-VUB genomes highlights the importance of further research. *A. baumannii* is believed to be an opportunistic pathogen and is known for its ability to acquire and spread resistance genes.

Cold shock proteins are involved in stress responses and *pdiff*/*XerD/XerC* sites are involved in recombination. The sharing of genes between IRMCBCU95U and the other two strains (K09-14 and ATCC 19606) indicate that they have the ability to rapidly acquire new traits, potentially contributing to their antibiotic resistance and adaptation to different environments. The identification of the IncFIB(pNDM-Mar) plasmid fragment in *A. baumannii* IRMCBCU95U is important. IncFIB plasmids are commonly found in Enterobacteriaceae and are known to be involved in the dissemination of antimicrobial-resistance genes, including those encoding carbapenemases such as NDM (New Delhi Metallo-beta-lactamase) [59,60,61]. The presence of this plasmid fragment suggests that *A. baumannii* IRMCBCU95U might have the potential to acquire the resistance genes associated with this type of plasmid. It is important to note that the identified fragment of the IncFIB(pNDM-Mar) plasmid may have been partially integrated into the chromosome and suggests interspecies horizontal gene transfer from *K. pneumoniae* to *A. baumannii* in clinical settings. This gene transfer highlights an epidemiological concern regarding resistance genes, as they can readily cross species barriers, accelerating the spread of MDR and creating a hospital resistance reservoir. Understanding these transfers is crucial for targeted interventions against pan-drug-resistant bacteria. Further investigation such as Sanger sequencing-based assays are needed to determine the complete structure and context of this plasmid. Finding an IncFIB-related sequence from Enterobacteriaceae in *A. baumannii* highlights the importance of horizontal gene transfer in the evolution of antimicrobial resistance across different bacterial species.

## 5. Conclusions

The comparative genomic analysis of *A. baumannii* strains K09-14, ATCC 19606 and IRMCBCU95U revealed significant genetic heterogeneity and mosaicism. The studied isolate IRMCBCU95U was found to have a hybrid genome with genes from K09-14 and ATCC 19606, indicating a history of horizontal gene transfers which could potentially influence clinically relevant traits. The resistance gene analysis found that IRMCBCU95U possesses antimicrobial-resistance genes that confer resistance to multiple antibiotic classes. These findings indicate the importance of comprehensive surveillance and characterization of resistance factors in the bacterial pathogens that are prevalent in Saudi Arabian hospital settings to guide the selection of effective treatment strategies and prevent the spread of antimicrobial resistance. Further studies on phenotypic-resistance testing in correlation with detailed genomic analysis are necessary to fully elucidate the resistance mechanisms and the clinical significance. The genome of *A. baumannii* IRMCBCU95U exhibits a diverse set of mobile genetic elements and insertion sequences. The presence of specific MGEs (ISAba1 and IS26) associated with resistance genes highlight the importance of genomic surveillance for monitoring the emergence and spread of multidrug-resistant *A. baumannii* strains. Several potential virulence-associated genes in the *A. baumannii* IRMCBCU95U genome provide valuable insights into the genetic determinants of its pathogenicity, including iron acquisition, motility and transcriptional regulation. Further research is necessary to elucidate the mechanisms and functional consequences of the observed genetic exchange. The presence of pathogenic proteins confirmed the pathogenicity of *Acinetobacter* IRMCBCU95U, and it was predicted to be a human pathogen. The identification of an IncFIB(pNDM-Mar) plasmid fragment in *A. baumannii* IRMCBCU95U emphasizes the importance of horizontal gene transfer in the spread of antimicrobial resistance across bacterial species and suggests that this strain may possess the capacity to acquire resistance genes. However, further investigation is needed to elucidate the complete structure and context of this plasmid within the genome.

## Figures and Tables

**Table 1:** Antibiotic susceptibility profile of extensively drug-resistant *A. baumannii* IRMCBCU95U.

Antibiotic	Interpretation	MIC (µg/mL)
Cefepime	Resistant	NaN
Ceftazidime	Resistant	NaN
Ciprofloxacin	Resistant	NaN
Imipenem	Resistant	NaN
Meropenem	Resistant	NaN
Piperacillin/Tazobactam	Resistant	NaN
Tigecycline	Sensitive	2.0
Trimethoprim/Sulfamethoxazole	Sensitive	NaN

MIC: minimum inhibitory concentration; NaN: MIC value was not determined.

**Table 2:** Acquired AMR genes identified in *A. baumannii* IRMCBCU95U.

Resistance Gene	Identity (%)	Alignment Length	Position in Reference	Contig or Depth	Position in Contig	Resistant Phenotype	Notes
*aph*(*3′*)-*Ia*	99.51	818	1...816	contig_25_segment0 contig_25:1.0-336501.0	85910...86726	(‘kanamycin’, ‘neomycin’, ‘lividomycin’, ‘paromomycin’, ‘ribostamycin’)	
*armA*	99.35	774	1...772	contig_5_segment0 contig_5:1.0-977170.0	774608...775381	(‘gentamicin’, ‘tobramycin’, ‘amikacin’, ‘isepamicin’, ‘netilmicin’)	
bla_OXA-66_	99.76	825	1...824	contig_2_segment0 contig_2:1.0-102924.0	85778...86602	(‘unknown beta-lactam’)	Class D; OXA-51-like; naturally occurring in *A. baumannii*
bla_TEM-1D_	99.77	863	1...861	contig_25_segment0 contig_25:1.0-336501.0	79447...80309	(‘amoxicillin’, ‘ampicillin’, ‘piperacillin’, ‘ticarcillin’, ‘cephalothin’)	Class A
bla_ADC-25_	99.57	1153	1...1152	contig_5_segment2 contig_5:979486.0-1449651.0	20168...21319	(‘unknown beta-lactam’)	Chromosomal
bla_OXA-23_	99.88	822	1...822	contig_6_segment1 contig_6:253978.0-804087.0	537440...538260	(‘imipenem’, ‘meropenem’)	Class D; OXA-23-like; naturally occurring in Acinetobacter radioresistens; alternative name: blaARI-1
bla_OXA-422_	99.88	822	1...822	contig_6_segment1 contig_6:253978.0-804087.0	537440...538260	(‘unknown beta-lactam’)	Class D; OXA-23-like; natural occurring in *A. baumannii*
*msr*(*E*)	99.80	1477	1...1476	contig_5_segment0 contig_5:1.0-977170.0	770829...772303	(‘erythromycin’, ‘azithromycin’, ‘quinupristin’, ‘pristinamycin ia’, ‘virginiamycin s’)	ABC transporter
*mph*(*E*)	99.77	886	1...885	contig_5_segment0 contig_5:1.0-977170.0	769889...770773	(‘erythromycin’)	Macrolide phosphotransferase

**Table 3:** Antimicrobial-resistance genes in IRMCBCU95U genome associated with resistance to multiple classes of antibiotics.

Template	Score	Template Length	Q Value	*p* Value	Template Coverage	Query Coverage	Depth	Depth Corr
NZ_CP091346.1 *A. baumannii* strain AB217-VUB chromosome, complete genome	137,198	152,863	137,185.02	1 × 10^26^	89.66	86.57	0.90	0.6321
*aph*(*3′*)-*Ia*_7_X62115	806	816	800.34	1 × 10^26^	100.12	99.88	1.00	0.6708
*armA*_1_AY220558	759	774	753.59	1 × 10^26^	100.26	99.74	1.00	0.6708
bla_OXA-23__1_AY795964	821	822	815.31	1 × 10^26^	99.88	100.12	1.00	0.6708
bla_OXA-66__1_AY750909	819	825	813.28	1 × 10^26^	100.12	99.88	1.00	0.6708
bla_TEM-1D__1_AF188200	858	861	852.07	1 × 10^26^	100.23	99.77	1.00	0.6708
bla_ADC-25__1_EF016355	1139	1152	1131.40	1 × 10^26^	100.00	100.00	1.00	0.6708
*msr*(*E*)_1_FR751518	1468	1476	1458.76	1 × 10^26^	99.93	100.07	1.00	0.6708
*mph*(*E*)_1_DQ839391	881	885	874.93	1 × 10^26^	100.00	100.00	1.00	0.6708

**Table 4:** Top virulence factors from standard nucleotide BLAST analysis of *A. baumannii* IRMCBCU95U genome.

VFDB ID	Gene Name/Description	Identity (%)	E-Value	Score (bits)	Length	Identities	Gaps	Strand
VFG037497	(ACICU_RS04580) TonB-dependent receptor	94	0	4811	3105	2943/3108	7/3108	+/+
VFG037513	(ACICU_RS04590) porin family protein	94	0	2268	1467	1386/1468	4/1468	+/+
VFG050400	(pilU) PilT/Pilu family type 4a pilus ATPase	99	0	2141	1119	1108/1119	1/1119	+/−
VFG050386	(pilT) type IV pilus twitching motility protein PilT	99	0	2030	1038	1038/1040	2/1040	+/−
VFG037490	(ACICU_RS04575) FecR domain-containing protein	94	0	1584	1017	966/1020	3/1020	+/+
VFG037470	(ACICU_RS04565) LysR family transcriptional regulator	94	0	1392	891	839/887	N/A	+/+
VFG037505	(ACICU_RS04585) transferrin-binding protein-like	96	0	1332	792	763/792	1/792	+/+
VFG037521	(ACICU_RS04595) TonB family protein	92	0	1279	921	853/918	5/918	+/+
VFG037529	(hemo) biliverdin-producing heme oxygenase	97	0	1035	600	585/602	3/602	+/+
VFG037482	(ACICU_RS04570) sigma-70 family RNA polymerase sigma factor	97	0	924	522	508/522	N/A	+/+

## Data Availability

The original contributions presented in the study are included in the article/Supplementary Materials. Further inquiries can be directed to the corresponding author.

## Supplementary Materials

The following supporting information can be downloaded at: https://www.mdpi.com/article/10.3390/life15071094/s1. Figure S1: Genome map of *Acinetobacter* IRMCBCU95U genome, with selected genomic regions projected to indicate the location of mutated genes; Figure S2. *Acinetobacter* IRMCBCU95U Genome map with selected genomic regions indicating the location of mutated genes associated with Mupirocin (Top panel), amoxicillin (Middle panel) and rifampicin (Bottom panel); Table S1: Comprehensive overview of the quality of the sequencing data generated from the IRMCBCU95U sample using the NanoForms platform.; Table S2: Assembly statistics for the IRMCBCU95U genome; Table S3: List of genomes used for constructing phylogenetic tree of whole genome of IRMCBCU95U; Table S4: Genome tree analysis statistics for constructing phylogenetic tree of whole genome of IRMCBCU95U; Table S5: Genome-predicted resistance phenotype in extensively drug-resistant *Acinetobacter baumannii* IRMCBCU95U; Table S6: List of mobile elements identified in extensively drug-resistant *Acinetobacter baumannii* IRMCBCU95U; Table S7: Pathogenic Proteins identified in the genome IRMCBCU95U; Table S8: Fragment of plasmid sequence identified in IRMCBCU95U.
