# Supplementary material for: Hybrid Genome and Clinical Impact of Emerging Extensively Drug-Resistant Priority Bacterial Pathogen Acinetobacter baumannii in Saudi Arabia
Source: Life (Basel). 2025 Jul 12;15(7):1094. doi: 10.3390/life15071094 (PMC12300311; doi:10.3390/life15071094)
Supplement: Supplementary file 1 [file life-15-01094-s001.zip › life-3717760-supplementary.pdf]

Supplementary Table S1: Comprehensive overview of the quality of the sequencing data generated from the IRMCBCU95U sample using the NanoForms platform.

Mean read length	2,665.9
Mean read quality	10.7
Median read length	1,040.0
Median read quality	10.8
Number of reads	33,004.0
Read length N50	7,677.0
Total bases	87,986,861.0
Number, percentage and megabases of reads above quality cutoffs	
>Q5	33004 (100.0%) 88.0Mb
>Q7	33000 (100.0%) 88.0Mb
>Q10	21510 (65.2%) 66.5Mb
>Q12	7947 (24.1%) 34.7Mb
>Q15	85 (0.3%) 0.3Mb
Top 5 highest mean basecall quality scores and their read lengths	
1	17.1 (1083)
2	16.6 (110)
3	16.6 (1822)
4	16.5 (1052)
5	16.5 (951)
Top 5 longest reads and their mean basecall quality score	
1	126801 (9.7)
2	114194 (9.8)
3	101489 (8.2)
4	100288 (9.2)
5	95882 (8.4)

Supplementary Table S2: Assembly statistics for the IRMCBCU95U genome.

Statistics without reference	assembly	polished_genome	consensus
# contigs	27	27	41
# contigs (>= 0 bp)	27	27	45
# contigs (>= 1000 bp)	27	27	38
# contigs (>= 5000 bp)	26	26	29
# contigs (>= 10000 bp)	21	21	23
# contigs (>= 25000 bp)	21	20	21
# contigs (>= 50000 bp)	13	12	14
Largest contig	1453532	1449342	978253
Total length	4371541	4325280	4299246
Total length (>= 0 bp)	4371541	4325280	4300495
Total length (>= 1000 bp)	4371541	4325280	4297201
Total length (>= 5000 bp)	4366947	4320737	4279782
Total length (>= 10000 bp)	4332487	4286386	4237752
Total length (>= 25000 bp)	4332487	4262391	4194468
Total length (>= 50000 bp)	4049474	3972242	3930832
N50	807720	803706	405948
N75	167919	167146	166297
L50	2	2	4
L75	7	7	9
GC (%)	40.64	40.43	40.47
Mismatches			
# N's	0	0	0
# N's per 100 kbp	0	0	0

Supplementary Table S3: List of genomes used for constructing phylogenetic tree of whole genome of IRMCBCU95U.

**GenomeId**	**Total Genes**	**Single Copy**	**Used**	**Name**
2605383.3	1668	215	100	*Acinetobacter* sp. B10A
2605267.3	1717	215	100	*Acinetobacter* sp. FNA3
2605270.3	1736	215	100	*Acinetobacter* sp. SCC477
2605272.3	1737	215	100	*Acinetobacter* sp. EC24
2665161.17	1765	215	100	*Acinetobacter faecalis* ANC 7521
2605271.3	1772	215	100	Acinetobacter sp. EC115
2662362.3	1856	215	100	*Acinetobacter* sp. dk386
281375.3	1865	215	100	*Acinetobacter marinus* strain ANC 3699
2696476.3	1939	215	100	*Acinetobacter* sp. B301
1229165.4	1987	215	100	*Acinetobacter* apis strain ANC 5114
2715163.3	2021	215	100	*Acinetobacter* sp. 185
2715164.3	2106	215	100	*Acinetobacter* sp. 323-1
1392540.3	2137	215	100	*Acinetobacter nectaris* CIP 110549
202956.26	2148	215	100	*Acinetobacter towneri* strain GX3
1219383.3	2197	215	100	*Acinetobacter boissieri* strain ANC 4422
1353941.3	2213	215	100	*Acinetobacter harbinensis* HITLi 7
2136182.11	2231	215	100	*Acinetobacter cumulans* WCHAc060092
2576829.3	2256	215	100	*Acinetsobacter* sp. S23
2419603.3	2297	215	100	*Acinetobacter* sp. WCHAc060012
2483802.3	2330	215	100	*Acinetobacter* sp. Marseille-P8049
2420890.3	2330	215	100	*Acinetobacter* sp. WCHAc060005
2053287.22	2338	215	100	*Acinetobacter pseudolwoffii* WB5
2529843.3	2339	215	100	*Acinetobacter* sp. ANC 4471
756892.66	2342	215	100	*Acinetobacter indicus* strain TQ23
981333.9	2352	215	100	*Acinetobacter parvus* DSM 16617 = CIP 108168
1673609.3	2387	215	100	*Acinetobacter albensis* strain ANC 4874

1226327.14	2389	215	100	*Acinetobacter kookii* JCM 18512
40216.65	2393	215	100	*Acinetobacter radioresistens* strain LH6
28090.21	2396	215	100	*Acinetobacter lwoffii* strain Acilw_F78
2609668.3	2410	215	100	*Acinetobacter* sp. C16S1
1262585.11	2429	215	100	*Acinetobacter qingfengensis* strain CCUG 69710T
1324350.3	2432	215	100	*Acinetobacter* sp. 114
1752076.3	2432	215	100	*Acinetobacter halotolerans* strain JCM 31009
2762215.3	2436	215	100	*Acinetobacter* sp. Sa1BUA6
2126983.3	2437	215	100	*Acinetobacter* sp. KPC-SM-21
70346.7	2450	215	100	*Acinetobacter variabilis* strain RYU24
1843371.3	2455	215	100	*Acinetobacter* sp. Ac_1271
1226327.3	2472	215	100	*Acinetobacter kookii* strain ANC 4667
108981.23	2476	215	100	*Acinetobacter schindleri* strain HZE30-1
1977882.3	2482	215	100	*Acinetobacter* sp. ANC 4999 strain ANC 4999
2699469.4	2489	215	100	*Acinetobacter* sp. PS-1
2943497.3	2494	215	100	*Acinetobacter* sp. Y-23
2419602.3	2497	215	100	*Acinetobacter* sp. WCHAc060096
70346.34	2502	215	100	*Acinetobacter variabilis* TB2-2B
29430.59	2519	215	100	*Acinetobacter haemolyticus* strain HW-2A
1341683.3	2523	215	100	*Acinetobacter brisouii* CIP 110357
40215.126	2555	215	100	*Acinetobacter junii* strain YR7
2998078.4	2555	215	100	*Acinetobacter* sp. VNH17
1873711.4	2562	215	100	*Acinetobacter* sp. ANC 4603
1443941.3	2567	215	100	*Acinetobacter gandensis* strain ANC 4275
28090.135	2600	215	100	*Acinetobacter lwoffii* strain H7
202954.8	2647	215	100	*Acinetobacter tandoii* strain W4-4-4
487316.69	2664	215	100	*Acinetobacter soli* strain M3-1-68
202951.7	2680	215	100	*Acinetobacter bouvetii* strain JCM 18991
1977878.3	2701	215	100	*Acinetobacter* sp. ANC 4655 strain ANC 4655
1217715.3	2709	215	100	*Acinetobacter* sp. ANC 3994
62977.6	2717	215	100	*Acinetobacter* sp. ADP1
1217711.3	2724	215	100	*Acinetobacter* sp. NIPH 236

1217648.3	2733	215	100	*Acinetobacter beijerinckii* CIP 110307
1776740.3	2739	215	100	*Acinetobacter modestus* strain CCM 8639
1191460.12	2740	215	100	*Acinetobacter venetianus* RAG-1 = CIP 110063
2006115.3	2770	215	100	*Acinetobacter* sp. LW15
1217716.3	2781	215	100	*Acinetobacter ursingii* NIPH 706
1343071.3	2794	215	100	*Acinetobacter nosocomialis* M2
1217662.6	2796	215	100	*Acinetobacter johnsonii* ANC 3681
1096779.7	2804	215	100	*Acinetobacter puyangensis* JCM 18011
2419601.3	2812	215	100	*Acinetobacter* sp. WCHAc060115
871585.3	2833	215	100	*Acinetobacter calcoaceticus* PHEA-2
465797.3	2845	215	100	*Acinetobacter septicus* strain FDAARGOS_1401
1217696.3	2851	215	100	*Acinetobacter* sp. NIPH 2036
1789224.3	2857	215	100	*Acinetobacter* sp. BRTC-1
1582270.3	2858	215	100	*Acinetobacter populi* strain PBJ7
1785128.9	2866	215	100	*Acinetobacter lactucae* strain QL-1
70348.3	2880	215	100	*Acinetobacter dispersus* strain NCCP 16014
1096779.3	2891	215	100	*Acinetobacter puyangensis* strain ANC 4466
280147.4	2898	215	100	*Acinetobacter courvalinii* strain CCM 8635
1530123.84	2905	215	100	*Acinetobacter seifertii* strain S21
202955.4	2912	215	100	*Acinetobacter tjernbergiae* strain DSM 14971
470.9250	2916	215	100	*Acinetobacter baumannii* strain K09-14
2730922.3	2918	215	100	*Acinetobacter* sp. J00019
421052.3	2925	215	100	*Acinetobacter rudis* CIP 110305
1776742.3	2946	215	100	*Acinetobacter vivianii* strain CCM 8642
471.46	2946	215	100	*Acinetobacter calcoaceticus* strain NCTC12983
595670.3	2954	215	100	*Acinetobacter kyonggiensis* strain ANC 5109
1871111.9	2966	215	100	*Acinetobacter defluvii* WCHA30
1879050.10	2970	215	100	*Acinetobacter wuhouensis* WCHA60
470.11567	2978	215	100	*Acinetobacter baumannii* strain ATCC 19606
471.28	3023	215	100	*Acinetobacter calcoaceticus* strain CA16
469.3674	3026	215	100	*Acinetobacter* IRMCBCU95U
436717.3	3027	215	100	*Acinetobacter oleivorans* DR1

2004650.3	3064	215	100	*Acinetobacter* sp. WCHA5 strain WCHA5
1217713.3	3108	215	100	*Acinetobacter* sp. NIPH 809
1217701.3	3138	215	100	*Acinetobacter* sp. ANC 3880
1806892.3	3140	215	100	*Acinetobacter* sp. ANC 4149
1120926.3	3304	215	100	*Acinetobacter gerneri* DSM 14967 = CIP 107464
134534.13	3344	215	100	*Acinetobacter gyllenbergii* strain FMP01
106648.100	3347	215	100	*Acinetobacter bereziniae* strain GD03185
106649.36	3432	215	100	*Acinetobacter guillouiae* NBRC 110550
2136183.5	3539	215	100	*Acinetobacter sichuanensis* WCHAc060041

Supplementary Table S4: Genome tree analysis statistics for constructing phylogenetic tree of whole genome of IRMCBCU95U.

Requested genomes	99
Genomes with data	99
Max allowed deletions	0
Max allowed duplications	0
Single-copy genes requested	100
Single-copy genes found	100
Num protein alignments	100
Alignment program	mafft
Protein alignment time	1079.6 seconds
Num aligned amino acids	31071
Num CDS alignments	100
Num aligned nucleotides	93213
Best protein model found by RAxML	LG
Branch support method	RAxML Fast Bootstrapping
RAxML likelihood	-3899556.2663
RAxML version	8.2.12
RAxML time	35353.8 seconds
Total time	36620.5 seconds

Supplementary Table S5: Genome-predicted resistance phenotype in extensively drug-resistant *Acinetobacter baumannii* IRMCBCU95U.

Antimicrobial	Class	WGS-predicted phenotype	Genetic background
gentamicin	aminoglycoside	Resistant	armA;;1;;AY220558
tobramycin	aminoglycoside	Resistant	armA;;1;;AY220558
streptomycin	aminoglycoside	No resistance	
amikacin	aminoglycoside	Resistant	armA;;1;;AY220558
isepamicin	aminoglycoside	Resistant	armA;;1;;AY220558
dibekacin	aminoglycoside	No resistance	
kanamycin	aminoglycoside	Resistant	aph(3')-Ia;;7;;X62115
neomycin	aminoglycoside	Resistant	aph(3')-Ia;;7;;X62115
lividomycin	aminoglycoside	Resistant	aph(3')-Ia;;7;;X62115
paromomycin	aminoglycoside	Resistant	aph(3')-Ia;;7;;X62115
ribostamycin	aminoglycoside	Resistant	aph(3')-Ia;;7;;X62115
unknown aminoglycoside	aminoglycoside	No resistance	
butiromycin	aminoglycoside	No resistance	
butirosin	aminoglycoside	No resistance	
hygromycin	aminoglycoside	No resistance	
netilmicin	aminoglycoside	Resistant	armA;;1;;AY220558
apramycin	aminoglycoside	No resistance	
sisomicin	aminoglycoside	No resistance	
arbekacin	aminoglycoside	No resistance	
kasugamycin	aminoglycoside	No resistance	
astromicin	aminoglycoside	No resistance	
fortimicin	aminoglycoside	No resistance	
spectinomycin	aminocyclitol	No resistance	
fluoroquinolone	quinolone	No resistance	
ciprofloxacin	quinolone	No resistance	
unknown quinolone	quinolone	No resistance	

nalidixic acid	quinolone	No resistance	
amoxicillin	beta-lactam	Resistant	blaTEM-1D;;1;;AF188200
amoxicillin+clavulanic acid	beta-lactam	No resistance	
ampicillin	beta-lactam	Resistant	blaTEM-1D;;1;;AF188200
ampicillin+clavulanic acid	beta-lactam	No resistance	
cefepime	beta-lactam	No resistance	
cefixime	beta-lactam	No resistance	
cefotaxime	beta-lactam	No resistance	
cefoxitin	beta-lactam	No resistance	
ceftazidime	beta-lactam	No resistance	
ertapenem	beta-lactam	No resistance	
imipenem	beta-lactam	Resistant	blaOXA-23;;1;;AY795964
meropenem	beta-lactam	Resistant	blaOXA-23;;1;;AY795964
piperacillin	beta-lactam	Resistant	blaTEM-1D;;1;;AF188200
piperacillin+tazobactam	beta-lactam	No resistance	
unknown beta-lactam	beta-lactam	Resistant	blaOXA-66;;1;;AY750909
aztreonam	beta-lactam	No resistance	
cefotaxime+clavulanic acid	beta-lactam	No resistance	
temocillin	beta-lactam	No resistance	
ticarcillin	beta-lactam	Resistant	blaTEM-1D;;1;;AF188200
ceftazidime+avibactam	beta-lactam	No resistance	
penicillin	beta-lactam	No resistance	
ceftriaxone	beta-lactam	No resistance	
ticarcillin+clavulanic acid	beta-lactam	No resistance	
cephalothin	beta-lactam	Resistant	blaTEM-1D;;1;;AF188200
piperacillin+clavulanic acid	beta-lactam	No resistance	
ceftiofur	under_development	No resistance	
sulfamethoxazole	folate pathway antagonist	No resistance	
trimethoprim	folate pathway	No resistance	

	antagonist		
fosfomycin	fosfomycin	No resistance	
vancomycin	glycopeptide	No resistance	
teicoplanin	glycopeptide	No resistance	
bleomycin	glycopeptide	No resistance	
lincomycin	lincosamide	No resistance	
clindamycin	lincosamide	No resistance	
dalfopristin	streptogramin a	No resistance	
pristinamycin iia	streptogramin a	No resistance	
virginiamycin m	streptogramin a	No resistance	
quinupristin+dalfopristin	streptogramin a	No resistance	
tiamulin	pleuromutilin	No resistance	
carbomycin	macrolide	No resistance	
erythromycin	macrolide	Resistant	msr(E);;1;;FR751518
azithromycin	macrolide	Resistant	msr(E);;1;;FR751518
oleandomycin	macrolide	No resistance	
spiramycin	macrolide	No resistance	
tylosin	macrolide	No resistance	
telithromycin	macrolide	No resistance	
tetracycline	tetracycline	No resistance	
doxycycline	tetracycline	No resistance	
minocycline	tetracycline	No resistance	
tigecycline	tetracycline	No resistance	
quinupristin	streptogramin b	Resistant	msr(E);;1;;FR751518
pristinamycin ia	streptogramin b	Resistant	msr(E);;1;;FR751518
virginiamycin s	streptogramin b	Resistant	msr(E);;1;;FR751518
linezolid	oxazolidinone	No resistance	
chloramphenicol	amphenicol	No resistance	
florfenicol	amphenicol	No resistance	

colistin	polymyxin	No resistance	
fusidic acid	steroid antibacterial	No resistance	
mupirocin	pseudomonic acid	No resistance	
rifampicin	rifamycin	No resistance	
metronidazole	nitroimidazole	No resistance	
narasin	ionophores	No resistance	
salinomycin	ionophores	No resistance	
maduramicin	ionophores	No resistance	

Supplementary Table S6: List of mobile elements identified in extensively drug-resistant *Acinetobacter baumannii* IRMCBCU95U.

mge_ no	name	prediction method	type	allele_len	identity	coverage	gaps	Substitution	contig	start	stop	cigar
1	ISAba1	alignment to reference	Insertion sequence	1181	0.984822934	0.990677966	11	7	contig_13_segment0 contig_13:1.0-188277.0	177969	179149	M589 D2 M11 I1 M10 I1 M26 D1 M1 D1 M19 D1 M33 I1 M76 D1 M399 I1 M1 I1 M10
3	ISAba1	alignment to reference	Insertion sequence	1182	0.998307953	0.998305085	2	0	contig_16_segment0 contig_16:1.0-404126.0	25450	26631	M991 D2 M189
4	ISAba1	alignment to reference	Insertion sequence	1182	0.996618766	0.996610169	4	0	contig_16_segment0 contig_16:1.0-404126.0	187352	188533	M263 D1 M428 I1 M299 D1 M125 D1 M64
5	ISAba1	alignment to reference	Insertion sequence	1182	0.998307953	0.998305085	2	0	contig_16_segment0 contig_16:1.0-404126.0	157542	158723	M991 D2 M189
9	IS26*	alignment to reference	Insertion sequence	819	0.995127893	0.996341463	3	1	contig_25_segment0 contig_25:1.0-336501.0	86794	87612	M109 I1 M96 D1 M517 I1 M96
10	IS26**	alignment to reference	Insertion sequence	821	0.998781973	0.998780488	1	0	contig_25_segment0 contig_25:1.0-336501.0	77926	78746	M106 D1 M714
11	IS26***	alignment to reference	Insertion sequence	820	1	1	0	0	contig_25_segment0 contig_25:1.0-336501.0	80328	81147	M820
12	ISAba1	alignment to reference	Insertion sequence	1183	0.996618766	0.997457627	3	1	contig_25_segment0 contig_25:1.0-336501.0	273743	274925	M84 D1 M105 D1 M217 D1 M774
13	ISAba1	alignment to reference	Insertion sequence	1182	0.996618766	0.996610169	4	0	contig_25_segment0 contig_25:1.0-336501.0	65370	66551	M56 D1 M542 I1 M175 D1 M217 D1 M189
14	ISAba1	alignment to reference	Insertion sequence	1182	0.996618766	0.996610169	4	0	contig_25_segment0 contig_25:1.0-336501.0	109362	110543	M84 D1 M105 D1 M129 D1 M170 I1 M691
15	ISAba1	alignment to reference	Insertion sequence	1180	1	1	0	0	contig_25_segment0 contig_25:1.0-336501.0	263714	264893	M1180
20	ISAba24	alignment to reference	Insertion sequence	2421	0.999174236	0.999173895	2	0	contig_5_segment0 contig_5:1.0-977170.0	766866	769286	M392 I1 M381 D1 M1647
21	ISAba1	alignment to reference	Insertion sequence	1181	0.997461929	0.997457627	3	0	contig_5_segment0 contig_5:1.0-977170.0	197076	198256	M84 D1 M105 D1 M158 I1 M832
22	ISAba1	alignment to reference	Insertion sequence	1181	0.994077834	0.997457627	3	4	contig_5_segment0 contig_5:1.0-977170.0	138727	139907	M189 D1 M299 I1 M221 D1 M470

23	ISAba1	alignment to reference	Insertion sequence	1177	0.997457627	0.997457627	3	0	contig_5_segment0 contig_5:1.0-977170.0	908745	909921	M53 I1 M434 I1 M320 I1 M370
24	ISAba1	alignment to reference	Insertion sequence	1181	0.994928149	0.995762712	5	1	contig_5_segment0 contig_5:1.0-977170.0	242064	243244	M56 D1 M314 I1 M403 D1 M217 D1 M11 I1 M177
25	ISAba1	alignment to reference	Insertion sequence	1182	0.998307953	0.998305085	2	0	contig_5_segment0 contig_5:1.0-977170.0	629544	630725	M189 D1 M373 D1 M618
26	ISAba1	alignment to reference	Insertion sequence	1180	0.99408284	0.994915254	6	1	contig_5_segment0 contig_5:1.0-977170.0	563	1742	M484 D1 M133 I1 M73 I1 M69 I1 M229 D2 M189
27	ISAba1	alignment to reference	Insertion sequence	1178	0.98902027	0.991525424	10	3	contig_5_segment0 contig_5:1.0-977170.0	615665	616842	M189 D2 M82 I1 M75 I1 M129 D1 M11 I1 M324 I1 M23 D1 M321 I2 M20
28	ISAba1	alignment to reference	Insertion sequence	1182	0.996618766	0.996610169	4	0	contig_5_segment0 contig_5:1.0-977170.0	216354	217535	M31 D1 M232 D1 M428 I1 M299 D1 M189
29	ISAba1	alignment to reference	Insertion sequence	1180	0.986475063	0.994915254	6	10	contig_5_segment0 contig_5:1.0-977170.0	642274	643453	M189 D2 M284 D1 M15 I1 M669 I2 M20
33	ISAba1	alignment to reference	Insertion sequence	1179	0.99661304	0.997457627	3	1	contig_5_segment2 contig_5:979486.0-1449651.0	18980	20158	M84 D1 M404 I1 M324 I1 M366
34	ISAba1	alignment to reference	Insertion sequence	1180	0.995769882	0.996610169	4	1	contig_5_segment2 contig_5:979486.0-1449651.0	120776	121955	M18 I1 M413 D1 M259 I1 M299 D1 M189
35	ISAba1	alignment to reference	Insertion sequence	1178	0.99322608	0.996610169	4	4	contig_5_segment2 contig_5:979486.0-1449651.0	157117	158294	M189 D1 M217 I1 M81 I1 M324 I1 M366
36	ISAba1	alignment to reference	Insertion sequence	1181	0.99323181	0.997457627	3	5	contig_5_segment2 contig_5:979486.0-1449651.0	216751	217931	M189 D1 M402 I1 M530 D1 M58
37	ISAba26	alignment to reference	Insertion sequence	1319	0.993181818	0.997723824	3	6	contig_5_segment2 contig_5:979486.0-1449651.0	117195	118513	M97 D1 M262 D1 M374 I1 M584
38	ISAba1	alignment to reference	Insertion sequence	1177	0.9957663	0.995762712	5	0	contig_6_segment0 contig_6:1.0-253956.0	106966	108142	M183 I1 M5 D1 M973 I3 M15
39	ISAba1	alignment to reference	Insertion sequence	1181	0.99745978	0.999152542	1	2	contig_6_segment0 contig_6:1.0-253956.0	57753	58933	M189 D1 M991
44	Tn2006	alignment to reference	Composite transposon	4807	0.998128509	0.998751301	6	3	contig_6_segment1 contig_6:253978.0-804087.0	536226	541032	M189 D1 M299 I1 M762 I1 M837 D1 M2154 D1 M373 D1 M189
45	ISAba1	alignment to reference	Insertion sequence	1182	0.994923858	0.998305085	2	4	contig_6_segment1 contig_6:253978.0-	539851	541032	M618 D1 M373 D1 M189

									804087.0			
47	ISAba26	alignment to reference	Insertion sequence	1313	0.992412747	0.996206373	5	5	contig_6_segment1 contig_6:253978.0-804087.0	291946	293258	M158 I1 M176 I1 M246 I1 M1 I1 M490 I1 M242
49	ISAba1	alignment to reference	Insertion sequence	1182	0.998307953	0.998305085	2	0	contig_8_segment0 contig_8:1.0-156175.0	72666	73847	M261 D1 M301 D1 M618
50	ISAba1	alignment to reference	Insertion sequence	1171	0.981418919	0.98559322	17	5	contig_8_segment0 contig_8:1.0-156175.0	106248	107418	M12 I1 M174 D1 M2 I1 M105 I2 M29 I1 M39 I1 M162 D1 M237 I1 M8 I2 M13 I1 M41 I1 M29 D1 M50 I1 M46 I1 M31 D1 M189
6_4_5	cn_30992_ISAba1	inferred	Composite transposon	30992	0.998307953	0.998305085	2	0	contig_16_segment0 contig_16:1.0-404126.0	157541	188533	-
12_10_11	cn_3222_IS26	inferred	Composite transposon	3222	0.998781973	0.998780488	1	0	contig_25_segment0 contig_25:1.0-336501.0	77925	81147	-
13_11	cn_5210_IS26	inferred	Composite transposon	5210	1	1	0	0	contig_25_segment0 contig_25:1.0-336501.0	80327	85537	-
14_9	cn_2898_IS26	inferred	Composite transposon	2898	0.995127893	0.996341463	3	1	contig_25_segment0 contig_25:1.0-336501.0	84714	87612	-
16_13_14	cn_45174_ISAba1	inferred	Composite transposon	45174	0.996618766	0.996610169	4	0	contig_25_segment0 contig_25:1.0-336501.0	65369	110543	-
17_12_15	cn_11212_ISAba1	inferred	Composite transposon	11212	1	1	0	0	contig_25_segment0 contig_25:1.0-336501.0	263713	274925	-
30_21_28	cn_20460_ISAba1	inferred	Composite transposon	20460	0.997461929	0.997457627	3	0	contig_5_segment0 contig_5:1.0-977170.0	197075	217535	-
31_24_28	cn_26891_ISAba1	inferred	Composite transposon	26891	0.996618766	0.996610169	4	0	contig_5_segment0 contig_5:1.0-977170.0	216353	243244	-
32_25_27	cn_15061_ISAba1	inferred	Composite transposon	15061	0.98902027	0.991525424	10	3	contig_5_segment0 contig_5:1.0-977170.0	615664	630725	-

33_25_29	cn_13910_ISAba1	inferred	Composite transposon	13910	0.998307953	0.998305085	2	0	contig_5_segment0 contig_5:1.0-977170.0	629543	643453	-
37_34_35	cn_37519_ISAba1	inferred	Composite transposon	37519	0.995769882	0.996610169	4	1	contig_5_segment2 contig_5:979486.0-1449651.0	120775	158294	-
40_38_39	cn_50390_ISAba1	inferred	Composite transposon	50390	0.99745978	0.999152542	1	2	contig_6_segment0 contig_6:1.0-253956.0	57752	108142	-
46_45	cn_4807_ISAba1	inferred	Composite transposon	4807	0.994923858	0.998305085	2	4	contig_6_segment1 contig_6:253978.0-804087.0	536225	541032	-
51_49_50	cn_34753_ISAba1	inferred	Composite transposon	34753	0.998307953	0.998305085	2	0	contig_8_segment0 contig_8:1.0-156175.0	72665	107418	-

* synonyms: IS160;IS26L;IS26R;IS6;IS140;IS46; **: IS160;IS26L;IS26R;IS6;IS140;IS46; ***: IS160;IS26L;IS26R;IS6;IS140;IS46

Supplementary Table S7: Pathogenic Proteins identified in the genome IRMCBCU95U.

**Input Sequence**	fig|469.3674.peg.6943 Exodeoxyribonuclease V beta chain (EC 3.1.11.5) [*Acinetobacter* IRMCBCU95U | 469.3674]						
	PROJECT ID	ACCESSION ID	ORGANISMS	CLASS	PROTEIN FUNCTION	PROTEIN ID	%IDENTITY
**Matched Family**	17827	CP000863	*Acinetobacter baumannii* ACICU, complete genome.	Gammaproteobacteria	ATP-dependent exoDNAse (exonuclease V) beta subunit	ACC55681	100.0

**Input Sequence**	fig|469.3674.peg.6365 Two-component system sensor histidine kinase [*Acinetobacter* IRMCBCU95U | 469.3674]						
	PROJECT ID	ACCESSION ID	ORGANISMS	CLASS	PROTEIN FUNCTION	PROTEIN ID	%IDENTITY
**Matched Family**	17477	CP000521	*Acinetobacter baumannii* ATCC 17978, complete genome.	Gammaproteobacteria	putative two-component sensor	ABO13691	100.0

**Input Sequence**	fig|469.3674.peg.1265 hypothetical protein [*Acinetobacter* IRMCBCU95U | 469.3674]						
	PROJECT ID	ACCESSION ID	ORGANISMS	CLASS	PROTEIN FUNCTION	PROTEIN ID	%IDENTITY
**Matched Family**	17827	CP000863	*Acinetobacter baumannii* ACICU, complete genome.	Gammaproteobacteria	uncharacterized protein conserved in bacteria	ACC55996	100.0

**Input Sequence**	fig|469.3674.peg.1065 Signal transduction histidine-protein kinase BarA (EC 2.7.13.3) [*Acinetobacter* IRMCBCU95U | 469.3674]

	PROJECT ID	ACCESSION ID	ORGANISMS	CLASS	PROTEIN FUNCTION	PROTEIN ID	%IDENTITY
**Matched Family**	13001	XXX	Proteus mirabilis HI4320	Gammaproteobacteria	XXX		100.0

**Input Sequence**	fig|469.3674.peg.4257 Outer membrane component of TAM transport system [*Acinetobacter* IRMCBCU95U | 469.3674]						
	PROJECT ID	ACCESSION ID	ORGANISMS	CLASS	PROTEIN FUNCTION	PROTEIN ID	%IDENTITY
**Matched Family**	17827	CP000863	*Acinetobacter baumannii* ACICU, complete genome.	Gammaproteobacteria	Outer membrane protein	ACC57644	100.0

**Input Sequence**	fig|469.3674.peg.1364 outer membrane autotransporter barrel domain [*Acinetobacter* IRMCBCU95U | 469.3674]						
	PROJECT ID	ACCESSION ID	ORGANISMS	CLASS	PROTEIN FUNCTION	PROTEIN ID	%IDENTITY
**Matched Family**	13001	XXX	Proteus mirabilis HI4320	Gammaproteobacteria	XXX		100.0

**Input Sequence**	fig|469.3674.peg.5204 DNA internalization-related competence protein ComEC/Rec2 [*Acinetobacter* IRMCBCU95U | 469.3674]						
	PROJECT ID	ACCESSION ID	ORGANISMS	CLASS	PROTEIN FUNCTION	PROTEIN ID	%IDENTITY
**Matched Family**	17827	CP000863	*Acinetobacter baumannii* ACICU, complete genome.	Gammaproteobacteria	predicted hydrolase (metallo-beta-lactamase superfamily)	ACC58166	100.0

**Input Sequence**	fig|469.3674.peg.2963 Outer membrane receptor proteins, mostly Fe transport [*Acinetobacter* IRMCBCU95U | 469.3674]						
	PROJECT ID	ACCESSION ID	ORGANISMS	CLASS	PROTEIN FUNCTION	PROTEIN ID	%IDENTITY
**Matched Family**	17477	CP000523	*Acinetobacter baumannii* ATCC 17978 plasmid pAB2, complete sequence.	Gammaproteobacteria	hypothetical protein	ABO13864	100.0

**Input Sequence**	fig|469.3674.peg.2965 Outer membrane receptor proteins, mostly Fe transport [*Acinetobacter* IRMCBCU95U | 469.3674]						
	PROJECT ID	ACCESSION ID	ORGANISMS	CLASS	PROTEIN FUNCTION	PROTEIN ID	%IDENTITY
**Matched Family**	21111	CP001183	*Acinetobacter baumannii* AB0057 plasmid pAB0057, complete sequence.	Gammaproteobacteria	TonB-dependent receptor protein	ACJ43228	100.0

**Input Sequence**	fig|469.3674.peg.983 FIG021862: membrane protein, exporter [*Acinetobacter* IRMCBCU95U | 469.3674]						
	PROJECT ID	ACCESSION ID	ORGANISMS	CLASS	PROTEIN FUNCTION	PROTEIN ID	%IDENTITY
**Matched Family**	17827	CP000863	*Acinetobacter baumannii* ACICU, complete genome.	Gammaproteobacteria	predicted exporter	ACC55840	100.0

**Input Sequence**	fig|469.3674.peg.1989 General secretion pathway protein D / Type II secretion outermembrane pore forming protein (PulD) [*Acinetobacter* IRMCBCU95U | 469.3674]						
	PROJECT ID	ACCESSION ID	ORGANISMS	CLASS	PROTEIN FUNCTION	PROTEIN ID	%IDENTITY

**Matched Family**	17477	CP000521	*Acinetobacter baumannii* ATCC 17978, complete genome.	Gammaproteobacteria	putative general secretion pathway protein	ABO10746	100.0

**Input Sequence**	fig|469.3674.peg.1988 General secretion pathway protein D / Type II secretion outermembrane pore forming protein (PulD) [*Acinetobacter* IRMCBCU95U | 469.3674]						
	PROJECT ID	ACCESSION ID	ORGANISMS	CLASS	PROTEIN FUNCTION	PROTEIN ID	%IDENTITY
**Matched Family**	17827	CP000863	*Acinetobacter baumannii* ACICU, complete genome.	Gammaproteobacteria	Type II secretory pathway, component PulD	ACC55604	100.0

**Input Sequence**	fig|469.3674.peg.911 Ferrichrome-iron receptor [*Acinetobacter* IRMCBCU95U | 469.3674]						
	PROJECT ID	ACCESSION ID	ORGANISMS	CLASS	PROTEIN FUNCTION	PROTEIN ID	%IDENTITY
**Matched Family**	17827	CP000863	*Acinetobacter baumannii* ACICU, complete genome.	Gammaproteobacteria	Outer membrane receptor for monomeric catechols	ACC55797	100.0

**Input Sequence**	fig|469.3674.peg.888 Alkaline phosphatase (EC 3.1.3.1) [*Acinetobacter* IRMCBCU95U | 469.3674]						
	PROJECT ID	ACCESSION ID	ORGANISMS	CLASS	PROTEIN FUNCTION	PROTEIN ID	%IDENTITY
**Matched Family**	13001	XXX	Proteus mirabilis HI4320	Gammaproteobacteria	XXX		100.0

Input Sequence	fig|469.3674.peg.3245 Outer membrane receptor for ferric coprogen and ferric-rhodotorulic acid [*Acinetobacter* IRMCBCU95U | 469.3674]						
	PROJECT ID	ACCESSION ID	ORGANISMS	CLASS	PROTEIN FUNCTION	PROTEIN ID	%IDENTITY
**Matched Family**	17827	CP000863	*Acinetobacter baumannii* ACICU, complete genome.	Gammaproteobacteria	Outer membrane receptor for ferric coprogen and ferric-rhodotorulic acid	ACC57008	100.0

Input Sequence	fig|469.3674.peg.3934 diguanylate cyclase/phosphodiesterase (GGDEF & EAL domains) with PAS/PAC sensor(s) [*Acinetobacter* IRMCBCU95U | 469.3674]						
	PROJECT ID	ACCESSION ID	ORGANISMS	CLASS	PROTEIN FUNCTION	PROTEIN ID	%IDENTITY
**Matched Family**	17477	CP000521	*Acinetobacter baumannii* ATCC 17978, complete genome.	Gammaproteobacteria	putative diguanylate cyclase/phosphodiesterase	ABO12376	100.0

Input Sequence	fig|469.3674.peg.5425 Probable site-specific recombinase [*Acinetobacter* IRMCBCU95U | 469.3674]						
	PROJECT ID	ACCESSION ID	ORGANISMS	CLASS	PROTEIN FUNCTION	PROTEIN ID	%IDENTITY
**Matched Family**	13001	XXX	Proteus mirabilis HI4320	Gammaproteobacteria	XXX		100.0

Input Sequence	fig|469.3674.peg.1027 GGDEF domain protein [*Acinetobacter* IRMCBCU95U | 469.3674]						
	PROJECT ID	ACCESSION ID	ORGANISMS	CLASS	PROTEIN FUNCTION	PROTEIN ID	%IDENTITY
**Matched**	17477	CP000521	*Acinetobacter*	Gammaproteobacteria	putative membrane protein	ABO10999	100.0

Family			*baumannii* ATCC 17978, complete genome.				

Input Sequence	fig|469.3674.peg.3125 Thimet oligopeptidase (EC 3.4.24.15) [*Acinetobacter* IRMCBCU95U | 469.3674]						
	PROJECT ID	ACCESSION ID	ORGANISMS	CLASS	PROTEIN FUNCTION	PROTEIN ID	%IDENTITY
Matched Family	17827	CP000863	*Acinetobacter baumannii* ACICU, complete genome.	Gammaproteobacteria	Zn-dependent oligopeptidase	ACC56948	100.0

Input Sequence	fig|469.3674.peg.5778 Outer membrane vitamin B12 receptor BtuB [*Acinetobacter* IRMCBCU95U | 469.3674]						
	PROJECT ID	ACCESSION ID	ORGANISMS	CLASS	PROTEIN FUNCTION	PROTEIN ID	%IDENTITY
Matched Family	17827	CP000863	*Acinetobacter baumannii* ACICU, complete genome.	Gammaproteobacteria	Outer membrane cobalamin receptor protein	ACC58436	100.0

Input Sequence	fig|469.3674.peg.1436 DNA primase DnaG [*Acinetobacter* IRMCBCU95U | 469.3674]						
	PROJECT ID	ACCESSION ID	ORGANISMS	CLASS	PROTEIN FUNCTION	PROTEIN ID	%IDENTITY
Matched Family	17827	CP000863	*Acinetobacter baumannii* ACICU, complete genome.	Gammaproteobacteria	DNA primase (bacterial type)	ACC56104	100.0

**Input Sequence**	fig|469.3674.peg.5059 hypothetical protein [*Acinetobacter* IRMCBCU95U | 469.3674]						
	PROJECT ID	ACCESSION ID	ORGANISMS	CLASS	PROTEIN FUNCTION	PROTEIN ID	%IDENTITY
**Matched Family**	17827	CP000863	*Acinetobacter baumannii* ACICU, complete genome.	Gammaproteobacteria	hypothetical protein	ACC58010	100.0

**Input Sequence**	fig|469.3674.peg.3120 Dipeptide ABC transporter, substrate-binding protein DppA (TC 3.A.1.5.2) [*Acinetobacter* IRMCBCU95U | 469.3674]						
	PROJECT ID	ACCESSION ID	ORGANISMS	CLASS	PROTEIN FUNCTION	PROTEIN ID	%IDENTITY
**Matched Family**	17827	CP000863	*Acinetobacter baumannii* ACICU, complete genome.	Gammaproteobacteria	ABC-type dipeptide transport system, periplasmic component	ACC56946	100.0

**Input Sequence**	fig|469.3674.peg.3198 Uncharacterized siderophore S biosynthesis protein, AcsD-like @ Siderophore synthetase superfamily, group A @ Siderophore synthetase large component, acetyltransferase [*Acinetobacter* IRMCBCU95U | 469.3674]						
	PROJECT ID	ACCESSION ID	ORGANISMS	CLASS	PROTEIN FUNCTION	PROTEIN ID	%IDENTITY
**Matched Family**	17827	CP000863	*Acinetobacter baumannii* ACICU, complete genome.	Gammaproteobacteria	Siderophore synthetase component	ACC56984	100.0

**Input Sequence**	fig|469.3674.peg.5046 Aminopeptidase N family protein, contains PDZ domain [*Acinetobacter* IRMCBCU95U | 469.3674]						
	PROJECT ID	ACCESSION ID	ORGANISMS	CLASS	PROTEIN FUNCTION	PROTEIN ID	%IDENTITY
**Matched**	17477	CP000521	*Acinetobacter*	Gammaproteobacteria	putative trypsin-like serine protease	ABO12896	100.0

Family			*baumannii* ATCC 17978, complete genome.				

Input Sequence	fig|469.3674.peg.5345 poly (glycerol-phosphate) alpha-glucosyltransferase [*Acinetobacter* IRMCBCU95U | 469.3674]						
	PROJECT ID	ACCESSION ID	ORGANISMS	CLASS	PROTEIN FUNCTION	PROTEIN ID	%IDENTITY
Matched Family	17827	CP000863	*Acinetobacter baumannii* ACICU, complete genome.	Gammaproteobacteria	hypothetical protein	ACC58249	100.0

Input Sequence	fig|469.3674.peg.4054 Nitrite reductase [NAD(P)H] large subunit (EC 1.7.1.4) [*Acinetobacter* IRMCBCU95U | 469.3674]						
	PROJECT ID	ACCESSION ID	ORGANISMS	CLASS	PROTEIN FUNCTION	PROTEIN ID	%IDENTITY
Matched Family	17827	CP000863	*Acinetobacter baumannii* ACICU, complete genome.	Gammaproteobacteria	NAD(P)H-nitrite reductase	ACC57441	100.0

Input Sequence	fig|469.3674.peg.3237 FIG138928: iron-regulated membrane protein [*Acinetobacter* IRMCBCU95U | 469.3674]						
	PROJECT ID	ACCESSION ID	ORGANISMS	CLASS	PROTEIN FUNCTION	PROTEIN ID	%IDENTITY
Matched Family	17827	CP000863	*Acinetobacter baumannii* ACICU, complete genome.	Gammaproteobacteria	uncharacterized iron-regulated membrane protein	ACC57006	100.0

Input Sequence	fig|469.3674.peg.5592 Outer membrane protein E [*Acinetobacter* IRMCBCU95U | 469.3674]						
	PROJECT ID	ACCESSION ID	ORGANISMS	CLASS	PROTEIN FUNCTION	PROTEIN ID	%IDENTITY
**Matched Family**	17827	CP000863	*Acinetobacter baumannii* ACICU, complete genome.	Gammaproteobacteria	Long-chain fatty acid transport protein	ACC58335	100.0

Input Sequence	fig|469.3674.peg.1357 hypothetical protein [*Acinetobacter* IRMCBCU95U | 469.3674]						
	PROJECT ID	ACCESSION ID	ORGANISMS	CLASS	PROTEIN FUNCTION	PROTEIN ID	%IDENTITY
**Matched Family**	17827	CP000863	*Acinetobacter baumannii* ACICU, complete genome.	Gammaproteobacteria	hypothetical protein	ACC56056	100.0

Input Sequence	fig|469.3674.peg.6203 UDP-N-acetylmuramoyl-dipeptide--2,6-diaminopimelate ligase (EC 6.3.2.13) [*Acinetobacter* IRMCBCU95U | 469.3674]						
	PROJECT ID	ACCESSION ID	ORGANISMS	CLASS	PROTEIN FUNCTION	PROTEIN ID	%IDENTITY
**Matched Family**	17477	CP000521	*Acinetobacter baumannii* ATCC 17978, complete genome.	Gammaproteobacteria	UDP-N-acetylmuramoylalanyl-D-glutamate-2 6-diaminopimelate ligase	ABO13596	100.0

Input Sequence	fig|469.3674.peg.5218 Efflux transport system, outer membrane factor (OMF) lipoprotein [*Acinetobacter* IRMCBCU95U | 469.3674]						
	PROJECT ID	ACCESSION ID	ORGANISMS	CLASS	PROTEIN FUNCTION	PROTEIN ID	%IDENTITY

Matched Family	21111	CP001182	*Acinetobacter baumannii* AB0057, complete genome.	Gammaproteobacteria	putative RND family drug transporter	ACJ42364	100.0

Input Sequence	fig|469.3674.peg.5431 Efflux transport system, outer membrane factor (OMF) lipoprotein [*Acinetobacter* IRMCBCU95U | 469.3674]						
	PROJECT ID	ACCESSION ID	ORGANISMS	CLASS	PROTEIN FUNCTION	PROTEIN ID	%IDENTITY
Matched Family	17827	CP000863	*Acinetobacter baumannii* ACICU, complete genome.	Gammaproteobacteria	Outer membrane protein	ACC58301	100.0

Input Sequence	fig|469.3674.peg.5527 FIG00349972: hypothetical protein [*Acinetobacter* IRMCBCU95U | 469.3674]						
	PROJECT ID	ACCESSION ID	ORGANISMS	CLASS	PROTEIN FUNCTION	PROTEIN ID	%IDENTITY
Matched Family	17477	CP000521	*Acinetobacter baumannii* ATCC 17978, complete genome.	Gammaproteobacteria	hypothetical protein	ABO12456	100.0

Input Sequence	fig|469.3674.peg.4735 hypothetical protein [*Acinetobacter* IRMCBCU95U | 469.3674]						
	PROJECT ID	ACCESSION ID	ORGANISMS	CLASS	PROTEIN FUNCTION	PROTEIN ID	%IDENTITY
Matched Family	21111	CP001182	*Acinetobacter baumannii* AB0057, complete genome.	Gammaproteobacteria	conserved hypothetical protein	ACJ42054	100.0

**Input Sequence**	fig|469.3674.peg.6769 FIGfam110555 [*Acinetobacter* IRMCBCU95U | 469.3674]						
	PROJECT ID	ACCESSION ID	ORGANISMS	CLASS	PROTEIN FUNCTION	PROTEIN ID	%IDENTITY
**Matched Family**	21111	CP001182	*Acinetobacter baumannii* AB0057, complete genome.	Gammaproteobacteria	conserved hypothetical protein	ACJ39982	100.0

**Input Sequence**	fig|469.3674.peg.1389 Alpha,alpha-trehalose-phosphate synthase [UDP-forming] (EC 2.4.1.15) [*Acinetobacter* IRMCBCU95U | 469.3674]						
	PROJECT ID	ACCESSION ID	ORGANISMS	CLASS	PROTEIN FUNCTION	PROTEIN ID	%IDENTITY
**Matched Family**	17477	CP000521	*Acinetobacter baumannii* ATCC 17978, complete genome.	Gammaproteobacteria	trehalose-6-phosphate synthase	ABO11241	100.0

**Input Sequence**	fig|469.3674.peg.3485 RND efflux system, outer membrane lipoprotein, NodT family [*Acinetobacter* IRMCBCU95U | 469.3674]						
	PROJECT ID	ACCESSION ID	ORGANISMS	CLASS	PROTEIN FUNCTION	PROTEIN ID	%IDENTITY
**Matched Family**	17827	CP000863	*Acinetobacter baumannii* ACICU, complete genome.	Gammaproteobacteria	Outer membrane protein	ACC57135	100.0

**Input Sequence**	fig|469.3674.peg.6973 ATP-binding protein [*Acinetobacter* IRMCBCU95U | 469.3674]						
	PROJECT ID	ACCESSION ID	ORGANISMS	CLASS	PROTEIN FUNCTION	PROTEIN ID	%IDENTITY

**Matched Family**	17827	CP000863	*Acinetobacter baumannii* ACICU, complete genome.	Gammaproteobacteria	predicted ATP-binding protein	ACC55670	100.0

**Input Sequence**	fig|469.3674.peg.3475 hypothetical protein [*Acinetobacter* IRMCBCU95U | 469.3674]						
	PROJECT ID	ACCESSION ID	ORGANISMS	CLASS	PROTEIN FUNCTION	PROTEIN ID	%IDENTITY
**Matched Family**	17827	CP000863	*Acinetobacter baumannii* ACICU, complete genome.	Gammaproteobacteria	Glycine/D-amino acid oxidase (deaminating)	ACC57129	100.0

**Input Sequence**	fig|469.3674.peg.6834 Tfp pilus assembly protein FimV [*Acinetobacter* IRMCBCU95U | 469.3674]						
	PROJECT ID	ACCESSION ID	ORGANISMS	CLASS	PROTEIN FUNCTION	PROTEIN ID	%IDENTITY
**Matched Family**	17827	CP000863	*Acinetobacter baumannii* ACICU, complete genome.	Gammaproteobacteria	hypothetical protein	ACC55748	100.0

**Input Sequence**	fig|469.3674.peg.6891 Multidrug efflux transporter MdtK/NorM (MATE family) [*Acinetobacter* IRMCBCU95U | 469.3674]						
	PROJECT ID	ACCESSION ID	ORGANISMS	CLASS	PROTEIN FUNCTION	PROTEIN ID	%IDENTITY
**Matched Family**	13001	XXX	Proteus mirabilis HI4320	Gammaproteobacteria	XXX		100.0

**Input**	fig|469.3674.peg.5170 Amidohydrolase family protein [*Acinetobacter* IRMCBCU95U | 469.3674]

Sequence							
	PROJECT ID	ACCESSION ID	ORGANISMS	CLASS	PROTEIN FUNCTION	PROTEIN ID	%IDENTITY
**Matched Family**	17827	CP000863	*Acinetobacter baumannii* ACICU, complete genome.	Gammaproteobacteria	Metal-dependent amidase/aminoacylase/carboxypeptidase	ACC58143	100.0

**Input Sequence**	fig|469.3674.peg.5453 Two-component system sensor histidine kinase [*Acinetobacter* IRMCBCU95U | 469.3674]						
	PROJECT ID	ACCESSION ID	ORGANISMS	CLASS	PROTEIN FUNCTION	PROTEIN ID	%IDENTITY
**Matched Family**	17827	CP000863	*Acinetobacter baumannii* ACICU, complete genome.	Gammaproteobacteria	Signal transduction histidine kinase	ACC58314	100.0

**Input Sequence**	fig|469.3674.peg.3526 Type I secretion outer membrane protein, TolC precursor [*Acinetobacter* IRMCBCU95U | 469.3674]						
	PROJECT ID	ACCESSION ID	ORGANISMS	CLASS	PROTEIN FUNCTION	PROTEIN ID	%IDENTITY
**Matched Family**	17827	CP000863	*Acinetobacter baumannii* ACICU, complete genome.	Gammaproteobacteria	Outer membrane protein	ACC57157	100.0

**Input Sequence**	fig|469.3674.peg.5555 replicative DNA helicase [*Acinetobacter* IRMCBCU95U | 469.3674]						
	PROJECT ID	ACCESSION ID	ORGANISMS	CLASS	PROTEIN FUNCTION	PROTEIN ID	%IDENTITY
**Matched Family**	21111	CP001182	*Acinetobacter baumannii* AB0057,	Gammaproteobacteria	replicative DNA helicase	ACJ42073	100.0

			complete genome.				

Input Sequence	fig|469.3674.peg.5049 D-alanyl-D-alanine carboxypeptidase (EC 3.4.16.4) [*Acinetobacter* IRMCBCU95U | 469.3674]						
	PROJECT ID	ACCESSION ID	ORGANISMS	CLASS	PROTEIN FUNCTION	PROTEIN ID	%IDENTITY
**Matched Family**	17477	CP000521	*Acinetobacter baumannii* ATCC 17978, complete genome.	Gammaproteobacteria	putative D-ala-D-ala-carboxypeptidase penicillin-binding protein	ABO12897	100.0

Input Sequence	fig|469.3674.peg.5459 DcaP [*Acinetobacter* IRMCBCU95U | 469.3674]						
	PROJECT ID	ACCESSION ID	ORGANISMS	CLASS	PROTEIN FUNCTION	PROTEIN ID	%IDENTITY
**Matched Family**	17827	CP000863	*Acinetobacter baumannii* ACICU, complete genome.	Gammaproteobacteria	putative DcaP-like protein	ACC58317	100.0

Input Sequence	fig|469.3674.peg.3916 Na(+)-dependent branched-chain amino acid transporter [*Acinetobacter* IRMCBCU95U | 469.3674]						
	PROJECT ID	ACCESSION ID	ORGANISMS	CLASS	PROTEIN FUNCTION	PROTEIN ID	%IDENTITY
**Matched Family**	17477	CP000521	*Acinetobacter baumannii* ATCC 17978, complete genome.	Gammaproteobacteria	branched chain amino acid transporter	ABO12368	100.0

Input Sequence	fig|469.3674.peg.949 Glycosyl transferase, group 1 family protein [*Acinetobacter* IRMCBCU95U | 469.3674]						
	PROJECT ID	ACCESSION ID	ORGANISMS	CLASS	PROTEIN FUNCTION	PROTEIN ID	%IDENTITY
**Matched Family**	17827	CP000863	*Acinetobacter baumannii* ACICU, complete genome.	Gammaproteobacteria	Glycosyltransferase	ACC55816	100.0

Input Sequence	fig|469.3674.peg.3189 Acyl-CoA dehydrogenase (EC 1.3.8.1), Mycobacterial subgroup FadE24 [*Acinetobacter* IRMCBCU95U | 469.3674]						
	PROJECT ID	ACCESSION ID	ORGANISMS	CLASS	PROTEIN FUNCTION	PROTEIN ID	%IDENTITY
**Matched Family**	13001	XXX	Proteus mirabilis HI4320	Gammaproteobacteria	XXX		100.0

Input Sequence	fig|469.3674.peg.6388 3-deoxy-D-manno-octulosonic acid transferase (EC 2.4.99.12)(EC 2.4.99.13) [*Acinetobacter* IRMCBCU95U | 469.3674]						
	PROJECT ID	ACCESSION ID	ORGANISMS	CLASS	PROTEIN FUNCTION	PROTEIN ID	%IDENTITY
**Matched Family**	13001	XXX	Proteus mirabilis HI4320	Gammaproteobacteria	XXX		100.0

Input Sequence	fig|469.3674.peg.3737 hypothetical protein [*Acinetobacter* IRMCBCU95U | 469.3674]						
	PROJECT ID	ACCESSION ID	ORGANISMS	CLASS	PROTEIN FUNCTION	PROTEIN ID	%IDENTITY
**Matched Family**	17827	CP000863	*Acinetobacter baumannii* ACICU, complete genome.	Gammaproteobacteria	hypothetical protein	ACC57277	100.0

Input Sequence	fig|469.3674.peg.2566 Exodeoxyribonuclease VII large subunit (EC 3.1.11.6) [*Acinetobacter* IRMCBCU95U | 469.3674]						
	PROJECT ID	ACCESSION ID	ORGANISMS	CLASS	PROTEIN FUNCTION	PROTEIN ID	%IDENTITY
**Matched Family**	13001	XXX	Proteus mirabilis HI4320	Gammaproteobacteria	XXX		100.0

Input Sequence	fig|469.3674.peg.2409 Phytochrome-like protein; Cph2 [*Acinetobacter* IRMCBCU95U | 469.3674]						
	PROJECT ID	ACCESSION ID	ORGANISMS	CLASS	PROTEIN FUNCTION	PROTEIN ID	%IDENTITY
**Matched Family**	17477	CP000521	*Acinetobacter baumannii* ATCC 17978, complete genome.	Gammaproteobacteria	GGDEF	ABO11498	100.0

Input Sequence	fig|469.3674.peg.3473 Outer membrane protein E [*Acinetobacter* IRMCBCU95U | 469.3674]						
	PROJECT ID	ACCESSION ID	ORGANISMS	CLASS	PROTEIN FUNCTION	PROTEIN ID	%IDENTITY
**Matched Family**	17827	CP000863	*Acinetobacter baumannii* ACICU, complete genome.	Gammaproteobacteria	Long-chain fatty acid transport protein	ACC57127	100.0

Input Sequence	fig|469.3674.peg.504 Glycerophosphoryl diester phosphodiesterase (EC 3.1.4.46) [*Acinetobacter* IRMCBCU95U | 469.3674]						
	PROJECT ID	ACCESSION ID	ORGANISMS	CLASS	PROTEIN FUNCTION	PROTEIN ID	%IDENTITY

**Matched Family**	17827	CP000863	*Acinetobacter baumannii* ACICU, complete genome.	Gammaproteobacteria	uncharacterized protein conserved in bacteria	ACC55514	100.0

**Input Sequence**	fig|469.3674.peg.1089 Uncharacterized MFS-type transporter [*Acinetobacter* IRMCBCU95U | 469.3674]						
	PROJECT ID	ACCESSION ID	ORGANISMS	CLASS	PROTEIN FUNCTION	PROTEIN ID	%IDENTITY
**Matched Family**	17827	CP000863	*Acinetobacter baumannii* ACICU, complete genome.	Gammaproteobacteria	Permease of the major facilitator superfamily	ACC55911	100.0

**Input Sequence**	fig|469.3674.peg.1388 Membrane protein mosC [*Acinetobacter* IRMCBCU95U | 469.3674]						
	PROJECT ID	ACCESSION ID	ORGANISMS	CLASS	PROTEIN FUNCTION	PROTEIN ID	%IDENTITY
**Matched Family**	13001	XXX	Proteus mirabilis HI4320	Gammaproteobacteria	XXX		100.0

**Input Sequence**	fig|469.3674.peg.5945 Inner membrane protein YihY, formerly thought to be RNase BN [*Acinetobacter* IRMCBCU95U | 469.3674]						
	PROJECT ID	ACCESSION ID	ORGANISMS	CLASS	PROTEIN FUNCTION	PROTEIN ID	%IDENTITY
**Matched Family**	13001	XXX	Proteus mirabilis HI4320	Gammaproteobacteria	XXX		100.0

**Input Sequence**	fig|469.3674.peg.5461 AmpG permease [*Acinetobacter* IRMCBCU95U | 469.3674]

	PROJECT ID	ACCESSION ID	ORGANISMS	CLASS	PROTEIN FUNCTION	PROTEIN ID	%IDENTITY
**Matched Family**	17827	CP000863	*Acinetobacter baumannii* ACICU, complete genome.	Gammaproteobacteria	Permease of the major facilitator superfamily	ACC58318	100.0

**Input Sequence**	fig|469.3674.peg.3306 Phytochrome-like protein; Cph2 [*Acinetobacter* IRMCBCU95U | 469.3674]						
	PROJECT ID	ACCESSION ID	ORGANISMS	CLASS	PROTEIN FUNCTION	PROTEIN ID	%IDENTITY
**Matched Family**	17477	CP000521	*Acinetobacter baumannii* ATCC 17978, complete genome.	Gammaproteobacteria	putative two-component response regulator	ABO12122	100.0

**Input Sequence**	fig|469.3674.peg.4597 Lysophospholipase (EC 3.1.1.5) [*Acinetobacter* IRMCBCU95U | 469.3674]						
	PROJECT ID	ACCESSION ID	ORGANISMS	CLASS	PROTEIN FUNCTION	PROTEIN ID	%IDENTITY
**Matched Family**	17827	CP000863	*Acinetobacter baumannii* ACICU, complete genome.	Gammaproteobacteria	hypothetical protein	ACC57821	100.0

**Input Sequence**	fig|469.3674.peg.6904 NAD(FAD)-utilizing dehydrogenases [*Acinetobacter* IRMCBCU95U | 469.3674]						
	PROJECT ID	ACCESSION ID	ORGANISMS	CLASS	PROTEIN FUNCTION	PROTEIN ID	%IDENTITY
**Matched Family**	17827	CP000863	*Acinetobacter baumannii* ACICU, complete genome.	Gammaproteobacteria	predicted flavoprotein	ACC55708	100.0

Input Sequence	fig|469.3674.peg.7111 FIG00350354: hypothetical protein [*Acinetobacter* IRMCBCU95U | 469.3674]						
	PROJECT ID	ACCESSION ID	ORGANISMS	CLASS	PROTEIN FUNCTION	PROTEIN ID	%IDENTITY
**Matched Family**	17827	CP000863	*Acinetobacter baumannii* ACICU, complete genome.	Gammaproteobacteria	hypothetical protein	ACC56232	100.0

Input Sequence	fig|469.3674.peg.2846 hypothetical protein [*Acinetobacter* IRMCBCU95U | 469.3674]						
	PROJECT ID	ACCESSION ID	ORGANISMS	CLASS	PROTEIN FUNCTION	PROTEIN ID	%IDENTITY
**Matched Family**	17477	CP000521	*Acinetobacter baumannii* ATCC 17978, complete genome.	Gammaproteobacteria	hypothetical protein	ABS90078	100.0

Input Sequence	fig|469.3674.peg.1980 hypothetical protein [*Acinetobacter* IRMCBCU95U | 469.3674]						
	PROJECT ID	ACCESSION ID	ORGANISMS	CLASS	PROTEIN FUNCTION	PROTEIN ID	%IDENTITY
**Matched Family**	17827	CP000863	*Acinetobacter baumannii* ACICU, complete genome.	Gammaproteobacteria	hypothetical protein	ACC55599	100.0

Input Sequence	fig|469.3674.peg.1446 FIG00350547: hypothetical protein [*Acinetobacter* IRMCBCU95U | 469.3674]

	PROJECT ID	ACCESSION ID	ORGANISMS	CLASS	PROTEIN FUNCTION	PROTEIN ID	%IDENTITY
**Matched Family**	17827	CP000863	*Acinetobacter baumannii* ACICU, complete genome.	Gammaproteobacteria	hypothetical protein	ACC56112	100.0

**Input Sequence**	fig|469.3674.peg.4186 hypothetical protein [*Acinetobacter* IRMCBCU95U | 469.3674]						
	PROJECT ID	ACCESSION ID	ORGANISMS	CLASS	PROTEIN FUNCTION	PROTEIN ID	%IDENTITY
**Matched Family**	17827	CP000863	*Acinetobacter baumannii* ACICU, complete genome.	Gammaproteobacteria	hypothetical protein	ACC57602	100.0

**Input Sequence**	fig|469.3674.peg.1048 Uncharacterized MFS-type transporter [*Acinetobacter* IRMCBCU95U | 469.3674]						
	PROJECT ID	ACCESSION ID	ORGANISMS	CLASS	PROTEIN FUNCTION	PROTEIN ID	%IDENTITY
**Matched Family**	17827	CP000863	*Acinetobacter baumannii* ACICU, complete genome.	Gammaproteobacteria	Permease of the major facilitator superfamily	ACC55880	100.0

**Input Sequence**	fig|469.3674.peg.5035 Uncharacterized MFS-type transporter [*Acinetobacter* IRMCBCU95U | 469.3674]						
	PROJECT ID	ACCESSION ID	ORGANISMS	CLASS	PROTEIN FUNCTION	PROTEIN ID	%IDENTITY
**Matched Family**	17827	CP000863	*Acinetobacter baumannii* ACICU, complete genome.	Gammaproteobacteria	Permease of the major facilitator superfamily	ACC57995	100.0

**Input Sequence**	fig|469.3674.peg.3525 FIG00350868: hypothetical protein [*Acinetobacter* IRMCBCU95U | 469.3674]						
	PROJECT ID	ACCESSION ID	ORGANISMS	CLASS	PROTEIN FUNCTION	PROTEIN ID	%IDENTITY
**Matched Family**	17827	CP000863	*Acinetobacter baumannii* ACICU, complete genome.	Gammaproteobacteria	hypothetical protein	ACC57156	100.0

**Input Sequence**	fig|469.3674.peg.4517 ATP-dependent RNA helicase RhlB (EC 3.6.4.13) [*Acinetobacter* IRMCBCU95U | 469.3674]						
	PROJECT ID	ACCESSION ID	ORGANISMS	CLASS	PROTEIN FUNCTION	PROTEIN ID	%IDENTITY
**Matched Family**	17477	CP000521	*Acinetobacter baumannii* ATCC 17978, complete genome.	Gammaproteobacteria	ATP-dependent RNA helicase (DEAD box)	ABO12683	100.0

**Input Sequence**	fig|469.3674.peg.4457 Polyhydroxyalkanoic acid synthase [*Acinetobacter* IRMCBCU95U | 469.3674]						
	PROJECT ID	ACCESSION ID	ORGANISMS	CLASS	PROTEIN FUNCTION	PROTEIN ID	%IDENTITY
**Matched Family**	17827	CP000863	*Acinetobacter baumannii* ACICU, complete genome.	Gammaproteobacteria	Poly(3-hydroxyalkanoate) synthetase	ACC57738	100.0

**Input Sequence**	fig|469.3674.peg.962 Outer membrane beta-barrel assembly protein BamB [*Acinetobacter* IRMCBCU95U | 469.3674]						
	PROJECT	ACCESSION	ORGANISMS	CLASS	PROTEIN FUNCTION	PROTEIN	%IDENTITY

	PROJECT ID	ACCESSION ID	ORGANISMS	CLASS	PROTEIN FUNCTION	PROTEIN ID	%IDENTITY
**Matched Family**	17477	CP000521	*Acinetobacter baumannii* ATCC 17978, complete genome.	Gammaproteobacteria	hypothetical protein	ABO10958	100.0

**Input Sequence**	fig|469.3674.peg.1459 FIG00350262: hypothetical protein [*Acinetobacter* IRMCBCU95U | 469.3674]						
	PROJECT ID	ACCESSION ID	ORGANISMS	CLASS	PROTEIN FUNCTION	PROTEIN ID	%IDENTITY
**Matched Family**	28921	XXX	*Acinetobacter baumannii* AYE	Gammaproteobacteria	XXX		100.0

**Input Sequence**	fig|469.3674.peg.5220 Efflux ABC transporter, permease protein [*Acinetobacter* IRMCBCU95U | 469.3674]						
	PROJECT ID	ACCESSION ID	ORGANISMS	CLASS	PROTEIN FUNCTION	PROTEIN ID	%IDENTITY
**Matched Family**	13001	XXX	Proteus mirabilis HI4320	Gammaproteobacteria	XXX		100.0

**Input Sequence**	fig|469.3674.peg.7298 hypothetical protein [*Acinetobacter* IRMCBCU95U | 469.3674]						
	PROJECT ID	ACCESSION ID	ORGANISMS	CLASS	PROTEIN FUNCTION	PROTEIN ID	%IDENTITY
**Matched Family**	17827	CP000863	*Acinetobacter baumannii* ACICU, complete genome.	Gammaproteobacteria	hypothetical protein	ACC56315	100.0

**Input Sequence**	fig|469.3674.peg.6771 Mll9366 protein [*Acinetobacter* IRMCBCU95U | 469.3674]						
	PROJECT ID	ACCESSION ID	ORGANISMS	CLASS	PROTEIN FUNCTION	PROTEIN ID	%IDENTITY
**Matched Family**	17477	CP000521	*Acinetobacter baumannii* ATCC 17978, complete genome.	Gammaproteobacteria	hypothetical protein	ABO10688	100.0

**Input Sequence**	fig|469.3674.peg.7260 putative lipoprotein [*Acinetobacter* IRMCBCU95U | 469.3674]						
	PROJECT ID	ACCESSION ID	ORGANISMS	CLASS	PROTEIN FUNCTION	PROTEIN ID	%IDENTITY
**Matched Family**	17827	CP000863	*Acinetobacter baumannii* ACICU, complete genome.	Gammaproteobacteria	Heat shock protein	ACC56301	100.0

**Input Sequence**	fig|469.3674.peg.5526 NAD+--asparagine ADP-ribosyltransferase [*Acinetobacter* IRMCBCU95U | 469.3674]						
	PROJECT ID	ACCESSION ID	ORGANISMS	CLASS	PROTEIN FUNCTION	PROTEIN ID	%IDENTITY
**Matched Family**	21111	CP001182	*Acinetobacter baumannii* AB0057, complete genome.	Gammaproteobacteria	phage putative head morphogenesis protein	ACJ42053	100.0

**Input Sequence**	fig|469.3674.peg.2759 T6SS component TssA (ImpA) [*Acinetobacter* IRMCBCU95U | 469.3674]						
	PROJECT ID	ACCESSION ID	ORGANISMS	CLASS	PROTEIN FUNCTION	PROTEIN ID	%IDENTITY

	PROJECT ID	ACCESSION ID	ORGANISMS	CLASS	PROTEIN FUNCTION	PROTEIN ID	%IDENTITY
**Matched Family**	17477	CP000521	*Acinetobacter baumannii* ATCC 17978, complete genome.	Gammaproteobacteria	hypothetical protein	ABO11736	100.0

**Input Sequence**	fig|469.3674.peg.3833 Hydrolase, alpha/beta fold family functionally coupled to Phosphoribulokinase [*Acinetobacter* IRMCBCU95U | 469.3674]						
	PROJECT ID	ACCESSION ID	ORGANISMS	CLASS	PROTEIN FUNCTION	PROTEIN ID	%IDENTITY
**Matched Family**	17827	CP000863	*Acinetobacter baumannii* ACICU, complete genome.	Gammaproteobacteria	predicted hydrolase of the alpha/beta-hydrolase fold	ACC57324	100.0

**Input Sequence**	fig|469.3674.peg.4083 Nucleoside:H+ symporter:Major facilitator superfamily [*Acinetobacter* IRMCBCU95U | 469.3674]						
	PROJECT ID	ACCESSION ID	ORGANISMS	CLASS	PROTEIN FUNCTION	PROTEIN ID	%IDENTITY
**Matched Family**	17827	CP000863	*Acinetobacter baumannii* ACICU, complete genome.	Gammaproteobacteria	Permease of the major facilitator superfamily	ACC57451	100.0

**Input Sequence**	fig|469.3674.peg.2634 diguanylate cyclase/phosphodiesterase (GGDEF & EAL domains) with PAS/PAC sensor(s) [*Acinetobacter* IRMCBCU95U | 469.3674]						
	PROJECT ID	ACCESSION ID	ORGANISMS	CLASS	PROTEIN FUNCTION	PROTEIN ID	%IDENTITY
**Matched Family**	17827	CP000863	*Acinetobacter baumannii* ACICU, complete genome.	Gammaproteobacteria	EAL domain protein	ACC56560	100.0

**Input Sequence**	fig|469.3674.peg.6035 Magnesium and cobalt transport protein CorA [*Acinetobacter* IRMCBCU95U | 469.3674]						
	PROJECT ID	ACCESSION ID	ORGANISMS	CLASS	PROTEIN FUNCTION	PROTEIN ID	%IDENTITY
**Matched Family**	28921	XXX	*Acinetobacter baumannii* AYE	Gammaproteobacteria	XXX		100.0

**Input Sequence**	fig|469.3674.peg.3497 Osmosensitive K+ channel histidine kinase KdpD [*Acinetobacter* IRMCBCU95U | 469.3674]						
	PROJECT ID	ACCESSION ID	ORGANISMS	CLASS	PROTEIN FUNCTION	PROTEIN ID	%IDENTITY
**Matched Family**	17827	CP000863	*Acinetobacter baumannii* ACICU, complete genome.	Gammaproteobacteria	Signal transduction histidine kinase	ACC57139	100.0

**Input Sequence**	fig|469.3674.peg.5711 Outer membrane protein A precursor [*Acinetobacter* IRMCBCU95U | 469.3674]						
	PROJECT ID	ACCESSION ID	ORGANISMS	CLASS	PROTEIN FUNCTION	PROTEIN ID	%IDENTITY
**Matched Family**	13001	XXX	Proteus mirabilis HI4320	Gammaproteobacteria	XXX		100.0

**Input Sequence**	fig|469.3674.peg.6519 Nicotinate phosphoribosyltransferase (EC 6.3.4.21) [*Acinetobacter* IRMCBCU95U | 469.3674]						
	PROJECT ID	ACCESSION ID	ORGANISMS	CLASS	PROTEIN FUNCTION	PROTEIN ID	%IDENTITY
**Matched Family**	30993	CP001172	*Acinetobacter baumannii* AB307-0294, complete	Gammaproteobacteria	nicotinate phosphoribosyltransferase	ACJ56759	100.0

			genome.				

Input Sequence	fig|469.3674.peg.5126 Murein endolytic transglycosylase MltG [*Acinetobacter* IRMCBCU95U | 469.3674]						
	PROJECT ID	ACCESSION ID	ORGANISMS	CLASS	PROTEIN FUNCTION	PROTEIN ID	%IDENTITY
**Matched Family**	17477	CP000521	*Acinetobacter baumannii* ATCC 17978, complete genome.	Gammaproteobacteria	hypothetical protein	ABO12946	100.0

Input Sequence	fig|469.3674.peg.5761 hypothetical protein [*Acinetobacter* IRMCBCU95U | 469.3674]						
	PROJECT ID	ACCESSION ID	ORGANISMS	CLASS	PROTEIN FUNCTION	PROTEIN ID	%IDENTITY
**Matched Family**	21111	CP001182	*Acinetobacter baumannii* AB0057, complete genome.	Gammaproteobacteria	conserved hypothetical protein	ACJ41942	100.0

Input Sequence	fig|469.3674.peg.5617 DUF1176 domain-containing protein [*Acinetobacter* IRMCBCU95U | 469.3674]						
	PROJECT ID	ACCESSION ID	ORGANISMS	CLASS	PROTEIN FUNCTION	PROTEIN ID	%IDENTITY
**Matched Family**	17827	CP000863	*Acinetobacter baumannii* ACICU, complete genome.	Gammaproteobacteria	hypothetical protein	ACC58351	100.0

**Input**	fig|469.3674.peg.101 FIG00353483: hypothetical protein [*Acinetobacter* IRMCBCU95U | 469.3674]

Sequence							
	PROJECT ID	ACCESSION ID	ORGANISMS	CLASS	PROTEIN FUNCTION	PROTEIN ID	%IDENTITY
**Matched Family**	17827	CP000863	*Acinetobacter baumannii* ACICU, complete genome.	Gammaproteobacteria	hypothetical protein	ACC57512	100.0

**Input Sequence**	fig|469.3674.peg.3500 Transcriptional regulator, AraC family [*Acinetobacter* IRMCBCU95U | 469.3674]						
	PROJECT ID	ACCESSION ID	ORGANISMS	CLASS	PROTEIN FUNCTION	PROTEIN ID	%IDENTITY
**Matched Family**	17827	CP000863	*Acinetobacter baumannii* ACICU, complete genome.	Gammaproteobacteria	AraC-type DNA-binding domain-containing protein	ACC57141	100.0

**Input Sequence**	fig|469.3674.peg.5811 Poly(glycerol-phosphate) alpha-glucosyltransferase (EC 2.4.1.52) [*Acinetobacter* IRMCBCU95U | 469.3674]						
	PROJECT ID	ACCESSION ID	ORGANISMS	CLASS	PROTEIN FUNCTION	PROTEIN ID	%IDENTITY
**Matched Family**	17827	CP000863	*Acinetobacter baumannii* ACICU, complete genome.	Gammaproteobacteria	Glycosyltransferase	ACC58462	100.0

**Input Sequence**	fig|469.3674.peg.5586 Transcriptional regulator, AraC family [*Acinetobacter* IRMCBCU95U | 469.3674]						
	PROJECT ID	ACCESSION ID	ORGANISMS	CLASS	PROTEIN FUNCTION	PROTEIN ID	%IDENTITY
**Matched Family**	17827	CP000863	*Acinetobacter baumannii* ACICU,	Gammaproteobacteria	AraC-type DNA-binding domain-containing protein	ACC58333	100.0

			complete genome.				

Input Sequence	fig|469.3674.peg.5830 hypothetical protein [*Acinetobacter* IRMCBCU95U | 469.3674]						
	PROJECT ID	ACCESSION ID	ORGANISMS	CLASS	PROTEIN FUNCTION	PROTEIN ID	%IDENTITY
Matched Family	17827	CP000863	*Acinetobacter baumannii* ACICU, complete genome.	Gammaproteobacteria	hypothetical protein	ACC58474	100.0

Input Sequence	fig|469.3674.peg.226 Iron siderophore sensor protein [*Acinetobacter* IRMCBCU95U | 469.3674]						
	PROJECT ID	ACCESSION ID	ORGANISMS	CLASS	PROTEIN FUNCTION	PROTEIN ID	%IDENTITY
Matched Family	21111	CP001182	*Acinetobacter baumannii* AB0057, complete genome.	Gammaproteobacteria	putative transmembrane sensor protein FecR	ACJ40778	100.0

Input Sequence	fig|469.3674.peg.6025 AdeT [*Acinetobacter* IRMCBCU95U | 469.3674]						
	PROJECT ID	ACCESSION ID	ORGANISMS	CLASS	PROTEIN FUNCTION	PROTEIN ID	%IDENTITY
Matched Family	13001	XXX	Proteus mirabilis HI4320	Gammaproteobacteria	XXX		100.0

Input Sequence	fig|469.3674.peg.6911 hypothetical protein [*Acinetobacter* IRMCBCU95U | 469.3674]						
	PROJECT	ACCESSION	ORGANISMS	CLASS	PROTEIN FUNCTION	PROTEIN	%IDENTITY

	ID	ID				ID	
**Matched Family**	17827	CP000863	*Acinetobacter baumannii* ACICU, complete genome.	Gammaproteobacteria	uncharacterized protein conserved in bacteria	ACC55703	100.0

**Input Sequence**	fig|469.3674.peg.5772 hypothetical protein [*Acinetobacter* IRMCBCU95U | 469.3674]						
	PROJECT ID	ACCESSION ID	ORGANISMS	CLASS	PROTEIN FUNCTION	PROTEIN ID	%IDENTITY
**Matched Family**	17827	CP000863	*Acinetobacter baumannii* ACICU, complete genome.	Gammaproteobacteria	hypothetical protein	ACC58432	100.0

**Input Sequence**	fig|469.3674.peg.4795 Xanthine and CO dehydrogenases maturation factor, XdhC/CoxF family [*Acinetobacter* IRMCBCU95U | 469.3674]						
	PROJECT ID	ACCESSION ID	ORGANISMS	CLASS	PROTEIN FUNCTION	PROTEIN ID	%IDENTITY
**Matched Family**	17827	CP000863	*Acinetobacter baumannii* ACICU, complete genome.	Gammaproteobacteria	Xanthine and CO dehydrogenase maturation factor, XdhC/CoxF family	ACC57871	100.0

**Input Sequence**	fig|469.3674.peg.3056 DNA polymerase III delta prime subunit (EC 2.7.7.7) [*Acinetobacter* IRMCBCU95U | 469.3674]						
	PROJECT ID	ACCESSION ID	ORGANISMS	CLASS	PROTEIN FUNCTION	PROTEIN ID	%IDENTITY
**Matched Family**	17827	CP000863	*Acinetobacter baumannii* ACICU, complete genome.	Gammaproteobacteria	ATPase	ACC56917	100.0

**Input Sequence**	fig|469.3674.peg.5422 Nonaprenyl diphosphate synthase (geranyl-diphosphate specific) (EC 2.5.1.84) [*Acinetobacter* IRMCBCU95U | 469.3674]					
	PROJECT ID	ACCESSION ID	ORGANISMS	CLASS	PROTEIN FUNCTION	PROTEIN ID	%IDENTITY
**Matched Family**	13001	XXX	Proteus mirabilis HI4320	Gammaproteobacteria	XXX		100.0

**Input Sequence**	fig|469.3674.peg.4195 Lysophospholipase (EC 3.1.1.5) [*Acinetobacter* IRMCBCU95U | 469.3674]					
	PROJECT ID	ACCESSION ID	ORGANISMS	CLASS	PROTEIN FUNCTION	PROTEIN ID	%IDENTITY
**Matched Family**	17827	CP000863	*Acinetobacter baumannii* ACICU, complete genome.	Gammaproteobacteria	Lysophospholipase	ACC57608	100.0

**Input Sequence**	fig|469.3674.peg.1139 Serine phosphatase RsbU, regulator of sigma subunit [*Acinetobacter* IRMCBCU95U | 469.3674]					
	PROJECT ID	ACCESSION ID	ORGANISMS	CLASS	PROTEIN FUNCTION	PROTEIN ID	%IDENTITY
**Matched Family**	13001	XXX	Proteus mirabilis HI4320	Gammaproteobacteria	XXX		100.0

**Input Sequence**	fig|469.3674.peg.3524 SurE-like protein [*Acinetobacter* IRMCBCU95U | 469.3674]					
	PROJECT ID	ACCESSION ID	ORGANISMS	CLASS	PROTEIN FUNCTION	PROTEIN ID	%IDENTITY
**Matched Family**	17827	CP000863	*Acinetobacter baumannii* ACICU, complete genome.	Gammaproteobacteria	predicted acid phosphatase	ACC57155	100.0

Input Sequence	fig|469.3674.peg.3183 Transcriptional regulator, AraC family [*Acinetobacter* IRMCBCU95U | 469.3674]						
	PROJECT ID	ACCESSION ID	ORGANISMS	CLASS	PROTEIN FUNCTION	PROTEIN ID	%IDENTITY
Matched Family	17827	CP000863	*Acinetobacter baumannii* ACICU, complete genome.	Gammaproteobacteria	AraC-type DNA-binding domain-containing protein	ACC56977	100.0

Input Sequence	fig|469.3674.peg.1365 hypothetical protein [*Acinetobacter* IRMCBCU95U | 469.3674]						
	PROJECT ID	ACCESSION ID	ORGANISMS	CLASS	PROTEIN FUNCTION	PROTEIN ID	%IDENTITY
Matched Family	17827	CP000863	*Acinetobacter baumannii* ACICU, complete genome.	Gammaproteobacteria	hypothetical protein	ACC56060	100.0

Input Sequence	fig|469.3674.peg.2959 Replication protein [*Acinetobacter* IRMCBCU95U | 469.3674]						
	PROJECT ID	ACCESSION ID	ORGANISMS	CLASS	PROTEIN FUNCTION	PROTEIN ID	%IDENTITY
Matched Family	17477	CP000523	*Acinetobacter baumannii* ATCC 17978 plasmid pAB2, complete sequence.	Gammaproteobacteria	DNA replication protein	ABO13861	100.0

Input Sequence	fig|469.3674.peg.6019 hypothetical protein [*Acinetobacter* IRMCBCU95U | 469.3674]

	PROJECT ID	ACCESSION ID	ORGANISMS	CLASS	PROTEIN FUNCTION	PROTEIN ID	%IDENTITY
**Matched Family**	17827	CP000863	*Acinetobacter baumannii* ACICU, complete genome.	Gammaproteobacteria	predicted metal-dependent hydrolase	ACC58598	100.0

**Input Sequence**	fig|469.3674.peg.6214 hypothetical protein [*Acinetobacter* IRMCBCU95U | 469.3674]						
	PROJECT ID	ACCESSION ID	ORGANISMS	CLASS	PROTEIN FUNCTION	PROTEIN ID	%IDENTITY
**Matched Family**	21111	CP001182	*Acinetobacter baumannii* AB0057, complete genome.	Gammaproteobacteria	hypothetical protein	ACJ43024	100.0

**Input Sequence**	fig|469.3674.peg.5211 hypothetical protein [*Acinetobacter* IRMCBCU95U | 469.3674]						
	PROJECT ID	ACCESSION ID	ORGANISMS	CLASS	PROTEIN FUNCTION	PROTEIN ID	%IDENTITY
**Matched Family**	17827	CP000863	*Acinetobacter baumannii* ACICU, complete genome.	Gammaproteobacteria	hypothetical protein	ACC58171	100.0

**Input Sequence**	fig|469.3674.peg.102 FIG00353596: hypothetical protein [*Acinetobacter* IRMCBCU95U | 469.3674]						
	PROJECT ID	ACCESSION ID	ORGANISMS	CLASS	PROTEIN FUNCTION	PROTEIN ID	%IDENTITY
**Matched Family**	17827	CP000863	*Acinetobacter baumannii* ACICU, complete genome.	Gammaproteobacteria	hypothetical protein	ACC57511	100.0

**Input Sequence**	fig|469.3674.peg.2867 Nitrilotriacetate monooxygenase component B (EC 1.14.13.-) [*Acinetobacter* IRMCBCU95U | 469.3674]						
	PROJECT ID	ACCESSION ID	ORGANISMS	CLASS	PROTEIN FUNCTION	PROTEIN ID	%IDENTITY
**Matched Family**	17827	CP000863	*Acinetobacter baumannii* ACICU, complete genome.	Gammaproteobacteria	conserved protein/domain typically associated with flavoprotein oxygenases, DIM6/NTAB family	ACC56674	100.0

**Input Sequence**	fig|469.3674.peg.1051 Drug/metabolite exporter [*Acinetobacter* IRMCBCU95U | 469.3674]						
	PROJECT ID	ACCESSION ID	ORGANISMS	CLASS	PROTEIN FUNCTION	PROTEIN ID	%IDENTITY
**Matched Family**	17827	CP000863	*Acinetobacter baumannii* ACICU, complete genome.	Gammaproteobacteria	Permease of the drug/metabolite transporter (DMT) superfamily	ACC55882	100.0

**Input Sequence**	fig|469.3674.peg.5235 Site-specific tyrosine recombinase XerC [*Acinetobacter* IRMCBCU95U | 469.3674]						
	PROJECT ID	ACCESSION ID	ORGANISMS	CLASS	PROTEIN FUNCTION	PROTEIN ID	%IDENTITY
**Matched Family**	13001	XXX	Proteus mirabilis HI4320	Gammaproteobacteria	XXX		100.0

**Input Sequence**	fig|469.3674.peg.5688 Auxin efflux carrier family protein [*Acinetobacter* IRMCBCU95U | 469.3674]						
	PROJECT ID	ACCESSION ID	ORGANISMS	CLASS	PROTEIN FUNCTION	PROTEIN ID	%IDENTITY

Matched Family	[17827](#)	[CP000863](#)	*Acinetobacter baumannii* ACICU, complete genome.	Gammaproteobacteria	predicted permease	[ACC58387](#)	100.0

Input Sequence	fig|469.3674.peg.3776 Transcriptional regulator, LysR family [*Acinetobacter* IRMCBCU95U | 469.3674]						
	PROJECT ID	ACCESSION ID	ORGANISMS	CLASS	PROTEIN FUNCTION	PROTEIN ID	%IDENTITY
Matched Family	17477	CP000521	*Acinetobacter baumannii* ATCC 17978, complete genome.	Gammaproteobacteria	putative transcriptional regulator (LysR family)	ABO12301	100.0

Input Sequence	fig|469.3674.peg.7012 Permease of the drug/metabolite transporter (DMT) superfamily [*Acinetobacter* IRMCBCU95U | 469.3674]						
	PROJECT ID	ACCESSION ID	ORGANISMS	CLASS	PROTEIN FUNCTION	PROTEIN ID	%IDENTITY
Matched Family	17477	CP000521	*Acinetobacter baumannii* ATCC 17978, complete genome.	Gammaproteobacteria	putative membrane protein	ABO10789	100.0

Input Sequence	fig|469.3674.peg.3092 FIG00350684: hypothetical protein [*Acinetobacter* IRMCBCU95U | 469.3674]						
	PROJECT ID	ACCESSION ID	ORGANISMS	CLASS	PROTEIN FUNCTION	PROTEIN ID	%IDENTITY
Matched Family	17827	CP000863	*Acinetobacter baumannii* ACICU, complete genome.	Gammaproteobacteria	predicted membrane protein	ACC56935	100.0

**Input Sequence**	fig|469.3674.peg.5209 Permease of the drug/metabolite transporter (DMT) superfamily [*Acinetobacter* IRMCBCU95U | 469.3674]						
	PROJECT ID	ACCESSION ID	ORGANISMS	CLASS	PROTEIN FUNCTION	PROTEIN ID	%IDENTITY
**Matched Family**	17477	CP000521	*Acinetobacter baumannii* ATCC 17978, complete genome.	Gammaproteobacteria	putative membrane protein	ABO13031	100.0

**Input Sequence**	fig|469.3674.peg.4229 Pseudouridine synthase (EC 4.2.1.70), PA2043 type [*Acinetobacter* IRMCBCU95U | 469.3674]						
	PROJECT ID	ACCESSION ID	ORGANISMS	CLASS	PROTEIN FUNCTION	PROTEIN ID	%IDENTITY
**Matched Family**	17477	CP000521	*Acinetobacter baumannii* ATCC 17978, complete genome.	Gammaproteobacteria	putative pseudouridylate synthase	ABO12544	100.0

**Input Sequence**	fig|469.3674.peg.2791 Permease of the drug/metabolite transporter (DMT) superfamily [*Acinetobacter* IRMCBCU95U | 469.3674]						
	PROJECT ID	ACCESSION ID	ORGANISMS	CLASS	PROTEIN FUNCTION	PROTEIN ID	%IDENTITY
**Matched Family**	17477	CP000521	*Acinetobacter baumannii* ATCC 17978, complete genome.	Gammaproteobacteria	hypothetical protein	ABO11751	100.0

**Input**	fig|469.3674.peg.4873 Permease of the drug/metabolite transporter (DMT) superfamily [*Acinetobacter* IRMCBCU95U | 469.3674]

Sequence							
	PROJECT ID	ACCESSION ID	ORGANISMS	CLASS	PROTEIN FUNCTION	PROTEIN ID	%IDENTITY
**Matched Family**	17827	CP000863	*Acinetobacter baumannii* ACICU, complete genome.	Gammaproteobacteria	Permease of the drug/metabolite transporter (DMT) superfamily	ACC57915	100.0

**Input Sequence**	fig|469.3674.peg.6662 hypothetical protein [*Acinetobacter* IRMCBCU95U | 469.3674]						
	PROJECT ID	ACCESSION ID	ORGANISMS	CLASS	PROTEIN FUNCTION	PROTEIN ID	%IDENTITY
**Matched Family**	17827	CP000863	*Acinetobacter baumannii* ACICU, complete genome.	Gammaproteobacteria	hypothetical protein	ACC55341	100.0

**Input Sequence**	fig|469.3674.peg.6357 33-36 kDa outer membrane protein [*Acinetobacter* IRMCBCU95U | 469.3674]						
	PROJECT ID	ACCESSION ID	ORGANISMS	CLASS	PROTEIN FUNCTION	PROTEIN ID	%IDENTITY
**Matched Family**	17827	CP000863	*Acinetobacter baumannii* ACICU, complete genome.	Gammaproteobacteria	34 kDa outer membrane protein	ACC58802	100.0

**Input Sequence**	fig|469.3674.peg.4559 Mutator MutT protein (7,8-dihydro-8-oxoguanine-triphosphatase) (EC 3.6.1.-) / Thiazole tautomerase TenI-like domain [*Acinetobacter* IRMCBCU95U | 469.3674]						
	PROJECT ID	ACCESSION ID	ORGANISMS	CLASS	PROTEIN FUNCTION	PROTEIN ID	%IDENTITY
**Matched Family**	17827	CP000863	*Acinetobacter baumannii* ACICU,	Gammaproteobacteria	Thiamine monophosphate synthase	ACC57796	100.0

			complete genome.				

Input Sequence	fig|469.3674.peg.1477 FIG00350000: hypothetical protein [*Acinetobacter* IRMCBCU95U | 469.3674]						
	PROJECT ID	ACCESSION ID	ORGANISMS	CLASS	PROTEIN FUNCTION	PROTEIN ID	%IDENTITY
**Matched Family**	28921	XXX	*Acinetobacter baumannii* AYE	Gammaproteobacteria	XXX		100.0

Input Sequence	fig|469.3674.peg.6882 Transcriptional regulator, LysR family [*Acinetobacter* IRMCBCU95U | 469.3674]						
	PROJECT ID	ACCESSION ID	ORGANISMS	CLASS	PROTEIN FUNCTION	PROTEIN ID	%IDENTITY
**Matched Family**	17827	CP000863	*Acinetobacter baumannii* ACICU, complete genome.	Gammaproteobacteria	Transcriptional regulator	ACC55720	100.0

Input Sequence	fig|469.3674.peg.140 Phage antirepressor protein [*Acinetobacter* IRMCBCU95U | 469.3674]						
	PROJECT ID	ACCESSION ID	ORGANISMS	CLASS	PROTEIN FUNCTION	PROTEIN ID	%IDENTITY
**Matched Family**	17827	CP000863	*Acinetobacter baumannii* ACICU, complete genome.	Gammaproteobacteria	putative antirepressor protein	ACC57484	100.0

Input Sequence	fig|469.3674.peg.3364 Transcriptional regulator, LysR family [*Acinetobacter* IRMCBCU95U | 469.3674]						
	PROJECT	ACCESSION	ORGANISMS	CLASS	PROTEIN FUNCTION	PROTEIN	%IDENTITY

	PROJECT ID	ACCESSION ID	ORGANISMS	CLASS	PROTEIN FUNCTION	PROTEIN ID	%IDENTITY
**Matched Family**	17827	CP000863	*Acinetobacter baumannii* ACICU, complete genome.	Gammaproteobacteria	Transcriptional regulator	ACC57077	100.0

**Input Sequence**	fig|469.3674.peg.5863 Hydrolase, alpha/beta fold family [*Acinetobacter* IRMCBCU95U | 469.3674]						
	PROJECT ID	ACCESSION ID	ORGANISMS	CLASS	PROTEIN FUNCTION	PROTEIN ID	%IDENTITY
**Matched Family**	17827	CP000863	*Acinetobacter baumannii* ACICU, complete genome.	Gammaproteobacteria	Hydrolase of the alpha/beta superfamily	ACC58494	100.0

**Input Sequence**	fig|469.3674.peg.5579 Transcriptional regulator, AraC family [*Acinetobacter* IRMCBCU95U | 469.3674]						
	PROJECT ID	ACCESSION ID	ORGANISMS	CLASS	PROTEIN FUNCTION	PROTEIN ID	%IDENTITY
**Matched Family**	17827	CP000863	*Acinetobacter baumannii* ACICU, complete genome.	Gammaproteobacteria	AraC-type DNA-binding domain-containing protein	ACC58329	100.0

**Input Sequence**	fig|469.3674.peg.149 FIG00353605: hypothetical protein [*Acinetobacter* IRMCBCU95U | 469.3674]						
	PROJECT ID	ACCESSION ID	ORGANISMS	CLASS	PROTEIN FUNCTION	PROTEIN ID	%IDENTITY
**Matched Family**	17827	CP000863	*Acinetobacter baumannii* ACICU, complete genome.	Gammaproteobacteria	hypothetical protein	ACC57478	100.0

Input Sequence	fig|469.3674.peg.5938 TonB, C-terminal [*Acinetobacter* IRMCBCU95U | 469.3674]						
	PROJECT ID	ACCESSION ID	ORGANISMS	CLASS	PROTEIN FUNCTION	PROTEIN ID	%IDENTITY
**Matched Family**	17827	CP000863	*Acinetobacter baumannii* ACICU, complete genome.	Gammaproteobacteria	Periplasmic protein TonB	ACC58540	100.0

Input Sequence	fig|469.3674.peg.1474 FIG00350000: hypothetical protein [*Acinetobacter* IRMCBCU95U | 469.3674]						
	PROJECT ID	ACCESSION ID	ORGANISMS	CLASS	PROTEIN FUNCTION	PROTEIN ID	%IDENTITY
**Matched Family**	17827	CP000863	*Acinetobacter baumannii* ACICU, complete genome.	Gammaproteobacteria	predicted metal-dependent hydrolase	ACC56131	100.0

Input Sequence	fig|469.3674.peg.901 putative membrane protein [*Acinetobacter* IRMCBCU95U | 469.3674]						
	PROJECT ID	ACCESSION ID	ORGANISMS	CLASS	PROTEIN FUNCTION	PROTEIN ID	%IDENTITY
**Matched Family**	17827	CP000863	*Acinetobacter baumannii* ACICU, complete genome.	Gammaproteobacteria	putative permease	ACC55790	100.0

Input Sequence	fig|469.3674.peg.5771 Predicted metal-dependent hydrolase [*Acinetobacter* IRMCBCU95U | 469.3674]						
	PROJECT ID	ACCESSION ID	ORGANISMS	CLASS	PROTEIN FUNCTION	PROTEIN ID	%IDENTITY
**Matched**	17827	CP000863	*Acinetobacter*	Gammaproteobacteria	predicted metal-dependent hydrolase	ACC58431	100.0

Family			*baumannii* ACICU, complete genome.				

Input Sequence	fig|469.3674.peg.7235 Urease accessory protein UreD [*Acinetobacter* IRMCBCU95U | 469.3674]						
	PROJECT ID	ACCESSION ID	ORGANISMS	CLASS	PROTEIN FUNCTION	PROTEIN ID	%IDENTITY
Matched Family	13001	XXX	Proteus mirabilis HI4320	Gammaproteobacteria	XXX		100.0

Input Sequence	fig|469.3674.peg.6122 Putative DMT superfamily metabolite efflux protein precursor [*Acinetobacter* IRMCBCU95U | 469.3674]						
	PROJECT ID	ACCESSION ID	ORGANISMS	CLASS	PROTEIN FUNCTION	PROTEIN ID	%IDENTITY
Matched Family	17827	CP000863	*Acinetobacter baumannii* ACICU, complete genome.	Gammaproteobacteria	predicted permease, DMT superfamily	ACC58664	100.0

Input Sequence	fig|469.3674.peg.3923 Hydrolase, alpha/beta fold family [*Acinetobacter* IRMCBCU95U | 469.3674]						
	PROJECT ID	ACCESSION ID	ORGANISMS	CLASS	PROTEIN FUNCTION	PROTEIN ID	%IDENTITY
Matched Family	17477	CP000521	*Acinetobacter baumannii* ATCC 17978, complete genome.	Gammaproteobacteria	Alpha/beta hydrolase	ABO12371	100.0

Input	fig|469.3674.peg.7093 putative hydrolase [*Acinetobacter* IRMCBCU95U | 469.3674]

Sequence							
	PROJECT ID	ACCESSION ID	ORGANISMS	CLASS	PROTEIN FUNCTION	PROTEIN ID	%IDENTITY
**Matched Family**	28921	XXX	*Acinetobacter baumannii* AYE	Gammaproteobacteria	XXX		100.0

**Input Sequence**	fig|469.3674.peg.3136 3'(2'),5'-bisphosphate nucleotidase (EC 3.1.3.7) [*Acinetobacter* IRMCBCU95U | 469.3674]						
	PROJECT ID	ACCESSION ID	ORGANISMS	CLASS	PROTEIN FUNCTION	PROTEIN ID	%IDENTITY
**Matched Family**	17827	CP000863	*Acinetobacter baumannii* ACICU, complete genome.	Gammaproteobacteria	3'-Phosphoadenosine 5'-phosphosulfate (PAPS) 3'-phosphatase	ACC56954	100.0

**Input Sequence**	fig|469.3674.peg.5604 Rod shape-determining protein MreC [*Acinetobacter* IRMCBCU95U | 469.3674]						
	PROJECT ID	ACCESSION ID	ORGANISMS	CLASS	PROTEIN FUNCTION	PROTEIN ID	%IDENTITY
**Matched Family**	13001	XXX	Proteus mirabilis HI4320	Gammaproteobacteria	XXX		100.0

**Input Sequence**	fig|469.3674.peg.1156 Type IV fimbrial biogenesis protein PilY1 [*Acinetobacter* IRMCBCU95U | 469.3674]						
	PROJECT ID	ACCESSION ID	ORGANISMS	CLASS	PROTEIN FUNCTION	PROTEIN ID	%IDENTITY
**Matched Family**	17827	CP000863	*Acinetobacter baumannii* ACICU, complete genome.	Gammaproteobacteria	hypothetical protein	ACC55947	100.0

Input Sequence	fig|469.3674.peg.3831 hypothetical protein [*Acinetobacter* IRMCBCU95U | 469.3674]						
	PROJECT ID	ACCESSION ID	ORGANISMS	CLASS	PROTEIN FUNCTION	PROTEIN ID	%IDENTITY
**Matched Family**	17827	CP000863	*Acinetobacter baumannii* ACICU, complete genome.	Gammaproteobacteria	predicted metal-dependent hydrolase	ACC57323	100.0

Input Sequence	fig|469.3674.peg.3504 Putative oxidoreductase [*Acinetobacter* IRMCBCU95U | 469.3674]						
	PROJECT ID	ACCESSION ID	ORGANISMS	CLASS	PROTEIN FUNCTION	PROTEIN ID	%IDENTITY
**Matched Family**	17827	CP000863	*Acinetobacter baumannii* ACICU, complete genome.	Gammaproteobacteria	Dehydrogenase with different specificities	ACC57144	100.0

Input Sequence	fig|469.3674.peg.943 Tetracenomycin polyketide synthesis O-methyltransferase TcmP [*Acinetobacter* IRMCBCU95U | 469.3674]						
	PROJECT ID	ACCESSION ID	ORGANISMS	CLASS	PROTEIN FUNCTION	PROTEIN ID	%IDENTITY
**Matched Family**	17827	CP000863	*Acinetobacter baumannii* ACICU, complete genome.	Gammaproteobacteria	O-Methyltransferase	ACC55813	100.0

Input Sequence	fig|469.3674.peg.2620 Universal stress protein UspA and related nucleotide-binding proteins [*Acinetobacter* IRMCBCU95U | 469.3674]						
	PROJECT ID	ACCESSION ID	ORGANISMS	CLASS	PROTEIN FUNCTION	PROTEIN ID	%IDENTITY

**Matched Family**	17477	CP000521	*Acinetobacter baumannii* ATCC 17978, complete genome.	Gammaproteobacteria	putative universal stress protein	ABO11674	100.0

**Input Sequence**	fig|469.3674.peg.1813 Glutamate/aspartate ABC transporter, permease protein GltJ (TC 3.A.1.3.4) [*Acinetobacter* IRMCBCU95U | 469.3674]						
	PROJECT ID	ACCESSION ID	ORGANISMS	CLASS	PROTEIN FUNCTION	PROTEIN ID	%IDENTITY
**Matched Family**	17477	CP000521	*Acinetobacter baumannii* ATCC 17978, complete genome.	Gammaproteobacteria	glutamate/aspartate transport protein	ABO11919	100.0

**Input Sequence**	fig|469.3674.peg.1979 hypothetical protein [*Acinetobacter* IRMCBCU95U | 469.3674]						
	PROJECT ID	ACCESSION ID	ORGANISMS	CLASS	PROTEIN FUNCTION	PROTEIN ID	%IDENTITY
**Matched Family**	17827	CP000863	*Acinetobacter baumannii* ACICU, complete genome.	Gammaproteobacteria	hypothetical protein	ACC55598	100.0

**Input Sequence**	fig|469.3674.peg.6916 Magnesium and cobalt efflux protein CorC [*Acinetobacter* IRMCBCU95U | 469.3674]						
	PROJECT ID	ACCESSION ID	ORGANISMS	CLASS	PROTEIN FUNCTION	PROTEIN ID	%IDENTITY
**Matched Family**	17477	CP000521	*Acinetobacter baumannii* ATCC 17978, complete genome.	Gammaproteobacteria	magnesium and cobalt efflux protein	ABO10835	100.0

Input Sequence	fig|469.3674.peg.3797 3-dehydroquinate dehydratase I (EC 4.2.1.10) [*Acinetobacter* IRMCBCU95U | 469.3674]					
	PROJECT ID	ACCESSION ID	ORGANISMS	CLASS	PROTEIN FUNCTION	PROTEIN ID	%IDENTITY
**Matched Family**	17477	CP000521	*Acinetobacter baumannii* ATCC 17978, complete genome.	Gammaproteobacteria	catabolic dehydroquinate dehydratase	ABO12310	100.0

Input Sequence	fig|469.3674.peg.6195 23S rRNA (guanine(745)-N(1))-methyltransferase (EC 2.1.1.187) [*Acinetobacter* IRMCBCU95U | 469.3674]						
	PROJECT ID	ACCESSION ID	ORGANISMS	CLASS	PROTEIN FUNCTION	PROTEIN ID	%IDENTITY
**Matched Family**	17477	CP000521	*Acinetobacter baumannii* ATCC 17978, complete genome.	Gammaproteobacteria	23S ribosomal RNA G745 methyltransferase	ABO13591	100.0

Input Sequence	fig|469.3674.peg.5416 Chromosome segregation ATPases [*Acinetobacter* IRMCBCU95U | 469.3674]						
	PROJECT ID	ACCESSION ID	ORGANISMS	CLASS	PROTEIN FUNCTION	PROTEIN ID	%IDENTITY
**Matched Family**	28921	XXX	*Acinetobacter baumannii* AYE	Gammaproteobacteria	XXX		100.0

Input Sequence	fig|469.3674.peg.1137 FIG003879: Uncharacterized subgroup of the nitrilase superfamily [*Acinetobacter* IRMCBCU95U | 469.3674]

	PROJECT ID	ACCESSION ID	ORGANISMS	CLASS	PROTEIN FUNCTION	PROTEIN ID	%IDENTITY
**Matched Family**	17827	CP000863	*Acinetobacter baumannii* ACICU, complete genome.	Gammaproteobacteria	predicted amidohydrolase	ACC55935	100.0

**Input Sequence**	fig|469.3674.peg.3529 hypothetical protein [*Acinetobacter* IRMCBCU95U | 469.3674]						
	PROJECT ID	ACCESSION ID	ORGANISMS	CLASS	PROTEIN FUNCTION	PROTEIN ID	%IDENTITY
**Matched Family**	17477	CP000521	*Acinetobacter baumannii* ATCC 17978, complete genome.	Gammaproteobacteria	hypothetical protein	ABO12198	100.0

**Input Sequence**	fig|469.3674.peg.1129 hypothetical protein [*Acinetobacter* IRMCBCU95U | 469.3674]						
	PROJECT ID	ACCESSION ID	ORGANISMS	CLASS	PROTEIN FUNCTION	PROTEIN ID	%IDENTITY
**Matched Family**	17477	CP000521	*Acinetobacter baumannii* ATCC 17978, complete genome.	Gammaproteobacteria	hypothetical protein	ABO11064	100.0

**Input Sequence**	fig|469.3674.peg.4306 Peptide chain release factor N(5)-glutamine methyltransferase (EC 2.1.1.297) [*Acinetobacter* IRMCBCU95U | 469.3674]						
	PROJECT ID	ACCESSION ID	ORGANISMS	CLASS	PROTEIN FUNCTION	PROTEIN ID	%IDENTITY
**Matched Family**	17827	CP000863	*Acinetobacter baumannii* ACICU,	Gammaproteobacteria	Methylase of polypeptide chain release factor	ACC57665	100.0

		complete genome.				

Input Sequence	fig|469.3674.peg.1037 Putative deoxyribonuclease YjjV [*Acinetobacter* IRMCBCU95U | 469.3674]						
	PROJECT ID	ACCESSION ID	ORGANISMS	CLASS	PROTEIN FUNCTION	PROTEIN ID	%IDENTITY
**Matched Family**	17827	CP000863	*Acinetobacter baumannii* ACICU, complete genome.	Gammaproteobacteria	Mg-dependent DNase	ACC55873	100.0

Input Sequence	fig|469.3674.peg.3624 putative 2-pyrone-4,6-dicarboxylic acid hydrolase [*Acinetobacter* IRMCBCU95U | 469.3674]						
	PROJECT ID	ACCESSION ID	ORGANISMS	CLASS	PROTEIN FUNCTION	PROTEIN ID	%IDENTITY
**Matched Family**	17827	CP000863	*Acinetobacter baumannii* ACICU, complete genome.	Gammaproteobacteria	predicted metal-dependent hydrolase of the TIM-barrel fold	ACC57218	100.0

Input Sequence	fig|469.3674.peg.8 FIG00350997: hypothetical protein [*Acinetobacter* IRMCBCU95U | 469.3674]						
	PROJECT ID	ACCESSION ID	ORGANISMS	CLASS	PROTEIN FUNCTION	PROTEIN ID	%IDENTITY
**Matched Family**	13001	XXX	Proteus mirabilis HI4320	Gammaproteobacteria	XXX		100.0

Input Sequence	fig|469.3674.peg.5584 Phenylacetic acid degradation operon negative regulatory protein paaX [*Acinetobacter* IRMCBCU95U | 469.3674]						
	PROJECT	ACCESSION	ORGANISMS	CLASS	PROTEIN FUNCTION	PROTEIN	%IDENTITY

	PROJECT ID	ACCESSION ID	ORGANISMS	CLASS	PROTEIN FUNCTION	PROTEIN ID	%IDENTITY
**Matched Family**	13001	XXX	Proteus mirabilis HI4320	Gammaproteobacteria	XXX		100.0

**Input Sequence**	fig|469.3674.peg.1059 Hydroxypyruvate isomerase (EC 5.3.1.22) [*Acinetobacter* IRMCBCU95U | 469.3674]						
	PROJECT ID	ACCESSION ID	ORGANISMS	CLASS	PROTEIN FUNCTION	PROTEIN ID	%IDENTITY
**Matched Family**	13001	XXX	Proteus mirabilis HI4320	Gammaproteobacteria	XXX		100.0

**Input Sequence**	fig|469.3674.peg.5223 hypothetical protein [*Acinetobacter* IRMCBCU95U | 469.3674]						
	PROJECT ID	ACCESSION ID	ORGANISMS	CLASS	PROTEIN FUNCTION	PROTEIN ID	%IDENTITY
**Matched Family**	17827	CP000863	*Acinetobacter baumannii* ACICU, complete genome.	Gammaproteobacteria	uncharacterized protein conserved in bacteria	ACC58177	100.0

**Input Sequence**	fig|469.3674.peg.4815 hypothetical protein [*Acinetobacter* IRMCBCU95U | 469.3674]						
	PROJECT ID	ACCESSION ID	ORGANISMS	CLASS	PROTEIN FUNCTION	PROTEIN ID	%IDENTITY
**Matched Family**	13001	XXX	Proteus mirabilis HI4320	Gammaproteobacteria	XXX		100.0

**Input Sequence**	fig|469.3674.peg.6094 SAM-dependent methyltransferase [*Acinetobacter* IRMCBCU95U | 469.3674]

	PROJECT ID	ACCESSION ID	ORGANISMS	CLASS	PROTEIN FUNCTION	PROTEIN ID	%IDENTITY
**Matched Family**	17827	CP000863	*Acinetobacter baumannii* ACICU, complete genome.	Gammaproteobacteria	SAM-dependent methyltransferase	ACC58648	100.0

**Input Sequence**	fig|469.3674.peg.5085 hypothetical protein [*Acinetobacter* IRMCBCU95U | 469.3674]						
	PROJECT ID	ACCESSION ID	ORGANISMS	CLASS	PROTEIN FUNCTION	PROTEIN ID	%IDENTITY
**Matched Family**	17827	CP000863	*Acinetobacter baumannii* ACICU, complete genome.	Gammaproteobacteria	hypothetical protein	ACC58095	100.0

**Input Sequence**	fig|469.3674.peg.956 Cytoskeleton protein RodZ [*Acinetobacter* IRMCBCU95U | 469.3674]						
	PROJECT ID	ACCESSION ID	ORGANISMS	CLASS	PROTEIN FUNCTION	PROTEIN ID	%IDENTITY
**Matched Family**	17827	CP000863	*Acinetobacter baumannii* ACICU, complete genome.	Gammaproteobacteria	hypothetical protein	ACC55822	100.0

**Input Sequence**	fig|469.3674.peg.221 PhaK-like protein [*Acinetobacter* IRMCBCU95U | 469.3674]						
	PROJECT ID	ACCESSION ID	ORGANISMS	CLASS	PROTEIN FUNCTION	PROTEIN ID	%IDENTITY
**Matched Family**	28921	XXX	*Acinetobacter baumannii* AYE	Gammaproteobacteria	XXX		100.0

Input Sequence	fig|469.3674.peg.3211 Atr protein [*Acinetobacter* IRMCBCU95U | 469.3674]						
	PROJECT ID	ACCESSION ID	ORGANISMS	CLASS	PROTEIN FUNCTION	PROTEIN ID	%IDENTITY
Matched Family	21111	CP001182	*Acinetobacter baumannii* AB0057, complete genome.	Gammaproteobacteria	conserved hypothetical protein	ACJ41253	100.0

Input Sequence	fig|469.3674.peg.6398 hypothetical protein [*Acinetobacter* IRMCBCU95U | 469.3674]						
	PROJECT ID	ACCESSION ID	ORGANISMS	CLASS	PROTEIN FUNCTION	PROTEIN ID	%IDENTITY
Matched Family	13001	XXX	Proteus mirabilis HI4320	Gammaproteobacteria	XXX		100.0

Input Sequence	fig|469.3674.peg.1978 Uroporphyrinogen-III synthase (EC 4.2.1.75) [*Acinetobacter* IRMCBCU95U | 469.3674]						
	PROJECT ID	ACCESSION ID	ORGANISMS	CLASS	PROTEIN FUNCTION	PROTEIN ID	%IDENTITY
Matched Family	17827	CP000863	*Acinetobacter baumannii* ACICU, complete genome.	Gammaproteobacteria	Uroporphyrinogen-III synthase	ACC55597	100.0

Input Sequence	fig|469.3674.peg.529 Transcriptional regulator, AraC family [*Acinetobacter* IRMCBCU95U | 469.3674]						
	PROJECT ID	ACCESSION ID	ORGANISMS	CLASS	PROTEIN FUNCTION	PROTEIN ID	%IDENTITY
Matched Family	17827	CP000863	*Acinetobacter baumannii* ACICU,	Gammaproteobacteria	AraC-type DNA-binding domain-containing protein	ACC55495	100.0

			complete genome.				

**Input Sequence**	fig|469.3674.peg.5801 Putative membrane-spanning protein [*Acinetobacter* IRMCBCU95U | 469.3674]						
	PROJECT ID	ACCESSION ID	ORGANISMS	CLASS	PROTEIN FUNCTION	PROTEIN ID	%IDENTITY
**Matched Family**	17827	CP000863	*Acinetobacter baumannii* ACICU, complete genome.	Gammaproteobacteria	hypothetical protein	ACC58455	100.0

**Input Sequence**	fig|469.3674.peg.5808 Beta-1,4-galactosyltransferase [*Acinetobacter* IRMCBCU95U | 469.3674]						
	PROJECT ID	ACCESSION ID	ORGANISMS	CLASS	PROTEIN FUNCTION	PROTEIN ID	%IDENTITY
**Matched Family**	28921	XXX	*Acinetobacter baumannii* AYE	Gammaproteobacteria	XXX		100.0

**Input Sequence**	fig|469.3674.peg.6014 Flavohemoglobin / Nitric oxide dioxygenase (EC 1.14.12.17) [*Acinetobacter* IRMCBCU95U | 469.3674]						
	PROJECT ID	ACCESSION ID	ORGANISMS	CLASS	PROTEIN FUNCTION	PROTEIN ID	%IDENTITY
**Matched Family**	17477	CP000521	*Acinetobacter baumannii* ATCC 17978, complete genome.	Gammaproteobacteria	putative flavohemoprotein	ABO13482	100.0

**Input Sequence**	fig|469.3674.peg.496 Lyzozyme M1 (1,4-beta-N-acetylmuramidase) (EC 3.2.1.17) [*Acinetobacter* IRMCBCU95U | 469.3674]

	PROJECT ID	ACCESSION ID	ORGANISMS	CLASS	PROTEIN FUNCTION	PROTEIN ID	%IDENTITY
**Matched Family**	17477	CP000521	*Acinetobacter baumannii* ATCC 17978, complete genome.	Gammaproteobacteria	putative lysozyme	ABO10668	100.0

**Input Sequence**	fig|469.3674.peg.5300 Probable DNA repair exonuclease [*Acinetobacter* IRMCBCU95U | 469.3674]						
	PROJECT ID	ACCESSION ID	ORGANISMS	CLASS	PROTEIN FUNCTION	PROTEIN ID	%IDENTITY
**Matched Family**	17827	CP000863	*Acinetobacter baumannii* ACICU, complete genome.	Gammaproteobacteria	predicted phosphohydrolase	ACC58223	100.0

**Input Sequence**	fig|469.3674.peg.2046 hypothetical protein [*Acinetobacter* IRMCBCU95U | 469.3674]						
	PROJECT ID	ACCESSION ID	ORGANISMS	CLASS	PROTEIN FUNCTION	PROTEIN ID	%IDENTITY
**Matched Family**	17477	CP000521	*Acinetobacter baumannii* ATCC 17978, complete genome.	Gammaproteobacteria	hypothetical protein	ABO10767	100.0

**Input Sequence**	fig|469.3674.peg.5815 Carbohydrate Esterase Family 4 [*Acinetobacter* IRMCBCU95U | 469.3674]						
	PROJECT ID	ACCESSION ID	ORGANISMS	CLASS	PROTEIN FUNCTION	PROTEIN ID	%IDENTITY
**Matched Family**	21111	CP001182	*Acinetobacter baumannii* AB0057,	Gammaproteobacteria	putative polysaccharide deacetylase	ACJ41976	100.0

		complete genome.				

Input Sequence	fig|469.3674.peg.1197 Copper resistance protein B [*Acinetobacter* IRMCBCU95U | 469.3674]						
	PROJECT ID	ACCESSION ID	ORGANISMS	CLASS	PROTEIN FUNCTION	PROTEIN ID	%IDENTITY
**Matched Family**	17477	CP000521	*Acinetobacter baumannii* ATCC 17978, complete genome.	Gammaproteobacteria	copper resistance protein B precursor	ABO11152	100.0

Input Sequence	fig|469.3674.peg.3307 Pyrroloquinoline quinone (Coenzyme PQQ) biosynthesis protein C [*Acinetobacter* IRMCBCU95U | 469.3674]						
	PROJECT ID	ACCESSION ID	ORGANISMS	CLASS	PROTEIN FUNCTION	PROTEIN ID	%IDENTITY
**Matched Family**	17827	CP000863	*Acinetobacter baumannii* ACICU, complete genome.	Gammaproteobacteria	Pyrroloquinoline quinone (Coenzyme PQQ) biosynthesis protein C	ACC57044	100.0

Input Sequence	fig|469.3674.peg.1371 Biotin--protein ligase (EC 6.3.4.9)(EC 6.3.4.10)(EC 6.3.4.11)(EC 6.3.4.15) [*Acinetobacter* IRMCBCU95U | 469.3674]						
	PROJECT ID	ACCESSION ID	ORGANISMS	CLASS	PROTEIN FUNCTION	PROTEIN ID	%IDENTITY
**Matched Family**	17827	CP000863	*Acinetobacter baumannii* ACICU, complete genome.	Gammaproteobacteria	Biotin-(acetyl-CoA carboxylase) ligase	ACC56065	100.0

**Input**	fig|469.3674.peg.1398 Malonyl-[acyl-carrier protein] O-methyltransferase (EC 2.1.1.197) [*Acinetobacter* IRMCBCU95U | 469.3674]

**Sequence**							
	PROJECT ID	ACCESSION ID	ORGANISMS	CLASS	PROTEIN FUNCTION	PROTEIN ID	%IDENTITY
**Matched Family**	17827	CP000863	*Acinetobacter baumannii* ACICU, complete genome.	Gammaproteobacteria	SAM-dependent methyltransferase	ACC56078	100.0

**Input Sequence**	fig|469.3674.peg.5561 Peptidase S24 S26A and S26B [*Acinetobacter* IRMCBCU95U | 469.3674]						
	PROJECT ID	ACCESSION ID	ORGANISMS	CLASS	PROTEIN FUNCTION	PROTEIN ID	%IDENTITY
**Matched Family**	21111	CP001182	*Acinetobacter baumannii* AB0057, complete genome.	Gammaproteobacteria	peptidase S24 S26A and S26B	ACJ42079	100.0

**Input Sequence**	fig|469.3674.peg.2379 NADH pyrophosphatase (EC 3.6.1.22), decaps 5'-NAD modified RNA [*Acinetobacter* IRMCBCU95U | 469.3674]						
	PROJECT ID	ACCESSION ID	ORGANISMS	CLASS	PROTEIN FUNCTION	PROTEIN ID	%IDENTITY
**Matched Family**	13001	XXX	Proteus mirabilis HI4320	Gammaproteobacteria	XXX		100.0

**Input Sequence**	fig|469.3674.peg.1439 FIG00003370: Multicopper polyphenol oxidase [*Acinetobacter* IRMCBCU95U | 469.3674]						
	PROJECT ID	ACCESSION ID	ORGANISMS	CLASS	PROTEIN FUNCTION	PROTEIN ID	%IDENTITY
**Matched Family**	17827	CP000863	*Acinetobacter baumannii* ACICU, complete genome.	Gammaproteobacteria	uncharacterized conserved protein	ACC56107	100.0

Input Sequence	fig|469.3674.peg.4825 Putative thioesterase [*Acinetobacter* IRMCBCU95U | 469.3674]						
	PROJECT ID	ACCESSION ID	ORGANISMS	CLASS	PROTEIN FUNCTION	PROTEIN ID	%IDENTITY
Matched Family	17827	CP000863	*Acinetobacter baumannii* ACICU, complete genome.	Gammaproteobacteria	probable acinetobactin biosynthesis protein	ACC57884	100.0

Input Sequence	fig|469.3674.peg.4946 High frequency lysogenization protein HflD [*Acinetobacter* IRMCBCU95U | 469.3674]						
	PROJECT ID	ACCESSION ID	ORGANISMS	CLASS	PROTEIN FUNCTION	PROTEIN ID	%IDENTITY
Matched Family	13001	XXX	Proteus mirabilis HI4320	Gammaproteobacteria	XXX		100.0

Input Sequence	fig|469.3674.peg.3134 hypothetical protein [*Acinetobacter* IRMCBCU95U | 469.3674]						
	PROJECT ID	ACCESSION ID	ORGANISMS	CLASS	PROTEIN FUNCTION	PROTEIN ID	%IDENTITY
Matched Family	17827	CP000863	*Acinetobacter baumannii* ACICU, complete genome.	Gammaproteobacteria	uncharacterized conserved protein	ACC56952	100.0

Input Sequence	fig|469.3674.peg.6647 hypothetical protein [*Acinetobacter* IRMCBCU95U | 469.3674]						
	PROJECT ID	ACCESSION ID	ORGANISMS	CLASS	PROTEIN FUNCTION	PROTEIN ID	%IDENTITY

**Matched Family**	17477	CP000521	*Acinetobacter baumannii* ATCC 17978, complete genome.	Gammaproteobacteria	hypothetical protein	ABO13848	100.0

**Input Sequence**	fig|469.3674.peg.4212 UDP-2,3-diacylglucosamine diphosphatase (EC 3.6.1.54) [*Acinetobacter* IRMCBCU95U | 469.3674]						
	PROJECT ID	ACCESSION ID	ORGANISMS	CLASS	PROTEIN FUNCTION	PROTEIN ID	%IDENTITY
**Matched Family**	17827	CP000863	*Acinetobacter baumannii* ACICU, complete genome.	Gammaproteobacteria	uncharacterized protein conserved in bacteria	ACC57618	100.0

**Input Sequence**	fig|469.3674.peg.916 hypothetical protein [*Acinetobacter* IRMCBCU95U | 469.3674]						
	PROJECT ID	ACCESSION ID	ORGANISMS	CLASS	PROTEIN FUNCTION	PROTEIN ID	%IDENTITY
**Matched Family**	17827	CP000863	*Acinetobacter baumannii* ACICU, complete genome.	Gammaproteobacteria	hypothetical protein	ACC55801	100.0

**Input Sequence**	fig|469.3674.peg.4435 L-Proline/Glycine betaine transporter ProP [*Acinetobacter* IRMCBCU95U | 469.3674]						
	PROJECT ID	ACCESSION ID	ORGANISMS	CLASS	PROTEIN FUNCTION	PROTEIN ID	%IDENTITY
**Matched Family**	17827	CP000863	*Acinetobacter baumannii* ACICU, complete genome.	Gammaproteobacteria	Permease of the major facilitator superfamily	ACC57724	100.0

Input Sequence	fig|469.3674.peg.4453 Transcriptional regulator, GntR family [*Acinetobacter* IRMCBCU95U | 469.3674]						
	PROJECT ID	ACCESSION ID	ORGANISMS	CLASS	PROTEIN FUNCTION	PROTEIN ID	%IDENTITY
**Matched Family**	17827	CP000863	*Acinetobacter baumannii* ACICU, complete genome.	Gammaproteobacteria	Transcriptional regulator	ACC57735	100.0

Input Sequence	fig|469.3674.peg.4599 Exodeoxyribonuclease X [*Acinetobacter* IRMCBCU95U | 469.3674]						
	PROJECT ID	ACCESSION ID	ORGANISMS	CLASS	PROTEIN FUNCTION	PROTEIN ID	%IDENTITY
**Matched Family**	21111	CP001182	*Acinetobacter baumannii* AB0057, complete genome.	Gammaproteobacteria	exodeoxyribonuclease X, putative	ACJ42772	100.0

Input Sequence	fig|469.3674.peg.4232 SSU rRNA pseudouridine(516) synthase (EC 5.4.99.19) [*Acinetobacter* IRMCBCU95U | 469.3674]						
	PROJECT ID	ACCESSION ID	ORGANISMS	CLASS	PROTEIN FUNCTION	PROTEIN ID	%IDENTITY
**Matched Family**	17827	CP000863	*Acinetobacter baumannii* ACICU, complete genome.	Gammaproteobacteria	16S rRNA uridine-516 pseudouridylate synthase	ACC57629	100.0

Input Sequence	fig|469.3674.peg.6871 Transcriptional regulator, DeoR family [*Acinetobacter* IRMCBCU95U | 469.3674]						
	PROJECT ID	ACCESSION ID	ORGANISMS	CLASS	PROTEIN FUNCTION	PROTEIN ID	%IDENTITY
**Matched**	17477	CP000521	*Acinetobacter*	Gammaproteobacteria	putative transcriptional regulator	ABO10862	100.0

**Family**			*baumannii* ATCC 17978, complete genome.				

**Input Sequence**	fig|469.3674.peg.1374 Transcriptional regulator, GntR family [*Acinetobacter* IRMCBCU95U | 469.3674]						
	PROJECT ID	ACCESSION ID	ORGANISMS	CLASS	PROTEIN FUNCTION	PROTEIN ID	%IDENTITY
**Matched Family**	13001	XXX	Proteus mirabilis HI4320	Gammaproteobacteria	XXX		100.0

**Input Sequence**	fig|469.3674.peg.5956 Nitroreductase family protein [*Acinetobacter* IRMCBCU95U | 469.3674]						
	PROJECT ID	ACCESSION ID	ORGANISMS	CLASS	PROTEIN FUNCTION	PROTEIN ID	%IDENTITY
**Matched Family**	17827	CP000863	*Acinetobacter baumannii* ACICU, complete genome.	Gammaproteobacteria	predicted oxidoreductase related to nitroreductase	ACC58550	100.0

**Input Sequence**	fig|469.3674.peg.3474 hypothetical protein [*Acinetobacter* IRMCBCU95U | 469.3674]						
	PROJECT ID	ACCESSION ID	ORGANISMS	CLASS	PROTEIN FUNCTION	PROTEIN ID	%IDENTITY
**Matched Family**	17827	CP000863	*Acinetobacter baumannii* ACICU, complete genome.	Gammaproteobacteria	putative membrane protein	ACC57128	100.0

**Input**	fig|469.3674.peg.3953 HAD-superfamily hydrolase, subfamily IA, variant 3 [*Acinetobacter* IRMCBCU95U | 469.3674]

Sequence							
	PROJECT ID	ACCESSION ID	ORGANISMS	CLASS	PROTEIN FUNCTION	PROTEIN ID	%IDENTITY
**Matched Family**	17827	CP000863	*Acinetobacter baumannii* ACICU, complete genome.	Gammaproteobacteria	predicted hydrolase (HAD superfamily)	ACC57395	100.0

**Input Sequence**	fig|469.3674.peg.4174 Short-chain dehydrogenase, associated with 2-hydroxychromene-2-carboxylate isomerase family protein [*Acinetobacter* IRMCBCU95U | 469.3674]						
	PROJECT ID	ACCESSION ID	ORGANISMS	CLASS	PROTEIN FUNCTION	PROTEIN ID	%IDENTITY
**Matched Family**	17827	CP000863	*Acinetobacter baumannii* ACICU, complete genome.	Gammaproteobacteria	putative oxidoreductase, short-chain dehydrogenase/reductase family	ACC57597	100.0

**Input Sequence**	fig|469.3674.peg.6039 Phospholipid ABC transporter substrate-binding protein MlaD [*Acinetobacter* IRMCBCU95U | 469.3674]						
	PROJECT ID	ACCESSION ID	ORGANISMS	CLASS	PROTEIN FUNCTION	PROTEIN ID	%IDENTITY
**Matched Family**	17477	CP000521	*Acinetobacter baumannii* ATCC 17978, complete genome.	Gammaproteobacteria	toluene tolerance efflux transporter	ABO13498	100.0

**Input Sequence**	fig|469.3674.peg.5436 hypothetical protein [*Acinetobacter* IRMCBCU95U | 469.3674]						
	PROJECT ID	ACCESSION ID	ORGANISMS	CLASS	PROTEIN FUNCTION	PROTEIN ID	%IDENTITY
**Matched**	17827	CP000863	*Acinetobacter*	Gammaproteobacteria	hypothetical protein	ACC58306	100.0

**Family**			*baumannii* ACICU, complete genome.				

**Input Sequence**	fig|469.3674.peg.5940 hypothetical protein [*Acinetobacter* IRMCBCU95U | 469.3674]						
	PROJECT ID	ACCESSION ID	ORGANISMS	CLASS	PROTEIN FUNCTION	PROTEIN ID	%IDENTITY
**Matched Family**	17827	CP000863	*Acinetobacter baumannii* ACICU, complete genome.	Gammaproteobacteria	hypothetical protein	ACC58541	100.0

**Input Sequence**	fig|469.3674.peg.6507 Transcriptional regulator, AcrR family [*Acinetobacter* IRMCBCU95U | 469.3674]						
	PROJECT ID	ACCESSION ID	ORGANISMS	CLASS	PROTEIN FUNCTION	PROTEIN ID	%IDENTITY
**Matched Family**	17477	CP000521	*Acinetobacter baumannii* ATCC 17978, complete genome.	Gammaproteobacteria	putative transcriptional regulator (TetR family)	ABO13762	100.0

**Input Sequence**	fig|469.3674.peg.3899 FIG00351405: hypothetical protein [*Acinetobacter* IRMCBCU95U | 469.3674]						
	PROJECT ID	ACCESSION ID	ORGANISMS	CLASS	PROTEIN FUNCTION	PROTEIN ID	%IDENTITY
**Matched Family**	17827	CP000863	*Acinetobacter baumannii* ACICU, complete genome.	Gammaproteobacteria	hypothetical protein	ACC57355	100.0

**Input Sequence**	fig|469.3674.peg.6962 Ubiquinone biosynthesis protein UbiJ [*Acinetobacter* IRMCBCU95U | 469.3674]						
	PROJECT ID	ACCESSION ID	ORGANISMS	CLASS	PROTEIN FUNCTION	PROTEIN ID	%IDENTITY
**Matched Family**	17477	CP000521	*Acinetobacter baumannii* ATCC 17978, complete genome.	Gammaproteobacteria	hypothetical protein	ABO10809	100.0

**Input Sequence**	fig|469.3674.peg.5517 hypothetical protein [*Acinetobacter* IRMCBCU95U | 469.3674]						
	PROJECT ID	ACCESSION ID	ORGANISMS	CLASS	PROTEIN FUNCTION	PROTEIN ID	%IDENTITY
**Matched Family**	21111	CP001182	*Acinetobacter baumannii* AB0057, complete genome.	Gammaproteobacteria	hypothetical protein	ACJ42047	100.0

**Input Sequence**	fig|469.3674.peg.1255 Transcriptional regulator, AcrR family [*Acinetobacter* IRMCBCU95U | 469.3674]						
	PROJECT ID	ACCESSION ID	ORGANISMS	CLASS	PROTEIN FUNCTION	PROTEIN ID	%IDENTITY
**Matched Family**	17827	CP000863	*Acinetobacter baumannii* ACICU, complete genome.	Gammaproteobacteria	putative transcriptional regulator (TetR family)	ACC55992	100.0

**Input Sequence**	fig|469.3674.peg.995 4'-phosphopantetheinyl transferase (EC 2.7.8.-) [*Acinetobacter* IRMCBCU95U | 469.3674]						
	PROJECT ID	ACCESSION ID	ORGANISMS	CLASS	PROTEIN FUNCTION	PROTEIN ID	%IDENTITY

**Matched Family**	17827	CP000863	*Acinetobacter baumannii* ACICU, complete genome.	Gammaproteobacteria	Phosphopantetheinyl transferase	ACC55848	100.0

**Input Sequence**	fig|469.3674.peg.6614 Collagenase and related proteases [*Acinetobacter* IRMCBCU95U | 469.3674]						
	PROJECT ID	ACCESSION ID	ORGANISMS	CLASS	PROTEIN FUNCTION	PROTEIN ID	%IDENTITY
**Matched Family**	13001	XXX	Proteus mirabilis HI4320	Gammaproteobacteria	XXX		100.0

**Input Sequence**	fig|469.3674.peg.4605 hypothetical protein [*Acinetobacter* IRMCBCU95U | 469.3674]						
	PROJECT ID	ACCESSION ID	ORGANISMS	CLASS	PROTEIN FUNCTION	PROTEIN ID	%IDENTITY
**Matched Family**	13001	XXX	Proteus mirabilis HI4320	Gammaproteobacteria	XXX		100.0

**Input Sequence**	fig|469.3674.peg.3175 putative membrane protein [*Acinetobacter* IRMCBCU95U | 469.3674]						
	PROJECT ID	ACCESSION ID	ORGANISMS	CLASS	PROTEIN FUNCTION	PROTEIN ID	%IDENTITY
**Matched Family**	17477	CP000521	*Acinetobacter baumannii* ATCC 17978, complete genome.	Gammaproteobacteria	putative membrane protein	ABO12062	100.0

**Input**	fig|469.3674.peg.3248 Transcriptional regulator YbiH, TetR family [*Acinetobacter* IRMCBCU95U | 469.3674]

Sequence							
	PROJECT ID	ACCESSION ID	ORGANISMS	CLASS	PROTEIN FUNCTION	PROTEIN ID	%IDENTITY
**Matched Family**	17827	CP000863	*Acinetobacter baumannii* ACICU, complete genome.	Gammaproteobacteria	putative TetR-family transcriptional regulator	ACC57010	100.0

**Input Sequence**	fig|469.3674.peg.4004 hypothetical protein [*Acinetobacter* IRMCBCU95U | 469.3674]						
	PROJECT ID	ACCESSION ID	ORGANISMS	CLASS	PROTEIN FUNCTION	PROTEIN ID	%IDENTITY
**Matched Family**	13001	XXX	Proteus mirabilis HI4320	Gammaproteobacteria	XXX		100.0

**Input Sequence**	fig|469.3674.peg.526 Transcriptional regulator, AcrR family [*Acinetobacter* IRMCBCU95U | 469.3674]						
	PROJECT ID	ACCESSION ID	ORGANISMS	CLASS	PROTEIN FUNCTION	PROTEIN ID	%IDENTITY
**Matched Family**	17827	CP000863	*Acinetobacter baumannii* ACICU, complete genome.	Gammaproteobacteria	Transcriptional regulator	ACC55497	100.0

**Input Sequence**	fig|469.3674.peg.5951 Fumarylacetoacetate hydrolase family protein [*Acinetobacter* IRMCBCU95U | 469.3674]						
	PROJECT ID	ACCESSION ID	ORGANISMS	CLASS	PROTEIN FUNCTION	PROTEIN ID	%IDENTITY
**Matched Family**	13001	XXX	Proteus mirabilis HI4320	Gammaproteobacteria	XXX		100.0

Input Sequence	fig|469.3674.peg.4950 Integral membrane protein [*Acinetobacter* IRMCBCU95U | 469.3674]						
	PROJECT ID	ACCESSION ID	ORGANISMS	CLASS	PROTEIN FUNCTION	PROTEIN ID	%IDENTITY
**Matched Family**	17827	CP000863	*Acinetobacter baumannii* ACICU, complete genome.	Gammaproteobacteria	predicted membrane protein	ACC57960	100.0

Input Sequence	fig|469.3674.peg.6032 Competence protein F homolog, phosphoribosyltransferase domain; protein YhgH required for utilization of DNA as sole source of carbon and energy [*Acinetobacter* IRMCBCU95U | 469.3674]						
	PROJECT ID	ACCESSION ID	ORGANISMS	CLASS	PROTEIN FUNCTION	PROTEIN ID	%IDENTITY
**Matched Family**	28921	XXX	*Acinetobacter baumannii* AYE	Gammaproteobacteria	XXX		100.0

Input Sequence	fig|469.3674.peg.6190 Type IV pilus biogenesis protein PilN [*Acinetobacter* IRMCBCU95U | 469.3674]						
	PROJECT ID	ACCESSION ID	ORGANISMS	CLASS	PROTEIN FUNCTION	PROTEIN ID	%IDENTITY
**Matched Family**	17827	CP000863	*Acinetobacter baumannii* ACICU, complete genome.	Gammaproteobacteria	Tfp pilus assembly protein PilN	ACC58703	100.0

Input Sequence	fig|469.3674.peg.3938 hypothetical protein [*Acinetobacter* IRMCBCU95U | 469.3674]						
	PROJECT ID	ACCESSION ID	ORGANISMS	CLASS	PROTEIN FUNCTION	PROTEIN ID	%IDENTITY

**Matched Family**	17827	CP000863	*Acinetobacter baumannii* ACICU, complete genome.	Gammaproteobacteria	hypothetical protein	ACC57383	100.0

**Input Sequence**	fig|469.3674.peg.4451 Transcriptional regulator, AcrR family [*Acinetobacter* IRMCBCU95U | 469.3674]						
	PROJECT ID	ACCESSION ID	ORGANISMS	CLASS	PROTEIN FUNCTION	PROTEIN ID	%IDENTITY
**Matched Family**	17827	CP000863	*Acinetobacter baumannii* ACICU, complete genome.	Gammaproteobacteria	transcriptional regulator, TetR family	ACC57733	100.0

**Input Sequence**	fig|469.3674.peg.5185 RNA polymerase ECF-type sigma factor [*Acinetobacter* IRMCBCU95U | 469.3674]						
	PROJECT ID	ACCESSION ID	ORGANISMS	CLASS	PROTEIN FUNCTION	PROTEIN ID	%IDENTITY
**Matched Family**	28921	XXX	*Acinetobacter baumannii* AYE	Gammaproteobacteria	XXX		100.0

**Input Sequence**	fig|469.3674.peg.1990 Serine phosphatase RsbU, regulator of sigma subunit [*Acinetobacter* IRMCBCU95U | 469.3674]						
	PROJECT ID	ACCESSION ID	ORGANISMS	CLASS	PROTEIN FUNCTION	PROTEIN ID	%IDENTITY
**Matched Family**	17827	CP000863	*Acinetobacter baumannii* ACICU, complete genome.	Gammaproteobacteria	hypothetical protein	ACC55605	100.0

**Input**	fig|469.3674.peg.2591 hypothetical protein [*Acinetobacter* IRMCBCU95U | 469.3674]

Sequence							
	PROJECT ID	ACCESSION ID	ORGANISMS	CLASS	PROTEIN FUNCTION	PROTEIN ID	%IDENTITY
**Matched Family**	17827	CP000863	*Acinetobacter baumannii* ACICU, complete genome.	Gammaproteobacteria	putative surface antigen	ACC56534	100.0

**Input Sequence**	fig|469.3674.peg.5464 1-acyl-sn-glycerol-3-phosphate acyltransferase [*Acinetobacter* IRMCBCU95U | 469.3674]						
	PROJECT ID	ACCESSION ID	ORGANISMS	CLASS	PROTEIN FUNCTION	PROTEIN ID	%IDENTITY
**Matched Family**	13001	XXX	Proteus mirabilis HI4320	Gammaproteobacteria	XXX		100.0

**Input Sequence**	fig|469.3674.peg.3223 FIG00350035: hypothetical protein [*Acinetobacter* IRMCBCU95U | 469.3674]						
	PROJECT ID	ACCESSION ID	ORGANISMS	CLASS	PROTEIN FUNCTION	PROTEIN ID	%IDENTITY
**Matched Family**	17827	CP000863	*Acinetobacter baumannii* ACICU, complete genome.	Gammaproteobacteria	hypothetical protein	ACC56997	100.0

**Input Sequence**	fig|469.3674.peg.555 Cell division protein [*Acinetobacter* IRMCBCU95U | 469.3674]						
	PROJECT ID	ACCESSION ID	ORGANISMS	CLASS	PROTEIN FUNCTION	PROTEIN ID	%IDENTITY
**Matched Family**	17477	CP000521	*Acinetobacter baumannii* ATCC 17978, complete	Gammaproteobacteria	hypothetical protein	ABO10622	100.0

			genome.				

Input Sequence	fig|469.3674.peg.5426 putative membrane-associated phospholipid phosphatase, PAP2 superfamily [*Acinetobacter* IRMCBCU95U | 469.3674]						
	PROJECT ID	ACCESSION ID	ORGANISMS	CLASS	PROTEIN FUNCTION	PROTEIN ID	%IDENTITY
**Matched Family**	17827	CP000863	*Acinetobacter baumannii* ACICU, complete genome.	Gammaproteobacteria	Membrane-associated phospholipid phosphatase	ACC58298	100.0

Input Sequence	fig|469.3674.peg.1174 Uncharacterized Nudix hydrolase NudL [*Acinetobacter* IRMCBCU95U | 469.3674]						
	PROJECT ID	ACCESSION ID	ORGANISMS	CLASS	PROTEIN FUNCTION	PROTEIN ID	%IDENTITY
**Matched Family**	28921	XXX	*Acinetobacter baumannii* AYE	Gammaproteobacteria	XXX		100.0

Input Sequence	fig|469.3674.peg.4404 putative lipoprotein [*Acinetobacter* IRMCBCU95U | 469.3674]						
	PROJECT ID	ACCESSION ID	ORGANISMS	CLASS	PROTEIN FUNCTION	PROTEIN ID	%IDENTITY
**Matched Family**	17827	CP000863	*Acinetobacter baumannii* ACICU, complete genome.	Gammaproteobacteria	uncharacterized protein conserved in bacteria	ACC57715	100.0

Input Sequence	fig|469.3674.peg.2443 Transcriptional regulator, AcrR family [*Acinetobacter* IRMCBCU95U | 469.3674]						
	PROJECT	ACCESSION	ORGANISMS	CLASS	PROTEIN FUNCTION	PROTEIN	%IDENTITY

	ID	ID				ID	
**Matched Family**	13001	XXX	Proteus mirabilis HI4320	Gammaproteobacteria	XXX		100.0

**Input Sequence**	fig|469.3674.peg.3218 Siderophore synthetase small component, acetyltransferase [*Acinetobacter* IRMCBCU95U | 469.3674]						
	PROJECT ID	ACCESSION ID	ORGANISMS	CLASS	PROTEIN FUNCTION	PROTEIN ID	%IDENTITY
**Matched Family**	17477	CP000521	*Acinetobacter baumannii* ATCC 17978, complete genome.	Gammaproteobacteria	putative siderophore biosynthesis protein; putative acetyltransferase	ABO12084	100.0

**Input Sequence**	fig|469.3674.peg.1879 Cell wall-associated hydrolases (invasion-associated proteins) [*Acinetobacter* IRMCBCU95U | 469.3674]						
	PROJECT ID	ACCESSION ID	ORGANISMS	CLASS	PROTEIN FUNCTION	PROTEIN ID	%IDENTITY
**Matched Family**	13001	XXX	Proteus mirabilis HI4320	Gammaproteobacteria	XXX		100.0

**Input Sequence**	fig|469.3674.peg.5848 Intracellular septation protein IspA [*Acinetobacter* IRMCBCU95U | 469.3674]						
	PROJECT ID	ACCESSION ID	ORGANISMS	CLASS	PROTEIN FUNCTION	PROTEIN ID	%IDENTITY
**Matched Family**	13001	XXX	Proteus mirabilis HI4320	Gammaproteobacteria	XXX		100.0

**Input**	fig|469.3674.peg.4874 MarC family integral membrane protein [*Acinetobacter* IRMCBCU95U | 469.3674]

**Sequence**							
	PROJECT ID	ACCESSION ID	ORGANISMS	CLASS	PROTEIN FUNCTION	PROTEIN ID	%IDENTITY
**Matched Family**	17827	CP000863	*Acinetobacter baumannii* ACICU, complete genome.	Gammaproteobacteria	hypothetical protein	ACC57916	100.0

**Input Sequence**	fig|469.3674.peg.6931 Efflux protein, LysE family [*Acinetobacter* IRMCBCU95U | 469.3674]						
	PROJECT ID	ACCESSION ID	ORGANISMS	CLASS	PROTEIN FUNCTION	PROTEIN ID	%IDENTITY
**Matched Family**	17827	CP000863	*Acinetobacter baumannii* ACICU, complete genome.	Gammaproteobacteria	putative threonine efflux protein	ACC55689	100.0

**Input Sequence**	fig|469.3674.peg.6913 hypothetical protein [*Acinetobacter* IRMCBCU95U | 469.3674]						
	PROJECT ID	ACCESSION ID	ORGANISMS	CLASS	PROTEIN FUNCTION	PROTEIN ID	%IDENTITY
**Matched Family**	13001	XXX	Proteus mirabilis HI4320	Gammaproteobacteria	XXX		100.0

**Input Sequence**	fig|469.3674.peg.510 Lytic transglycosylase [*Acinetobacter* IRMCBCU95U | 469.3674]						
	PROJECT ID	ACCESSION ID	ORGANISMS	CLASS	PROTEIN FUNCTION	PROTEIN ID	%IDENTITY
**Matched Family**	13001	XXX	Proteus mirabilis HI4320	Gammaproteobacteria	XXX		100.0

**Input Sequence**	fig|469.3674.peg.6870 Glutathione S-transferase (EC 2.5.1.18) [*Acinetobacter* IRMCBCU95U | 469.3674]						
	PROJECT ID	ACCESSION ID	ORGANISMS	CLASS	PROTEIN FUNCTION	PROTEIN ID	%IDENTITY
**Matched Family**	17827	CP000863	*Acinetobacter baumannii* ACICU, complete genome.	Gammaproteobacteria	Glutathione S-transferase	ACC55726	100.0

**Input Sequence**	fig|469.3674.peg.4464 Methyltransferase type 12 [*Acinetobacter* IRMCBCU95U | 469.3674]						
	PROJECT ID	ACCESSION ID	ORGANISMS	CLASS	PROTEIN FUNCTION	PROTEIN ID	%IDENTITY
**Matched Family**	17827	CP000863	*Acinetobacter baumannii* ACICU, complete genome.	Gammaproteobacteria	SAM-dependent methyltransferase	ACC57745	100.0

**Input Sequence**	fig|469.3674.peg.556 Glutathione S-transferase (EC 2.5.1.18) [*Acinetobacter* IRMCBCU95U | 469.3674]						
	PROJECT ID	ACCESSION ID	ORGANISMS	CLASS	PROTEIN FUNCTION	PROTEIN ID	%IDENTITY
**Matched Family**	21111	CP001182	*Acinetobacter baumannii* AB0057, complete genome.	Gammaproteobacteria	glutathione S-transferase	ACJ39607	100.0

**Input Sequence**	fig|469.3674.peg.4357 hypothetical protein [*Acinetobacter* IRMCBCU95U | 469.3674]						
	PROJECT ID	ACCESSION ID	ORGANISMS	CLASS	PROTEIN FUNCTION	PROTEIN ID	%IDENTITY

**Matched Family**	17827	CP000863	*Acinetobacter baumannii* ACICU, complete genome.	Gammaproteobacteria	hypothetical protein	ACC57693	100.0

**Input Sequence**	fig|469.3674.peg.5696 DNA-3-methyladenine glycosylase II (EC 3.2.2.21) [*Acinetobacter* IRMCBCU95U | 469.3674]						
	PROJECT ID	ACCESSION ID	ORGANISMS	CLASS	PROTEIN FUNCTION	PROTEIN ID	%IDENTITY
**Matched Family**	28921	XXX	*Acinetobacter baumannii* AYE	Gammaproteobacteria	XXX		100.0

**Input Sequence**	fig|469.3674.peg.1354 Transporter, LysE family [*Acinetobacter* IRMCBCU95U | 469.3674]						
	PROJECT ID	ACCESSION ID	ORGANISMS	CLASS	PROTEIN FUNCTION	PROTEIN ID	%IDENTITY
**Matched Family**	17827	CP000863	*Acinetobacter baumannii* ACICU, complete genome.	Gammaproteobacteria	putative threonine efflux protein	ACC56054	100.0

**Input Sequence**	fig|469.3674.peg.2590 hypothetical protein [*Acinetobacter* IRMCBCU95U | 469.3674]						
	PROJECT ID	ACCESSION ID	ORGANISMS	CLASS	PROTEIN FUNCTION	PROTEIN ID	%IDENTITY
**Matched Family**	17827	CP000863	*Acinetobacter baumannii* ACICU, complete genome.	Gammaproteobacteria	hypothetical protein	ACC56533	100.0

**Input**	fig|469.3674.peg.4068 Response regulator NasT [*Acinetobacter* IRMCBCU95U | 469.3674]

Sequence							
	PROJECT ID	ACCESSION ID	ORGANISMS	CLASS	PROTEIN FUNCTION	PROTEIN ID	%IDENTITY
**Matched Family**	17477	CP000521	*Acinetobacter baumannii* ATCC 17978, complete genome.	Gammaproteobacteria	response regulator protein	ABO12433	100.0

**Input Sequence**	fig|469.3674.peg.7195 hypothetical protein [*Acinetobacter* IRMCBCU95U | 469.3674]						
	PROJECT ID	ACCESSION ID	ORGANISMS	CLASS	PROTEIN FUNCTION	PROTEIN ID	%IDENTITY
**Matched Family**	17827	CP000863	*Acinetobacter baumannii* ACICU, complete genome.	Gammaproteobacteria	hypothetical protein	ACC56264	100.0

**Input Sequence**	fig|469.3674.peg.2520 O-methyltransferase [*Acinetobacter* IRMCBCU95U | 469.3674]						
	PROJECT ID	ACCESSION ID	ORGANISMS	CLASS	PROTEIN FUNCTION	PROTEIN ID	%IDENTITY
**Matched Family**	17827	CP000863	*Acinetobacter baumannii* ACICU, complete genome.	Gammaproteobacteria	predicted O-methyltransferase	ACC56493	100.0

**Input Sequence**	fig|469.3674.peg.4505 Colicin V production protein [*Acinetobacter* IRMCBCU95U | 469.3674]						
	PROJECT ID	ACCESSION ID	ORGANISMS	CLASS	PROTEIN FUNCTION	PROTEIN ID	%IDENTITY
**Matched**	17477	CP000521	*Acinetobacter*	Gammaproteobacteria	putative colicin V producing membrane protein	ABO12675	100.0

**Family**			*baumannii* ATCC 17978, complete genome.				

**Input Sequence**	fig|469.3674.peg.4503 Colicin V production protein [*Acinetobacter* IRMCBCU95U | 469.3674]						
	PROJECT ID	ACCESSION ID	ORGANISMS	CLASS	PROTEIN FUNCTION	PROTEIN ID	%IDENTITY
**Matched Family**	17827	CP000863	*Acinetobacter baumannii* ACICU, complete genome.	Gammaproteobacteria	putative membrane protein required for colicin V production (CvpA)	ACC57765	100.0

**Input Sequence**	fig|469.3674.peg.3008 Transcriptional regulator, AcrR family [*Acinetobacter* IRMCBCU95U | 469.3674]						
	PROJECT ID	ACCESSION ID	ORGANISMS	CLASS	PROTEIN FUNCTION	PROTEIN ID	%IDENTITY
**Matched Family**	17827	CP000863	*Acinetobacter baumannii* ACICU, complete genome.	Gammaproteobacteria	Transcriptional regulator	ACC56898	100.0

**Input Sequence**	fig|469.3674.peg.7287 5-formyltetrahydrofolate cyclo-ligase (EC 6.3.3.2) [*Acinetobacter* IRMCBCU95U | 469.3674]						
	PROJECT ID	ACCESSION ID	ORGANISMS	CLASS	PROTEIN FUNCTION	PROTEIN ID	%IDENTITY
**Matched Family**	17827	CP000863	*Acinetobacter baumannii* ACICU, complete genome.	Gammaproteobacteria	5-formyltetrahydrofolate cyclo-ligase	ACC56307	100.0

**Input Sequence**	fig|469.3674.peg.2031 Outer membrane protein W precursor [*Acinetobacter* IRMCBCU95U | 469.3674]						
	PROJECT ID	ACCESSION ID	ORGANISMS	CLASS	PROTEIN FUNCTION	PROTEIN ID	%IDENTITY
**Matched Family**	17827	CP000863	*Acinetobacter baumannii* ACICU, complete genome.	Gammaproteobacteria	Outer membrane protein W	ACC55622	100.0

**Input Sequence**	fig|469.3674.peg.6922 General secretion pathway protein G [*Acinetobacter* IRMCBCU95U | 469.3674]						
	PROJECT ID	ACCESSION ID	ORGANISMS	CLASS	PROTEIN FUNCTION	PROTEIN ID	%IDENTITY
**Matched Family**	17827	CP000863	*Acinetobacter baumannii* ACICU, complete genome.	Gammaproteobacteria	Type II secretory pathway, pseudopilin PulG	ACC55694	100.0

**Input Sequence**	fig|469.3674.peg.4514 Nitroreductase family protein [*Acinetobacter* IRMCBCU95U | 469.3674]						
	PROJECT ID	ACCESSION ID	ORGANISMS	CLASS	PROTEIN FUNCTION	PROTEIN ID	%IDENTITY
**Matched Family**	17827	CP000863	*Acinetobacter baumannii* ACICU, complete genome.	Gammaproteobacteria	Nitroreductase	ACC57771	100.0

**Input Sequence**	fig|469.3674.peg.5638 putative membrane protein [*Acinetobacter* IRMCBCU95U | 469.3674]						
	PROJECT ID	ACCESSION ID	ORGANISMS	CLASS	PROTEIN FUNCTION	PROTEIN ID	%IDENTITY
**Matched**	13001	XXX	Proteus mirabilis	Gammaproteobacteria	XXX		100.0

Family			HI4320				

Input Sequence	fig|469.3674.peg.2971 hypothetical protein [*Acinetobacter* IRMCBCU95U | 469.3674]						
	PROJECT ID	ACCESSION ID	ORGANISMS	CLASS	PROTEIN FUNCTION	PROTEIN ID	%IDENTITY
**Matched Family**	17827	CP000864	*Acinetobacter baumannii* ACICU plasmid pACICU1, complete sequence.	Gammaproteobacteria	DNA replication protein	ACC58981	100.0

Input Sequence	fig|469.3674.peg.4088 Transcriptional regulator, AcrR family [*Acinetobacter* IRMCBCU95U | 469.3674]						
	PROJECT ID	ACCESSION ID	ORGANISMS	CLASS	PROTEIN FUNCTION	PROTEIN ID	%IDENTITY
**Matched Family**	17827	CP000863	*Acinetobacter baumannii* ACICU, complete genome.	Gammaproteobacteria	Transcriptional regulator	ACC57549	100.0

Input Sequence	fig|469.3674.peg.1340 Transcriptional regulator, AcrR family [*Acinetobacter* IRMCBCU95U | 469.3674]						
	PROJECT ID	ACCESSION ID	ORGANISMS	CLASS	PROTEIN FUNCTION	PROTEIN ID	%IDENTITY
**Matched Family**	17477	CP000521	*Acinetobacter baumannii* ATCC 17978, complete genome.	Gammaproteobacteria	putative transcriptional regulator (TetR-family)	ABO11216	100.0

**Input Sequence**	fig|469.3674.peg.7249 Nickel-binding accessory protein UreJ-HupE [*Acinetobacter* IRMCBCU95U | 469.3674]						
	PROJECT ID	ACCESSION ID	ORGANISMS	CLASS	PROTEIN FUNCTION	PROTEIN ID	%IDENTITY
**Matched Family**	13001	XXX	Proteus mirabilis HI4320	Gammaproteobacteria	XXX		100.0

**Input Sequence**	fig|469.3674.peg.924 Transposase [*Acinetobacter* IRMCBCU95U | 469.3674]						
	PROJECT ID	ACCESSION ID	ORGANISMS	CLASS	PROTEIN FUNCTION	PROTEIN ID	%IDENTITY
**Matched Family**	21111	CP001182	*Acinetobacter baumannii* AB0057, complete genome.	Gammaproteobacteria	transposase 1	ACJ39440	100.0

**Input Sequence**	fig|469.3674.peg.6759 Transposase [*Acinetobacter* IRMCBCU95U | 469.3674]						
	PROJECT ID	ACCESSION ID	ORGANISMS	CLASS	PROTEIN FUNCTION	PROTEIN ID	%IDENTITY
**Matched Family**	21111	CP001182	*Acinetobacter baumannii* AB0057, complete genome.	Gammaproteobacteria	transposase 1	ACJ39971	100.0

**Input Sequence**	fig|469.3674.peg.5967 Membrane protein, Rhomboid family [*Acinetobacter* IRMCBCU95U | 469.3674]						
	PROJECT ID	ACCESSION ID	ORGANISMS	CLASS	PROTEIN FUNCTION	PROTEIN ID	%IDENTITY
**Matched Family**	17827	CP000863	*Acinetobacter baumannii* ACICU,	Gammaproteobacteria	uncharacterized membrane protein	ACC58558	100.0

		complete genome.				

Input Sequence	fig|469.3674.peg.1352 COGs COG3216 [*Acinetobacter* IRMCBCU95U | 469.3674]						
	PROJECT ID	ACCESSION ID	ORGANISMS	CLASS	PROTEIN FUNCTION	PROTEIN ID	%IDENTITY
**Matched Family**	17827	CP000863	*Acinetobacter baumannii* ACICU, complete genome.	Gammaproteobacteria	uncharacterized protein conserved in bacteria	ACC56053	100.0

Input Sequence	fig|469.3674.peg.1825 Transcriptional regulator, AcrR family [*Acinetobacter* IRMCBCU95U | 469.3674]						
	PROJECT ID	ACCESSION ID	ORGANISMS	CLASS	PROTEIN FUNCTION	PROTEIN ID	%IDENTITY
**Matched Family**	17827	CP000863	*Acinetobacter baumannii* ACICU, complete genome.	Gammaproteobacteria	Transcriptional regulator	ACC56850	100.0

Input Sequence	fig|469.3674.peg.1870 Protein affecting phage T7 exclusion by the F plasmid [*Acinetobacter* IRMCBCU95U | 469.3674]						
	PROJECT ID	ACCESSION ID	ORGANISMS	CLASS	PROTEIN FUNCTION	PROTEIN ID	%IDENTITY
**Matched Family**	13001	XXX	Proteus mirabilis HI4320	Gammaproteobacteria	XXX		100.0

Input Sequence	fig|469.3674.peg.6166 FIG004694: Hypothetical protein [*Acinetobacter* IRMCBCU95U | 469.3674]						
	PROJECT	ACCESSION	ORGANISMS	CLASS	PROTEIN FUNCTION	PROTEIN	%IDENTITY

	ID	ID				ID	
**Matched Family**	17827	CP000863	*Acinetobacter baumannii* ACICU, complete genome.	Gammaproteobacteria	predicted membrane protein	ACC58691	100.0

**Input Sequence**	fig|469.3674.peg.6721 DedA protein [*Acinetobacter* IRMCBCU95U | 469.3674]						
	PROJECT ID	ACCESSION ID	ORGANISMS	CLASS	PROTEIN FUNCTION	PROTEIN ID	%IDENTITY
**Matched Family**	17477	CP000521	*Acinetobacter baumannii* ATCC 17978, complete genome.	Gammaproteobacteria	putative DedA family protein	ABO10503	100.0

**Input Sequence**	fig|469.3674.peg.917 hypothetical protein; putative signal peptide [*Acinetobacter* IRMCBCU95U | 469.3674]						
	PROJECT ID	ACCESSION ID	ORGANISMS	CLASS	PROTEIN FUNCTION	PROTEIN ID	%IDENTITY
**Matched Family**	13001	XXX	Proteus mirabilis HI4320	Gammaproteobacteria	XXX		100.0

**Input Sequence**	fig|469.3674.peg.6656 Heat shock protein GrpE [*Acinetobacter* IRMCBCU95U | 469.3674]						
	PROJECT ID	ACCESSION ID	ORGANISMS	CLASS	PROTEIN FUNCTION	PROTEIN ID	%IDENTITY
**Matched Family**	17477	CP000521	*Acinetobacter baumannii* ATCC 17978, complete genome.	Gammaproteobacteria	Hsp 24 nucleotide exchange factor	ABO13362	100.0

Input Sequence	fig|469.3674.peg.7023 UPF0301 protein YqgE [*Acinetobacter* IRMCBCU95U | 469.3674]						
	PROJECT ID	ACCESSION ID	ORGANISMS	CLASS	PROTEIN FUNCTION	PROTEIN ID	%IDENTITY
Matched Family	13001	XXX	Proteus mirabilis HI4320	Gammaproteobacteria	XXX		100.0

Input Sequence	fig|469.3674.peg.6464 FIG00350877: hypothetical protein [*Acinetobacter* IRMCBCU95U | 469.3674]						
	PROJECT ID	ACCESSION ID	ORGANISMS	CLASS	PROTEIN FUNCTION	PROTEIN ID	%IDENTITY
Matched Family	21111	CP001182	*Acinetobacter baumannii* AB0057, complete genome.	Gammaproteobacteria	conserved hypothetical protein	ACJ43151	100.0

Input Sequence	fig|469.3674.peg.7327 FIG00350917: hypothetical protein [*Acinetobacter* IRMCBCU95U | 469.3674]						
	PROJECT ID	ACCESSION ID	ORGANISMS	CLASS	PROTEIN FUNCTION	PROTEIN ID	%IDENTITY
Matched Family	17827	CP000863	*Acinetobacter baumannii* ACICU, complete genome.	Gammaproteobacteria	hypothetical protein	ACC56337	100.0

Input Sequence	fig|469.3674.peg.1307 NADH ubiquinone oxidoreductase chain A (EC 1.6.5.3) [*Acinetobacter* IRMCBCU95U | 469.3674]						
	PROJECT ID	ACCESSION ID	ORGANISMS	CLASS	PROTEIN FUNCTION	PROTEIN ID	%IDENTITY

**Matched Family**	13001	XXX	Proteus mirabilis HI4320	Gammaproteobacteria	XXX		100.0

**Input Sequence**	fig|469.3674.peg.4261 ATPase involved in DNA repair [*Acinetobacter* IRMCBCU95U | 469.3674]						
	PROJECT ID	ACCESSION ID	ORGANISMS	CLASS	PROTEIN FUNCTION	PROTEIN ID	%IDENTITY
**Matched Family**	17477	CP000521	*Acinetobacter baumannii* ATCC 17978, complete genome.	Gammaproteobacteria	putative signal peptide	ABO12562	100.0

**Input Sequence**	fig|469.3674.peg.985 hypothetical protein [*Acinetobacter* IRMCBCU95U | 469.3674]						
	PROJECT ID	ACCESSION ID	ORGANISMS	CLASS	PROTEIN FUNCTION	PROTEIN ID	%IDENTITY
**Matched Family**	17827	CP000863	*Acinetobacter baumannii* ACICU, complete genome.	Gammaproteobacteria	hypothetical protein	ACC55842	100.0

**Input Sequence**	fig|469.3674.peg.2969 hypothetical protein [*Acinetobacter* IRMCBCU95U | 469.3674]						
	PROJECT ID	ACCESSION ID	ORGANISMS	CLASS	PROTEIN FUNCTION	PROTEIN ID	%IDENTITY
**Matched Family**	17827	CP000864	*Acinetobacter baumannii* ACICU plasmid pACICU1, complete sequence.	Gammaproteobacteria	TPR repeat, SEL1 subfamily protein	ACC58992	100.0

**Input Sequence**	fig|469.3674.peg.7057 hypothetical protein [*Acinetobacter* IRMCBCU95U | 469.3674]						
	PROJECT ID	ACCESSION ID	ORGANISMS	CLASS	PROTEIN FUNCTION	PROTEIN ID	%IDENTITY
**Matched Family**	17827	CP000863	*Acinetobacter baumannii* ACICU, complete genome.	Gammaproteobacteria	hypothetical protein	ACC55632	100.0

**Input Sequence**	fig|469.3674.peg.4450 Sigma-fimbriae uncharacterized paralogous subunit [*Acinetobacter* IRMCBCU95U | 469.3674]						
	PROJECT ID	ACCESSION ID	ORGANISMS	CLASS	PROTEIN FUNCTION	PROTEIN ID	%IDENTITY
**Matched Family**	28921	XXX	*Acinetobacter baumannii* AYE	Gammaproteobacteria	XXX		100.0

**Input Sequence**	fig|469.3674.peg.5323 hypothetical protein [*Acinetobacter* IRMCBCU95U | 469.3674]						
	PROJECT ID	ACCESSION ID	ORGANISMS	CLASS	PROTEIN FUNCTION	PROTEIN ID	%IDENTITY
**Matched Family**	17827	CP000863	*Acinetobacter baumannii* ACICU, complete genome.	Gammaproteobacteria	hypothetical protein	ACC58239	100.0

**Input Sequence**	fig|469.3674.peg.1083 Sodium-dependent phosphate transporter [*Acinetobacter* IRMCBCU95U | 469.3674]						
	PROJECT ID	ACCESSION ID	ORGANISMS	CLASS	PROTEIN FUNCTION	PROTEIN ID	%IDENTITY
**Matched Family**	13001	XXX	Proteus mirabilis HI4320	Gammaproteobacteria	XXX		100.0

Input Sequence	fig|469.3674.peg.2707 Uncharacterized NAD(P)H oxidoreductase, YdeQ/YrkL/YwrO family [*Acinetobacter* IRMCBCU95U | 469.3674]						
	PROJECT ID	ACCESSION ID	ORGANISMS	CLASS	PROTEIN FUNCTION	PROTEIN ID	%IDENTITY
**Matched Family**	17827	CP000863	*Acinetobacter baumannii* ACICU, complete genome.	Gammaproteobacteria	putative NADPH-quinone reductase (modulator of drug activity B)	ACC56596	100.0

Input Sequence	fig|469.3674.peg.5782 hypothetical protein [*Acinetobacter* IRMCBCU95U | 469.3674]						
	PROJECT ID	ACCESSION ID	ORGANISMS	CLASS	PROTEIN FUNCTION	PROTEIN ID	%IDENTITY
**Matched Family**	17827	CP000863	*Acinetobacter baumannii* ACICU, complete genome.	Gammaproteobacteria	hypothetical protein	ACC58439	100.0

Input Sequence	fig|469.3674.peg.7252 hypothetical protein [*Acinetobacter* IRMCBCU95U | 469.3674]						
	PROJECT ID	ACCESSION ID	ORGANISMS	CLASS	PROTEIN FUNCTION	PROTEIN ID	%IDENTITY
**Matched Family**	13001	XXX	Proteus mirabilis HI4320	Gammaproteobacteria	XXX		100.0

Input Sequence	fig|469.3674.peg.4318 hypothetical protein [*Acinetobacter* IRMCBCU95U | 469.3674]						
	PROJECT ID	ACCESSION ID	ORGANISMS	CLASS	PROTEIN FUNCTION	PROTEIN ID	%IDENTITY

**Matched Family**	28921	XXX	*Acinetobacter baumannii* AYE	Gammaproteobacteria	XXX		100.0

**Input Sequence**	fig|469.3674.peg.4000 FIG00350469: hypothetical protein [*Acinetobacter* IRMCBCU95U | 469.3674]						
	PROJECT ID	ACCESSION ID	ORGANISMS	CLASS	PROTEIN FUNCTION	PROTEIN ID	%IDENTITY
**Matched Family**	17827	CP000863	*Acinetobacter baumannii* ACICU, complete genome.	Gammaproteobacteria	hypothetical protein	ACC57416	100.0

**Input Sequence**	fig|469.3674.peg.5616 hypothetical protein; putative signal peptide [*Acinetobacter* IRMCBCU95U | 469.3674]						
	PROJECT ID	ACCESSION ID	ORGANISMS	CLASS	PROTEIN FUNCTION	PROTEIN ID	%IDENTITY
**Matched Family**	17827	CP000863	*Acinetobacter baumannii* ACICU, complete genome.	Gammaproteobacteria	hypothetical protein	ACC58350	100.0

**Input Sequence**	fig|469.3674.peg.3168 Chaperone protein HscB [*Acinetobacter* IRMCBCU95U | 469.3674]						
	PROJECT ID	ACCESSION ID	ORGANISMS	CLASS	PROTEIN FUNCTION	PROTEIN ID	%IDENTITY
**Matched Family**	17477	CP000521	*Acinetobacter baumannii* ATCC 17978, complete genome.	Gammaproteobacteria	co-chaperone protein (Hsc20)	ABO12056	100.0

**Input Sequence**	fig|469.3674.peg.121 FIG00350955: hypothetical protein [*Acinetobacter* IRMCBCU95U | 469.3674]						
	PROJECT ID	ACCESSION ID	ORGANISMS	CLASS	PROTEIN FUNCTION	PROTEIN ID	%IDENTITY
**Matched Family**	17827	CP000863	*Acinetobacter baumannii* ACICU, complete genome.	Gammaproteobacteria	hypothetical protein	ACC57498	100.0

**Input Sequence**	fig|469.3674.peg.1046 hypothetical protein [*Acinetobacter* IRMCBCU95U | 469.3674]						
	PROJECT ID	ACCESSION ID	ORGANISMS	CLASS	PROTEIN FUNCTION	PROTEIN ID	%IDENTITY
**Matched Family**	13001	XXX	Proteus mirabilis HI4320	Gammaproteobacteria	XXX		100.0

**Input Sequence**	fig|469.3674.peg.7400 hypothetical protein [*Acinetobacter* IRMCBCU95U | 469.3674]						
	PROJECT ID	ACCESSION ID	ORGANISMS	CLASS	PROTEIN FUNCTION	PROTEIN ID	%IDENTITY
**Matched Family**	17827	CP000863	*Acinetobacter baumannii* ACICU, complete genome.	Gammaproteobacteria	hypothetical protein	ACC56376	100.0

**Input Sequence**	fig|469.3674.peg.2463 putative lipoprotein [*Acinetobacter* IRMCBCU95U | 469.3674]						
	PROJECT ID	ACCESSION ID	ORGANISMS	CLASS	PROTEIN FUNCTION	PROTEIN ID	%IDENTITY
**Matched Family**	28921	XXX	*Acinetobacter baumannii* AYE	Gammaproteobacteria	XXX		100.0

**Input Sequence**	fig|469.3674.peg.3829 internalin, putative [*Acinetobacter* IRMCBCU95U | 469.3674]						
	PROJECT ID	ACCESSION ID	ORGANISMS	CLASS	PROTEIN FUNCTION	PROTEIN ID	%IDENTITY
**Matched Family**	28921	XXX	*Acinetobacter baumannii* AYE	Gammaproteobacteria	XXX		100.0

**Input Sequence**	fig|469.3674.peg.5955 hypothetical protein [*Acinetobacter* IRMCBCU95U | 469.3674]						
	PROJECT ID	ACCESSION ID	ORGANISMS	CLASS	PROTEIN FUNCTION	PROTEIN ID	%IDENTITY
**Matched Family**	28921	XXX	*Acinetobacter baumannii* AYE	Gammaproteobacteria	XXX		100.0

**Input Sequence**	fig|469.3674.peg.5171 Tol-Pal system-associated acyl-CoA thioesterase [*Acinetobacter* IRMCBCU95U | 469.3674]						
	PROJECT ID	ACCESSION ID	ORGANISMS	CLASS	PROTEIN FUNCTION	PROTEIN ID	%IDENTITY
**Matched Family**	13001	XXX	Proteus mirabilis HI4320	Gammaproteobacteria	XXX		100.0

**Input Sequence**	fig|469.3674.peg.7205 FIG00350948: hypothetical protein [*Acinetobacter* IRMCBCU95U | 469.3674]						
	PROJECT ID	ACCESSION ID	ORGANISMS	CLASS	PROTEIN FUNCTION	PROTEIN ID	%IDENTITY
**Matched Family**	13001	XXX	Proteus mirabilis HI4320	Gammaproteobacteria	XXX		100.0

Input Sequence	fig|469.3674.peg.724 Putative transmembrane protein [*Acinetobacter* IRMCBCU95U | 469.3674]						
	PROJECT ID	ACCESSION ID	ORGANISMS	CLASS	PROTEIN FUNCTION	PROTEIN ID	%IDENTITY
**Matched Family**	28921	XXX	*Acinetobacter baumannii* AYE	Gammaproteobacteria	XXX		100.0

Input Sequence	fig|469.3674.peg.3783 hypothetical protein [*Acinetobacter* IRMCBCU95U | 469.3674]						
	PROJECT ID	ACCESSION ID	ORGANISMS	CLASS	PROTEIN FUNCTION	PROTEIN ID	%IDENTITY
**Matched Family**	13001	XXX	Proteus mirabilis HI4320	Gammaproteobacteria	XXX		100.0

Input Sequence	fig|469.3674.peg.5721 Oxidoreductase probably involved in sulfite reduction [*Acinetobacter* IRMCBCU95U | 469.3674]						
	PROJECT ID	ACCESSION ID	ORGANISMS	CLASS	PROTEIN FUNCTION	PROTEIN ID	%IDENTITY
**Matched Family**	17827	CP000863	*Acinetobacter baumannii* ACICU, complete genome.	Gammaproteobacteria	uncharacterized protein conserved in bacteria	ACC58406	100.0

Input Sequence	fig|469.3674.peg.220 PhaK-like protein [*Acinetobacter* IRMCBCU95U | 469.3674]						
	PROJECT ID	ACCESSION ID	ORGANISMS	CLASS	PROTEIN FUNCTION	PROTEIN ID	%IDENTITY
**Matched**	21111	CP001182	*Acinetobacter*	Gammaproteobacteria	putative benzoate transport porin	ACJ40772	100.0

**Family**			*baumannii* AB0057, complete genome.				

**Input Sequence**	fig|469.3674.peg.3959 Regulatory protein RecX [*Acinetobacter* IRMCBCU95U | 469.3674]						
	PROJECT ID	ACCESSION ID	ORGANISMS	CLASS	PROTEIN FUNCTION	PROTEIN ID	%IDENTITY
**Matched Family**	13001	XXX	Proteus mirabilis HI4320	Gammaproteobacteria	XXX		100.0

**Input Sequence**	fig|469.3674.peg.1821 Transcriptional regulator, AsnC family [*Acinetobacter* IRMCBCU95U | 469.3674]						
	PROJECT ID	ACCESSION ID	ORGANISMS	CLASS	PROTEIN FUNCTION	PROTEIN ID	%IDENTITY
**Matched Family**	13001	XXX	Proteus mirabilis HI4320	Gammaproteobacteria	XXX		100.0

**Input Sequence**	fig|469.3674.peg.5850 hypothetical protein; putative signal peptide [*Acinetobacter* IRMCBCU95U | 469.3674]						
	PROJECT ID	ACCESSION ID	ORGANISMS	CLASS	PROTEIN FUNCTION	PROTEIN ID	%IDENTITY
**Matched Family**	17477	CP000521	*Acinetobacter baumannii* ATCC 17978, complete genome.	Gammaproteobacteria	hypothetical protein	ABS90273	100.0

**Input Sequence**	fig|469.3674.peg.5393 hypothetical protein [*Acinetobacter* IRMCBCU95U | 469.3674]

	PROJECT ID	ACCESSION ID	ORGANISMS	CLASS	PROTEIN FUNCTION	PROTEIN ID	%IDENTITY
**Matched Family**	17477	CP000521	*Acinetobacter baumannii* ATCC 17978, complete genome.	Gammaproteobacteria	hypothetical protein	ABO13122	100.0

**Input Sequence**	fig|469.3674.peg.3445 hypothetical protein [*Acinetobacter* IRMCBCU95U | 469.3674]						
	PROJECT ID	ACCESSION ID	ORGANISMS	CLASS	PROTEIN FUNCTION	PROTEIN ID	%IDENTITY
**Matched Family**	17827	CP000863	*Acinetobacter baumannii* ACICU, complete genome.	Gammaproteobacteria	hypothetical protein	ACC57116	100.0

**Input Sequence**	fig|469.3674.peg.3930 Transcriptional regulator, MarR family [*Acinetobacter* IRMCBCU95U | 469.3674]						
	PROJECT ID	ACCESSION ID	ORGANISMS	CLASS	PROTEIN FUNCTION	PROTEIN ID	%IDENTITY
**Matched Family**	17477	CP000521	*Acinetobacter baumannii* ATCC 17978, complete genome.	Gammaproteobacteria	putative transcriptional repressor of for multidrug resistance pump (MarR family)	ABO12375	100.0

**Input Sequence**	fig|469.3674.peg.6888 Integral membrane protein [*Acinetobacter* IRMCBCU95U | 469.3674]						
	PROJECT ID	ACCESSION ID	ORGANISMS	CLASS	PROTEIN FUNCTION	PROTEIN ID	%IDENTITY
**Matched Family**	13001	XXX	Proteus mirabilis HI4320	Gammaproteobacteria	XXX		100.0

Input Sequence	fig|469.3674.peg.3259 FIG00350803: hypothetical protein [*Acinetobacter* IRMCBCU95U | 469.3674]						
	PROJECT ID	ACCESSION ID	ORGANISMS	CLASS	PROTEIN FUNCTION	PROTEIN ID	%IDENTITY
Matched Family	13001	XXX	Proteus mirabilis HI4320	Gammaproteobacteria	XXX		100.0

Input Sequence	fig|469.3674.peg.7375 transcriptional regulator [*Acinetobacter* IRMCBCU95U | 469.3674]						
	PROJECT ID	ACCESSION ID	ORGANISMS	CLASS	PROTEIN FUNCTION	PROTEIN ID	%IDENTITY
Matched Family	21111	CP001182	*Acinetobacter baumannii* AB0057, complete genome.	Gammaproteobacteria	helix-turn-helix domain protein	ACJ41791	100.0

Input Sequence	fig|469.3674.peg.6815 hypothetical protein [*Acinetobacter* IRMCBCU95U | 469.3674]						
	PROJECT ID	ACCESSION ID	ORGANISMS	CLASS	PROTEIN FUNCTION	PROTEIN ID	%IDENTITY
Matched Family	13001	XXX	Proteus mirabilis HI4320	Gammaproteobacteria	XXX		100.0

Input Sequence	fig|469.3674.peg.5798 hypothetical protein [*Acinetobacter* IRMCBCU95U | 469.3674]						
	PROJECT ID	ACCESSION ID	ORGANISMS	CLASS	PROTEIN FUNCTION	PROTEIN ID	%IDENTITY
Matched	17827	CP000863	*Acinetobacter*	Gammaproteobacteria	hypothetical protein	ACC58452	100.0

Family			*baumannii* ACICU, complete genome.				

Input Sequence	fig|469.3674.peg.7127 hypothetical protein [*Acinetobacter* IRMCBCU95U | 469.3674]						
	PROJECT ID	ACCESSION ID	ORGANISMS	CLASS	PROTEIN FUNCTION	PROTEIN ID	%IDENTITY
Matched Family	17827	CP000863	*Acinetobacter baumannii* ACICU, complete genome.	Gammaproteobacteria	hypothetical protein	ACC56239	100.0

Input Sequence	fig|469.3674.peg.926 Mobile element protein [*Acinetobacter* IRMCBCU95U | 469.3674]						
	PROJECT ID	ACCESSION ID	ORGANISMS	CLASS	PROTEIN FUNCTION	PROTEIN ID	%IDENTITY
Matched Family	21111	CP001182	*Acinetobacter baumannii* AB0057, complete genome.	Gammaproteobacteria	transposase subunit	ACJ39443	100.0

Input Sequence	fig|469.3674.peg.4665 hypothetical protein [*Acinetobacter* IRMCBCU95U | 469.3674]						
	PROJECT ID	ACCESSION ID	ORGANISMS	CLASS	PROTEIN FUNCTION	PROTEIN ID	%IDENTITY
Matched Family	17827	CP000863	*Acinetobacter baumannii* ACICU, complete genome.	Gammaproteobacteria	hypothetical protein	ACC57857	100.0

Input	fig|469.3674.peg.4231 Acetyltransferase, GNAT family [*Acinetobacter* IRMCBCU95U | 469.3674]

**Sequence**							
	PROJECT ID	ACCESSION ID	ORGANISMS	CLASS	PROTEIN FUNCTION	PROTEIN ID	%IDENTITY
**Matched Family**	17827	CP000863	*Acinetobacter baumannii* ACICU, complete genome.	Gammaproteobacteria	Histone acetyltransferase HPA2	ACC57628	100.0

**Input Sequence**	fig|469.3674.peg.1834 hypothetical protein [*Acinetobacter* IRMCBCU95U | 469.3674]						
	PROJECT ID	ACCESSION ID	ORGANISMS	CLASS	PROTEIN FUNCTION	PROTEIN ID	%IDENTITY
**Matched Family**	13001	XXX	Proteus mirabilis HI4320	Gammaproteobacteria	XXX		100.0

**Input Sequence**	fig|469.3674.peg.4242 FIG00349963: hypothetical protein [*Acinetobacter* IRMCBCU95U | 469.3674]						
	PROJECT ID	ACCESSION ID	ORGANISMS	CLASS	PROTEIN FUNCTION	PROTEIN ID	%IDENTITY
**Matched Family**	17827	CP000863	*Acinetobacter baumannii* ACICU, complete genome.	Gammaproteobacteria	hypothetical protein	ACC57639	100.0

**Input Sequence**	fig|469.3674.peg.5434 hypothetical protein [*Acinetobacter* IRMCBCU95U | 469.3674]						
	PROJECT ID	ACCESSION ID	ORGANISMS	CLASS	PROTEIN FUNCTION	PROTEIN ID	%IDENTITY
**Matched Family**	17827	CP000863	*Acinetobacter baumannii* ACICU, complete genome.	Gammaproteobacteria	hypothetical protein	ACC58304	100.0

Input Sequence	fig|469.3674.peg.6076 hypothetical protein [*Acinetobacter* IRMCBCU95U | 469.3674]						
	PROJECT ID	ACCESSION ID	ORGANISMS	CLASS	PROTEIN FUNCTION	PROTEIN ID	%IDENTITY
**Matched Family**	17477	CP000521	*Acinetobacter baumannii* ATCC 17978, complete genome.	Gammaproteobacteria	putative signal peptide	ABO13522	100.0

Input Sequence	fig|469.3674.peg.988 3-hydroxydecanoyl-[ACP] dehydratase (EC 4.2.1.59) [*Acinetobacter* IRMCBCU95U | 469.3674]						
	PROJECT ID	ACCESSION ID	ORGANISMS	CLASS	PROTEIN FUNCTION	PROTEIN ID	%IDENTITY
**Matched Family**	17827	CP000863	*Acinetobacter baumannii* ACICU, complete genome.	Gammaproteobacteria	predicted 3-hydroxylacyl-(acyl carrier protein) dehydratase	ACC55844	100.0

Input Sequence	fig|469.3674.peg.2500 hypothetical protein [*Acinetobacter* IRMCBCU95U | 469.3674]						
	PROJECT ID	ACCESSION ID	ORGANISMS	CLASS	PROTEIN FUNCTION	PROTEIN ID	%IDENTITY
**Matched Family**	28921	XXX	*Acinetobacter baumannii* AYE	Gammaproteobacteria	XXX		100.0

Input Sequence	fig|469.3674.peg.5311 hypothetical protein [*Acinetobacter* IRMCBCU95U | 469.3674]						
	PROJECT ID	ACCESSION ID	ORGANISMS	CLASS	PROTEIN FUNCTION	PROTEIN ID	%IDENTITY

**Matched Family**	13001	XXX	Proteus mirabilis HI4320	Gammaproteobacteria	XXX		100.0

**Input Sequence**	fig|469.3674.peg.4941 hypothetical protein [*Acinetobacter* IRMCBCU95U | 469.3674]						
	PROJECT ID	ACCESSION ID	ORGANISMS	CLASS	PROTEIN FUNCTION	PROTEIN ID	%IDENTITY
**Matched Family**	17477	CP000521	*Acinetobacter baumannii* ATCC 17978, complete genome.	Gammaproteobacteria	putative signal peptide	ABO12852	100.0

**Input Sequence**	fig|469.3674.peg.6231 hypothetical protein [*Acinetobacter* IRMCBCU95U | 469.3674]						
	PROJECT ID	ACCESSION ID	ORGANISMS	CLASS	PROTEIN FUNCTION	PROTEIN ID	%IDENTITY
**Matched Family**	13001	XXX	Proteus mirabilis HI4320	Gammaproteobacteria	XXX		100.0

**Input Sequence**	fig|469.3674.peg.3918 Protein GlcG [*Acinetobacter* IRMCBCU95U | 469.3674]						
	PROJECT ID	ACCESSION ID	ORGANISMS	CLASS	PROTEIN FUNCTION	PROTEIN ID	%IDENTITY
**Matched Family**	17477	CP000521	*Acinetobacter baumannii* ATCC 17978, complete genome.	Gammaproteobacteria	hypothetical protein	ABO12369	100.0

Input Sequence	fig|469.3674.peg.6075 hypothetical protein [*Acinetobacter* IRMCBCU95U | 469.3674]						
	PROJECT ID	ACCESSION ID	ORGANISMS	CLASS	PROTEIN FUNCTION	PROTEIN ID	%IDENTITY
**Matched Family**	17827	CP000863	*Acinetobacter baumannii* ACICU, complete genome.	Gammaproteobacteria	conserved protein	ACC58635	100.0

Input Sequence	fig|469.3674.peg.6379 Transcriptional regulator, AsnC family [*Acinetobacter* IRMCBCU95U | 469.3674]						
	PROJECT ID	ACCESSION ID	ORGANISMS	CLASS	PROTEIN FUNCTION	PROTEIN ID	%IDENTITY
**Matched Family**	21111	CP001182	*Acinetobacter baumannii* AB0057, complete genome.	Gammaproteobacteria	transcriptional regulator, AsnC/Lrp family	ACJ43108	100.0

Input Sequence	fig|469.3674.peg.3266 Bis(5'-nucleosyl)-tetraphosphatase (asymmetrical) (EC 3.6.1.17) [*Acinetobacter* IRMCBCU95U | 469.3674]						
	PROJECT ID	ACCESSION ID	ORGANISMS	CLASS	PROTEIN FUNCTION	PROTEIN ID	%IDENTITY
**Matched Family**	13001	XXX	Proteus mirabilis HI4320	Gammaproteobacteria	XXX		100.0

Input Sequence	fig|469.3674.peg.7361 hypothetical protein [*Acinetobacter* IRMCBCU95U | 469.3674]						
	PROJECT ID	ACCESSION ID	ORGANISMS	CLASS	PROTEIN FUNCTION	PROTEIN ID	%IDENTITY
**Matched Family**	17827	CP000863	*Acinetobacter baumannii* ACICU,	Gammaproteobacteria	hypothetical protein	ACC56358	100.0

			complete genome.				

Input Sequence	fig|469.3674.peg.5037 FIG00350424: hypothetical protein [*Acinetobacter* IRMCBCU95U | 469.3674]						
	PROJECT ID	ACCESSION ID	ORGANISMS	CLASS	PROTEIN FUNCTION	PROTEIN ID	%IDENTITY
**Matched Family**	17827	CP000863	*Acinetobacter baumannii* ACICU, complete genome.	Gammaproteobacteria	hypothetical protein	ACC57997	100.0

Input Sequence	fig|469.3674.peg.6844 FIG00350926: hypothetical protein [*Acinetobacter* IRMCBCU95U | 469.3674]						
	PROJECT ID	ACCESSION ID	ORGANISMS	CLASS	PROTEIN FUNCTION	PROTEIN ID	%IDENTITY
**Matched Family**	17827	CP000863	*Acinetobacter baumannii* ACICU, complete genome.	Gammaproteobacteria	hypothetical protein	ACC55741	100.0

Input Sequence	fig|469.3674.peg.1854 FIG00351400: hypothetical protein [*Acinetobacter* IRMCBCU95U | 469.3674]						
	PROJECT ID	ACCESSION ID	ORGANISMS	CLASS	PROTEIN FUNCTION	PROTEIN ID	%IDENTITY
**Matched Family**	13001	XXX	Proteus mirabilis HI4320	Gammaproteobacteria	XXX		100.0

Input Sequence	fig|469.3674.peg.6033 Nudix hydrolase family protein PA3470 [*Acinetobacter* IRMCBCU95U | 469.3674]						
	PROJECT	ACCESSION	ORGANISMS	CLASS	PROTEIN FUNCTION	PROTEIN	%IDENTITY

	ID	ID				ID	
**Matched Family**	17827	CP000863	*Acinetobacter baumannii* ACICU, complete genome.	Gammaproteobacteria	NTP pyrophosphohydrolase including oxidative damage repair enzyme	ACC58606	100.0

**Input Sequence**	fig|469.3674.peg.2363 Transcriptional regulator, MerR family [*Acinetobacter* IRMCBCU95U | 469.3674]						
	PROJECT ID	ACCESSION ID	ORGANISMS	CLASS	PROTEIN FUNCTION	PROTEIN ID	%IDENTITY
**Matched Family**	13001	XXX	Proteus mirabilis HI4320	Gammaproteobacteria	XXX		100.0

**Input Sequence**	fig|469.3674.peg.1058 Ribosomal silencing factor RsfA [*Acinetobacter* IRMCBCU95U | 469.3674]						
	PROJECT ID	ACCESSION ID	ORGANISMS	CLASS	PROTEIN FUNCTION	PROTEIN ID	%IDENTITY
**Matched Family**	17827	CP000863	*Acinetobacter baumannii* ACICU, complete genome.	Gammaproteobacteria	uncharacterized plant Iojap protein	ACC55887	100.0

**Input Sequence**	fig|469.3674.peg.2725 hypothetical protein [*Acinetobacter* IRMCBCU95U | 469.3674]						
	PROJECT ID	ACCESSION ID	ORGANISMS	CLASS	PROTEIN FUNCTION	PROTEIN ID	%IDENTITY
**Matched Family**	17827	CP000863	*Acinetobacter baumannii* ACICU, complete genome.	Gammaproteobacteria	hypothetical protein	ACC56605	100.0

**Input Sequence**	fig|469.3674.peg.3423 FIG00351880: hypothetical protein [*Acinetobacter* IRMCBCU95U | 469.3674]						
	PROJECT ID	ACCESSION ID	ORGANISMS	CLASS	PROTEIN FUNCTION	PROTEIN ID	%IDENTITY
**Matched Family**	17827	CP000863	*Acinetobacter baumannii* ACICU, complete genome.	Gammaproteobacteria	hypothetical protein	ACC57104	100.0

**Input Sequence**	fig|469.3674.peg.7399 hypothetical protein [*Acinetobacter* IRMCBCU95U | 469.3674]						
	PROJECT ID	ACCESSION ID	ORGANISMS	CLASS	PROTEIN FUNCTION	PROTEIN ID	%IDENTITY
**Matched Family**	17827	CP000863	*Acinetobacter baumannii* ACICU, complete genome.	Gammaproteobacteria	hypothetical protein	ACC56375	100.0

**Input Sequence**	fig|469.3674.peg.7363 hypothetical protein [*Acinetobacter* IRMCBCU95U | 469.3674]						
	PROJECT ID	ACCESSION ID	ORGANISMS	CLASS	PROTEIN FUNCTION	PROTEIN ID	%IDENTITY
**Matched Family**	17827	CP000863	*Acinetobacter baumannii* ACICU, complete genome.	Gammaproteobacteria	hypothetical protein	ACC56360	100.0

**Input Sequence**	fig|469.3674.peg.5187 hypothetical protein [*Acinetobacter* IRMCBCU95U | 469.3674]						
	PROJECT ID	ACCESSION ID	ORGANISMS	CLASS	PROTEIN FUNCTION	PROTEIN ID	%IDENTITY
**Matched**	17477	CP000521	*Acinetobacter*	Gammaproteobacteria	hypothetical protein	ABO13017	100.0

Family			*baumannii* ATCC 17978, complete genome.				

Input Sequence	fig|469.3674.peg.4723 hypothetical protein [*Acinetobacter* IRMCBCU95U | 469.3674]						
	PROJECT ID	ACCESSION ID	ORGANISMS	CLASS	PROTEIN FUNCTION	PROTEIN ID	%IDENTITY
Matched Family	21111	CP001182	*Acinetobacter baumannii* AB0057, complete genome.	Gammaproteobacteria	conserved hypothetical protein	ACJ42799	100.0

Input Sequence	fig|469.3674.peg.6663 hypothetical protein [*Acinetobacter* IRMCBCU95U | 469.3674]						
	PROJECT ID	ACCESSION ID	ORGANISMS	CLASS	PROTEIN FUNCTION	PROTEIN ID	%IDENTITY
Matched Family	28921	XXX	*Acinetobacter baumannii* AYE	Gammaproteobacteria	XXX		100.0

Input Sequence	fig|469.3674.peg.2371 FIG00351276: hypothetical protein [*Acinetobacter* IRMCBCU95U | 469.3674]						
	PROJECT ID	ACCESSION ID	ORGANISMS	CLASS	PROTEIN FUNCTION	PROTEIN ID	%IDENTITY
Matched Family	17827	CP000863	*Acinetobacter baumannii* ACICU, complete genome.	Gammaproteobacteria	hypothetical protein	ACC56399	100.0

Input	fig|469.3674.peg.4706 FIG00351760: hypothetical protein [*Acinetobacter* IRMCBCU95U | 469.3674]

Sequence							
	PROJECT ID	ACCESSION ID	ORGANISMS	CLASS	PROTEIN FUNCTION	PROTEIN ID	%IDENTITY
**Matched Family**	21111	CP001182	*Acinetobacter baumannii* AB0057, complete genome.	Gammaproteobacteria	lipoprotein, putative	ACJ41789	100.0

**Input Sequence**	fig|469.3674.peg.5022 hypothetical protein [*Acinetobacter* IRMCBCU95U | 469.3674]						
	PROJECT ID	ACCESSION ID	ORGANISMS	CLASS	PROTEIN FUNCTION	PROTEIN ID	%IDENTITY
**Matched Family**	17477	CP000521	*Acinetobacter baumannii* ATCC 17978, complete genome.	Gammaproteobacteria	hypothetical protein	ABO12887	100.0

**Input Sequence**	fig|469.3674.peg.6247 hypothetical protein [*Acinetobacter* IRMCBCU95U | 469.3674]						
	PROJECT ID	ACCESSION ID	ORGANISMS	CLASS	PROTEIN FUNCTION	PROTEIN ID	%IDENTITY
**Matched Family**	13001	XXX	Proteus mirabilis HI4320	Gammaproteobacteria	XXX		100.0

**Input Sequence**	fig|469.3674.peg.4161 hypothetical protein [*Acinetobacter* IRMCBCU95U | 469.3674]						
	PROJECT ID	ACCESSION ID	ORGANISMS	CLASS	PROTEIN FUNCTION	PROTEIN ID	%IDENTITY
**Matched Family**	17827	CP000863	*Acinetobacter baumannii* ACICU,	Gammaproteobacteria	hypothetical protein	ACC57586	100.0

			complete genome.				

**Input Sequence**	fig|469.3674.peg.3067 General secretion pathway protein I [*Acinetobacter* IRMCBCU95U | 469.3674]						
	PROJECT ID	ACCESSION ID	ORGANISMS	CLASS	PROTEIN FUNCTION	PROTEIN ID	%IDENTITY
**Matched Family**	17827	CP000863	*Acinetobacter baumannii* ACICU, complete genome.	Gammaproteobacteria	general secretion pathway protein I precursor (PilD-dependent protein pddC)	ACC56922	100.0

**Input Sequence**	fig|469.3674.peg.7357 hypothetical protein [*Acinetobacter* IRMCBCU95U | 469.3674]						
	PROJECT ID	ACCESSION ID	ORGANISMS	CLASS	PROTEIN FUNCTION	PROTEIN ID	%IDENTITY
**Matched Family**	17827	CP000863	*Acinetobacter baumannii* ACICU, complete genome.	Gammaproteobacteria	hypothetical protein	ACC56354	100.0

**Input Sequence**	fig|469.3674.peg.4283 Dihydroneopterin aldolase (EC 4.1.2.25) [*Acinetobacter* IRMCBCU95U | 469.3674]						
	PROJECT ID	ACCESSION ID	ORGANISMS	CLASS	PROTEIN FUNCTION	PROTEIN ID	%IDENTITY
**Matched Family**	13001	XXX	Proteus mirabilis HI4320	Gammaproteobacteria	XXX		100.0

**Input Sequence**	fig|469.3674.peg.5790 hypothetical protein [*Acinetobacter* IRMCBCU95U | 469.3674]						
	PROJECT	ACCESSION	ORGANISMS	CLASS	PROTEIN FUNCTION	PROTEIN	%IDENTITY

	PROJECT ID	ACCESSION ID	ORGANISMS	CLASS	PROTEIN FUNCTION	PROTEIN ID	%IDENTITY
**Matched Family**	17827	CP000863	*Acinetobacter baumannii* ACICU, complete genome.	Gammaproteobacteria	hypothetical protein	ACC58444	100.0

**Input Sequence**	fig|469.3674.peg.626 hypothetical protein [*Acinetobacter* IRMCBCU95U | 469.3674]						
	PROJECT ID	ACCESSION ID	ORGANISMS	CLASS	PROTEIN FUNCTION	PROTEIN ID	%IDENTITY
**Matched Family**	13001	XXX	Proteus mirabilis HI4320	Gammaproteobacteria	XXX		100.0

**Input Sequence**	fig|469.3674.peg.5882 Antiholin-like protein LrgA [*Acinetobacter* IRMCBCU95U | 469.3674]						
	PROJECT ID	ACCESSION ID	ORGANISMS	CLASS	PROTEIN FUNCTION	PROTEIN ID	%IDENTITY
**Matched Family**	13001	XXX	Proteus mirabilis HI4320	Gammaproteobacteria	XXX		100.0

**Input Sequence**	fig|469.3674.peg.3271 hypothetical protein [*Acinetobacter* IRMCBCU95U | 469.3674]						
	PROJECT ID	ACCESSION ID	ORGANISMS	CLASS	PROTEIN FUNCTION	PROTEIN ID	%IDENTITY
**Matched Family**	17827	CP000863	*Acinetobacter baumannii* ACICU, complete genome.	Gammaproteobacteria	hypothetical protein	ACC57024	100.0

**Input**	fig|469.3674.peg.4170 FIG00350929: hypothetical protein [*Acinetobacter* IRMCBCU95U | 469.3674]

Sequence							
	PROJECT ID	ACCESSION ID	ORGANISMS	CLASS	PROTEIN FUNCTION	PROTEIN ID	%IDENTITY
**Matched Family**	28921	XXX	*Acinetobacter baumannii* AYE	Gammaproteobacteria	XXX		100.0

**Input Sequence**	fig|469.3674.peg.3088 hypothetical protein [*Acinetobacter* IRMCBCU95U | 469.3674]						
	PROJECT ID	ACCESSION ID	ORGANISMS	CLASS	PROTEIN FUNCTION	PROTEIN ID	%IDENTITY
**Matched Family**	17827	CP000863	*Acinetobacter baumannii* ACICU, complete genome.	Gammaproteobacteria	putative membrane protein	ACC56933	100.0

**Input Sequence**	fig|469.3674.peg.2960 hypothetical protein [*Acinetobacter* IRMCBCU95U | 469.3674]						
	PROJECT ID	ACCESSION ID	ORGANISMS	CLASS	PROTEIN FUNCTION	PROTEIN ID	%IDENTITY
**Matched Family**	17827	CP000864	*Acinetobacter baumannii* ACICU plasmid pACICU1, complete sequence.	Gammaproteobacteria	hypothetical protein	ACC59007	100.0

**Input Sequence**	fig|469.3674.peg.3135 hypothetical protein [*Acinetobacter* IRMCBCU95U | 469.3674]						
	PROJECT ID	ACCESSION ID	ORGANISMS	CLASS	PROTEIN FUNCTION	PROTEIN ID	%IDENTITY
**Matched Family**	13001	XXX	Proteus mirabilis HI4320	Gammaproteobacteria	XXX		100.0

**Input Sequence**	fig|469.3674.peg.5315 FIG00350883: hypothetical protein [*Acinetobacter* IRMCBCU95U | 469.3674]						
	PROJECT ID	ACCESSION ID	ORGANISMS	CLASS	PROTEIN FUNCTION	PROTEIN ID	%IDENTITY
**Matched Family**	17827	CP000863	*Acinetobacter baumannii* ACICU, complete genome.	Gammaproteobacteria	hypothetical protein	ACC58233	100.0

**Input Sequence**	fig|469.3674.peg.4462 hypothetical protein [*Acinetobacter* IRMCBCU95U | 469.3674]						
	PROJECT ID	ACCESSION ID	ORGANISMS	CLASS	PROTEIN FUNCTION	PROTEIN ID	%IDENTITY
**Matched Family**	17827	CP000863	*Acinetobacter baumannii* ACICU, complete genome.	Gammaproteobacteria	uncharacterized conserved protein	ACC57743	100.0

**Input Sequence**	fig|469.3674.peg.6593 HIT family hydrolase [*Acinetobacter* IRMCBCU95U | 469.3674]						
	PROJECT ID	ACCESSION ID	ORGANISMS	CLASS	PROTEIN FUNCTION	PROTEIN ID	%IDENTITY
**Matched Family**	17477	CP000521	*Acinetobacter baumannii* ATCC 17978, complete genome.	Gammaproteobacteria	putative histidine triad family protein	ABO13820	100.0

**Input Sequence**	fig|469.3674.peg.7401 hypothetical protein [*Acinetobacter* IRMCBCU95U | 469.3674]						
	PROJECT	ACCESSION	ORGANISMS	CLASS	PROTEIN FUNCTION	PROTEIN	%IDENTITY

	PROJECT ID	ACCESSION ID	ORGANISMS	CLASS	PROTEIN FUNCTION	PROTEIN ID	%IDENTITY
**Matched Family**	17827	CP000863	*Acinetobacter baumannii* ACICU, complete genome.	Gammaproteobacteria	hypothetical protein	ACC56377	100.0

**Input Sequence**	fig|469.3674.peg.1785 putative membrane protein [*Acinetobacter* IRMCBCU95U | 469.3674]						
	PROJECT ID	ACCESSION ID	ORGANISMS	CLASS	PROTEIN FUNCTION	PROTEIN ID	%IDENTITY
**Matched Family**	13001	XXX	Proteus mirabilis HI4320	Gammaproteobacteria	XXX		100.0

**Input Sequence**	fig|469.3674.peg.6779 hypothetical protein [*Acinetobacter* IRMCBCU95U | 469.3674]						
	PROJECT ID	ACCESSION ID	ORGANISMS	CLASS	PROTEIN FUNCTION	PROTEIN ID	%IDENTITY
**Matched Family**	17827	CP000863	*Acinetobacter baumannii* ACICU, complete genome.	Gammaproteobacteria	hypothetical protein	ACC55781	100.0

**Input Sequence**	fig|469.3674.peg.4564 Protein co-occuring with molybdenum cofactor biosynthesis protein B [*Acinetobacter* IRMCBCU95U | 469.3674]						
	PROJECT ID	ACCESSION ID	ORGANISMS	CLASS	PROTEIN FUNCTION	PROTEIN ID	%IDENTITY
**Matched Family**	17827	CP000863	*Acinetobacter baumannii* ACICU, complete genome.	Gammaproteobacteria	uncharacterized conserved protein	ACC57800	100.0

Input Sequence	fig|469.3674.peg.4245 hypothetical protein [*Acinetobacter* IRMCBCU95U | 469.3674]						
	PROJECT ID	ACCESSION ID	ORGANISMS	CLASS	PROTEIN FUNCTION	PROTEIN ID	%IDENTITY
**Matched Family**	17827	CP000863	*Acinetobacter baumannii* ACICU, complete genome.	Gammaproteobacteria	hypothetical protein	ACC57641	100.0

Input Sequence	fig|469.3674.peg.6778 hypothetical protein [*Acinetobacter* IRMCBCU95U | 469.3674]						
	PROJECT ID	ACCESSION ID	ORGANISMS	CLASS	PROTEIN FUNCTION	PROTEIN ID	%IDENTITY
**Matched Family**	17477	CP000521	*Acinetobacter baumannii* ATCC 17978, complete genome.	Gammaproteobacteria	putative membrane protein	ABO10914	100.0

Input Sequence	fig|469.3674.peg.2024 hypothetical protein [*Acinetobacter* IRMCBCU95U | 469.3674]						
	PROJECT ID	ACCESSION ID	ORGANISMS	CLASS	PROTEIN FUNCTION	PROTEIN ID	%IDENTITY
**Matched Family**	17827	CP000863	*Acinetobacter baumannii* ACICU, complete genome.	Gammaproteobacteria	hypothetical protein	ACC55618	100.0

Input Sequence	fig|469.3674.peg.5720 putative quaternary ammonium compound-resistance protein qacE [*Acinetobacter* IRMCBCU95U | 469.3674]						
	PROJECT ID	ACCESSION ID	ORGANISMS	CLASS	PROTEIN FUNCTION	PROTEIN ID	%IDENTITY

**Matched Family**	17827	CP000863	*Acinetobacter baumannii* ACICU, complete genome.	Gammaproteobacteria	quaternary ammonium compound-resistance protein	ACC58405	100.0

**Input Sequence**	fig|469.3674.peg.1304 FIG00349934: hypothetical protein [*Acinetobacter* IRMCBCU95U | 469.3674]						
	PROJECT ID	ACCESSION ID	ORGANISMS	CLASS	PROTEIN FUNCTION	PROTEIN ID	%IDENTITY
**Matched Family**	17477	CP000521	*Acinetobacter baumannii* ATCC 17978, complete genome.	Gammaproteobacteria	hypothetical protein	ABO11190	100.0

**Input Sequence**	fig|469.3674.peg.1144 Ribosome hibernation promoting factor Hpf [*Acinetobacter* IRMCBCU95U | 469.3674]						
	PROJECT ID	ACCESSION ID	ORGANISMS	CLASS	PROTEIN FUNCTION	PROTEIN ID	%IDENTITY
**Matched Family**	13001	XXX	Proteus mirabilis HI4320	Gammaproteobacteria	XXX		100.0

**Input Sequence**	fig|469.3674.peg.3820 Cell division protein DivIC (FtsB), stabilizes FtsL against RasP cleavage [*Acinetobacter* IRMCBCU95U | 469.3674]						
	PROJECT ID	ACCESSION ID	ORGANISMS	CLASS	PROTEIN FUNCTION	PROTEIN ID	%IDENTITY
**Matched Family**	21111	CP001182	*Acinetobacter baumannii* AB0057, complete genome.	Gammaproteobacteria	septum formation initiator	ACJ41601	100.0

Input Sequence	fig|469.3674.peg.2690 hypothetical protein [*Acinetobacter* IRMCBCU95U | 469.3674]						
	PROJECT ID	ACCESSION ID	ORGANISMS	CLASS	PROTEIN FUNCTION	PROTEIN ID	%IDENTITY
**Matched Family**	17827	CP000863	*Acinetobacter baumannii* ACICU, complete genome.	Gammaproteobacteria	hypothetical protein	ACC56586	100.0

Input Sequence	fig|469.3674.peg.2502 FIG00350662: hypothetical protein [*Acinetobacter* IRMCBCU95U | 469.3674]						
	PROJECT ID	ACCESSION ID	ORGANISMS	CLASS	PROTEIN FUNCTION	PROTEIN ID	%IDENTITY
**Matched Family**	17827	CP000863	*Acinetobacter baumannii* ACICU, complete genome.	Gammaproteobacteria	hypothetical protein	ACC56481	100.0

Input Sequence	fig|469.3674.peg.6215 hypothetical protein [*Acinetobacter* IRMCBCU95U | 469.3674]						
	PROJECT ID	ACCESSION ID	ORGANISMS	CLASS	PROTEIN FUNCTION	PROTEIN ID	%IDENTITY
**Matched Family**	13001	XXX	Proteus mirabilis HI4320	Gammaproteobacteria	XXX		100.0

Input Sequence	fig|469.3674.peg.5855 Rhodanese-like domain protein [*Acinetobacter* IRMCBCU95U | 469.3674]						
	PROJECT ID	ACCESSION ID	ORGANISMS	CLASS	PROTEIN FUNCTION	PROTEIN ID	%IDENTITY
**Matched Family**	13001	XXX	Proteus mirabilis HI4320	Gammaproteobacteria	XXX		100.0

Input Sequence	fig|469.3674.peg.3217 hypothetical protein [*Acinetobacter* IRMCBCU95U | 469.3674]					
	PROJECT ID	ACCESSION ID	ORGANISMS	CLASS	PROTEIN FUNCTION	PROTEIN ID	%IDENTITY
**Matched Family**	17827	CP000863	*Acinetobacter baumannii* ACICU, complete genome.	Gammaproteobacteria	hypothetical protein	ACC56994	100.0

Input Sequence	fig|469.3674.peg.1788 hypothetical protein [*Acinetobacter* IRMCBCU95U | 469.3674]						
	PROJECT ID	ACCESSION ID	ORGANISMS	CLASS	PROTEIN FUNCTION	PROTEIN ID	%IDENTITY
**Matched Family**	17827	CP000863	*Acinetobacter baumannii* ACICU, complete genome.	Gammaproteobacteria	hypothetical protein	ACC56830	100.0

Input Sequence	fig|469.3674.peg.6205 Cell division protein FtsL [*Acinetobacter* IRMCBCU95U | 469.3674]						
	PROJECT ID	ACCESSION ID	ORGANISMS	CLASS	PROTEIN FUNCTION	PROTEIN ID	%IDENTITY
**Matched Family**	13001	XXX	Proteus mirabilis HI4320	Gammaproteobacteria	XXX		100.0

Input Sequence	fig|469.3674.peg.5550 hypothetical protein [*Acinetobacter* IRMCBCU95U | 469.3674]						
	PROJECT ID	ACCESSION ID	ORGANISMS	CLASS	PROTEIN FUNCTION	PROTEIN ID	%IDENTITY

**Matched Family**	21111	CP001182	*Acinetobacter baumannii* AB0057, complete genome.	Gammaproteobacteria	hypothetical protein	ACJ42067	100.0

**Input Sequence**	fig|469.3674.peg.1020 FIG00349950: hypothetical protein [*Acinetobacter* IRMCBCU95U | 469.3674]						
	PROJECT ID	ACCESSION ID	ORGANISMS	CLASS	PROTEIN FUNCTION	PROTEIN ID	%IDENTITY
**Matched Family**	17827	CP000863	*Acinetobacter baumannii* ACICU, complete genome.	Gammaproteobacteria	hypothetical protein	ACC55862	100.0

**Input Sequence**	fig|469.3674.peg.6934 hypothetical protein [*Acinetobacter* IRMCBCU95U | 469.3674]						
	PROJECT ID	ACCESSION ID	ORGANISMS	CLASS	PROTEIN FUNCTION	PROTEIN ID	%IDENTITY
**Matched Family**	13001	XXX	Proteus mirabilis HI4320	Gammaproteobacteria	XXX		100.0

**Input Sequence**	fig|469.3674.peg.2588 hypothetical protein [*Acinetobacter* IRMCBCU95U | 469.3674]						
	PROJECT ID	ACCESSION ID	ORGANISMS	CLASS	PROTEIN FUNCTION	PROTEIN ID	%IDENTITY
**Matched Family**	17477	CP000521	*Acinetobacter baumannii* ATCC 17978, complete genome.	Gammaproteobacteria	hypothetical protein	ABS90059	100.0

Input Sequence	fig|469.3674.peg.3903 FIG00351379: hypothetical protein [*Acinetobacter* IRMCBCU95U | 469.3674]						
	PROJECT ID	ACCESSION ID	ORGANISMS	CLASS	PROTEIN FUNCTION	PROTEIN ID	%IDENTITY
**Matched Family**	13001	XXX	Proteus mirabilis HI4320	Gammaproteobacteria	XXX		100.0

Input Sequence	fig|469.3674.peg.1983 Putative DNA-binding protein [*Acinetobacter* IRMCBCU95U | 469.3674]						
	PROJECT ID	ACCESSION ID	ORGANISMS	CLASS	PROTEIN FUNCTION	PROTEIN ID	%IDENTITY
**Matched Family**	13001	XXX	Proteus mirabilis HI4320	Gammaproteobacteria	XXX		100.0

Input Sequence	fig|469.3674.peg.6651 hypothetical protein [*Acinetobacter* IRMCBCU95U | 469.3674]						
	PROJECT ID	ACCESSION ID	ORGANISMS	CLASS	PROTEIN FUNCTION	PROTEIN ID	%IDENTITY
**Matched Family**	21111	CP001182	*Acinetobacter baumannii* AB0057, complete genome.	Gammaproteobacteria	lipoprotein, putative	ACJ39485	100.0

Input Sequence	fig|469.3674.peg.3912 hypothetical protein [*Acinetobacter* IRMCBCU95U | 469.3674]						
	PROJECT ID	ACCESSION ID	ORGANISMS	CLASS	PROTEIN FUNCTION	PROTEIN ID	%IDENTITY
**Matched Family**	17827	CP000863	*Acinetobacter baumannii* ACICU, complete genome.	Gammaproteobacteria	hypothetical protein	ACC57365	100.0

**Input Sequence**	fig|469.3674.peg.725 hypothetical protein [*Acinetobacter* IRMCBCU95U | 469.3674]						
	PROJECT ID	ACCESSION ID	ORGANISMS	CLASS	PROTEIN FUNCTION	PROTEIN ID	%IDENTITY
**Matched Family**	17477	CP000521	*Acinetobacter baumannii* ATCC 17978, complete genome.	Gammaproteobacteria	putative signal peptide	ABO10527	100.0

**Input Sequence**	fig|469.3674.peg.5186 hypothetical protein [*Acinetobacter* IRMCBCU95U | 469.3674]						
	PROJECT ID	ACCESSION ID	ORGANISMS	CLASS	PROTEIN FUNCTION	PROTEIN ID	%IDENTITY
**Matched Family**	17827	CP000863	*Acinetobacter baumannii* ACICU, complete genome.	Gammaproteobacteria	hypothetical protein	ACC58154	100.0

**Input Sequence**	fig|469.3674.peg.7290 FIG00350276: hypothetical protein [*Acinetobacter* IRMCBCU95U | 469.3674]						
	PROJECT ID	ACCESSION ID	ORGANISMS	CLASS	PROTEIN FUNCTION	PROTEIN ID	%IDENTITY
**Matched Family**	17477	CP000521	*Acinetobacter baumannii* ATCC 17978, complete genome.	Gammaproteobacteria	hypothetical protein	ABS90016	100.0

**Input Sequence**	fig|469.3674.peg.2670 hypothetical protein [*Acinetobacter* IRMCBCU95U | 469.3674]

	PROJECT ID	ACCESSION ID	ORGANISMS	CLASS	PROTEIN FUNCTION	PROTEIN ID	%IDENTITY
**Matched Family**	17827	CP000863	*Acinetobacter baumannii* ACICU, complete genome.	Gammaproteobacteria	hypothetical protein	ACC56576	100.0

**Input Sequence**	fig|469.3674.peg.6423 Zn-ribbon-containing, possibly RNA-binding protein and truncated derivatives [*Acinetobacter* IRMCBCU95U | 469.3674]						
	PROJECT ID	ACCESSION ID	ORGANISMS	CLASS	PROTEIN FUNCTION	PROTEIN ID	%IDENTITY
**Matched Family**	21111	CP001182	*Acinetobacter baumannii* AB0057, complete genome.	Gammaproteobacteria	conserved hypothetical protein	ACJ43131	100.0

**Input Sequence**	fig|469.3674.peg.5316 hypothetical protein [*Acinetobacter* IRMCBCU95U | 469.3674]						
	PROJECT ID	ACCESSION ID	ORGANISMS	CLASS	PROTEIN FUNCTION	PROTEIN ID	%IDENTITY
**Matched Family**	13001	XXX	Proteus mirabilis HI4320	Gammaproteobacteria	XXX		100.0

**Input Sequence**	fig|469.3674.peg.7374 hypothetical protein [*Acinetobacter* IRMCBCU95U | 469.3674]						
	PROJECT ID	ACCESSION ID	ORGANISMS	CLASS	PROTEIN FUNCTION	PROTEIN ID	%IDENTITY
**Matched Family**	28921	XXX	*Acinetobacter baumannii* AYE	Gammaproteobacteria	XXX		100.0

Input Sequence	fig|469.3674.peg.3520 Transcriptional regulator [*Acinetobacter* IRMCBCU95U | 469.3674]						
	PROJECT ID	ACCESSION ID	ORGANISMS	CLASS	PROTEIN FUNCTION	PROTEIN ID	%IDENTITY
Matched Family	17827	CP000863	*Acinetobacter baumannii* ACICU, complete genome.	Gammaproteobacteria	predicted transcriptional regulator	ACC57152	100.0

Input Sequence	fig|469.3674.peg.4022 tRNA 2-thiouridine synthesis protein TusE [*Acinetobacter* IRMCBCU95U | 469.3674]						
	PROJECT ID	ACCESSION ID	ORGANISMS	CLASS	PROTEIN FUNCTION	PROTEIN ID	%IDENTITY
Matched Family	13001	XXX	Proteus mirabilis HI4320	Gammaproteobacteria	XXX		100.0

Input Sequence	fig|469.3674.peg.1429 LSU ribosomal protein L25p [*Acinetobacter* IRMCBCU95U | 469.3674]						
	PROJECT ID	ACCESSION ID	ORGANISMS	CLASS	PROTEIN FUNCTION	PROTEIN ID	%IDENTITY
Matched Family	17827	CP000863	*Acinetobacter baumannii* ACICU, complete genome.	Gammaproteobacteria	putative 50S ribosomal protein L25	ACC56098	100.0

Input Sequence	fig|469.3674.peg.1082 Phosphocarrier protein, nitrogen regulation associated [*Acinetobacter* IRMCBCU95U | 469.3674]						
	PROJECT ID	ACCESSION ID	ORGANISMS	CLASS	PROTEIN FUNCTION	PROTEIN ID	%IDENTITY
Matched Family	13001	XXX	Proteus mirabilis HI4320	Gammaproteobacteria	XXX		100.0

**Input Sequence**	fig|469.3674.peg.4230 Antitoxin DinJ (binds YafQ toxin) [*Acinetobacter* IRMCBCU95U | 469.3674]						
	PROJECT ID	ACCESSION ID	ORGANISMS	CLASS	PROTEIN FUNCTION	PROTEIN ID	%IDENTITY
**Matched Family**	17477	CP000521	*Acinetobacter baumannii* ATCC 17978, complete genome.	Gammaproteobacteria	hypothetical protein	ABO12545	100.0

**Input Sequence**	fig|469.3674.peg.5403 Proposed lipoate regulatory protein YbeD [*Acinetobacter* IRMCBCU95U | 469.3674]						
	PROJECT ID	ACCESSION ID	ORGANISMS	CLASS	PROTEIN FUNCTION	PROTEIN ID	%IDENTITY
**Matched Family**	13001	XXX	Proteus mirabilis HI4320	Gammaproteobacteria	XXX		100.0

**Input Sequence**	fig|469.3674.peg.6209 hypothetical protein [*Acinetobacter* IRMCBCU95U | 469.3674]						
	PROJECT ID	ACCESSION ID	ORGANISMS	CLASS	PROTEIN FUNCTION	PROTEIN ID	%IDENTITY
**Matched Family**	17477	CP000521	*Acinetobacter baumannii* ATCC 17978, complete genome.	Gammaproteobacteria	putative peptide signal	ABO13601	100.0

**Input Sequence**	fig|469.3674.peg.1325 putative cold shock protein [*Acinetobacter* IRMCBCU95U | 469.3674]						
	PROJECT	ACCESSION	ORGANISMS	CLASS	PROTEIN FUNCTION	PROTEIN	%IDENTITY

	ID	ID				ID	
**Matched Family**	17477	CP000521	*Acinetobacter baumannii* ATCC 17978, complete genome.	Gammaproteobacteria	putative cold shock protein	ABO11206	100.0

**Input Sequence**	fig|469.3674.peg.3951 FIG00351831: hypothetical protein [*Acinetobacter* IRMCBCU95U | 469.3674]						
	PROJECT ID	ACCESSION ID	ORGANISMS	CLASS	PROTEIN FUNCTION	PROTEIN ID	%IDENTITY
**Matched Family**	17827	CP000863	*Acinetobacter baumannii* ACICU, complete genome.	Gammaproteobacteria	hypothetical protein	ACC57392	100.0

**Input Sequence**	fig|469.3674.peg.3215 hypothetical protein [*Acinetobacter* IRMCBCU95U | 469.3674]						
	PROJECT ID	ACCESSION ID	ORGANISMS	CLASS	PROTEIN FUNCTION	PROTEIN ID	%IDENTITY
**Matched Family**	17827	CP000863	*Acinetobacter baumannii* ACICU, complete genome.	Gammaproteobacteria	hypothetical protein	ACC56992	100.0

**Input Sequence**	fig|469.3674.peg.4367 DNA-binding protein Fis [*Acinetobacter* IRMCBCU95U | 469.3674]						
	PROJECT ID	ACCESSION ID	ORGANISMS	CLASS	PROTEIN FUNCTION	PROTEIN ID	%IDENTITY
**Matched Family**	12352	XXX	*Acinetobacter* sp. ADP1	Gammaproteobacteria	XXX		100.0

**Input Sequence**	fig|469.3674.peg.3472 hypothetical protein [*Acinetobacter* IRMCBCU95U | 469.3674]						
	PROJECT ID	ACCESSION ID	ORGANISMS	CLASS	PROTEIN FUNCTION	PROTEIN ID	%IDENTITY
**Matched Family**	17827	CP000863	*Acinetobacter baumannii* ACICU, complete genome.	Gammaproteobacteria	hypothetical protein	ACC57126	100.0

**Input Sequence**	fig|469.3674.peg.6036 Phospholipid ABC transporter-binding protein MlaB [*Acinetobacter* IRMCBCU95U | 469.3674]						
	PROJECT ID	ACCESSION ID	ORGANISMS	CLASS	PROTEIN FUNCTION	PROTEIN ID	%IDENTITY
**Matched Family**	13001	XXX	Proteus mirabilis HI4320	Gammaproteobacteria	XXX		100.0

**Input Sequence**	fig|469.3674.peg.2350 Z-ring-associated protein ZapA [*Acinetobacter* IRMCBCU95U | 469.3674]						
	PROJECT ID	ACCESSION ID	ORGANISMS	CLASS	PROTEIN FUNCTION	PROTEIN ID	%IDENTITY
**Matched Family**	17827	CP000863	*Acinetobacter baumannii* ACICU, complete genome.	Gammaproteobacteria	hypothetical protein	ACC56388	100.0

**Input Sequence**	fig|469.3674.peg.6070 hypothetical protein [*Acinetobacter* IRMCBCU95U | 469.3674]						
	PROJECT ID	ACCESSION ID	ORGANISMS	CLASS	PROTEIN FUNCTION	PROTEIN ID	%IDENTITY
**Matched Family**	17827	CP000863	*Acinetobacter baumannii* ACICU,	Gammaproteobacteria	uncharacterized protein conserved in bacteria	ACC58632	100.0

			complete genome.				

Input Sequence	fig|469.3674.peg.5742 hypothetical protein [*Acinetobacter* IRMCBCU95U | 469.3674]						
	PROJECT ID	ACCESSION ID	ORGANISMS	CLASS	PROTEIN FUNCTION	PROTEIN ID	%IDENTITY
**Matched Family**	13001	XXX	Proteus mirabilis HI4320	Gammaproteobacteria	XXX		100.0

Input Sequence	fig|469.3674.peg.219 FIG00350011: hypothetical protein [*Acinetobacter* IRMCBCU95U | 469.3674]						
	PROJECT ID	ACCESSION ID	ORGANISMS	CLASS	PROTEIN FUNCTION	PROTEIN ID	%IDENTITY
**Matched Family**	21111	CP001182	*Acinetobacter baumannii* AB0057, complete genome.	Gammaproteobacteria	hypothetical protein	ACJ40771	100.0

Input Sequence	fig|469.3674.peg.5629 FIG00351427: hypothetical protein [*Acinetobacter* IRMCBCU95U | 469.3674]						
	PROJECT ID	ACCESSION ID	ORGANISMS	CLASS	PROTEIN FUNCTION	PROTEIN ID	%IDENTITY
**Matched Family**	13001	XXX	Proteus mirabilis HI4320	Gammaproteobacteria	XXX		100.0

Input Sequence	fig|469.3674.peg.3355 FIG028220: hypothetical protein co-occurring with HEAT repeat protein [*Acinetobacter* IRMCBCU95U | 469.3674]						
	PROJECT ID	ACCESSION ID	ORGANISMS	CLASS	PROTEIN FUNCTION	PROTEIN ID	%IDENTITY

**Matched Family**	17827	CP000863	*Acinetobacter baumannii* ACICU, complete genome.	Gammaproteobacteria	uncharacterized protein conserved in bacteria	ACC57071	100.0

**Input Sequence**	fig|469.3674.peg.4162 hypothetical protein [*Acinetobacter* IRMCBCU95U | 469.3674]						
	PROJECT ID	ACCESSION ID	ORGANISMS	CLASS	PROTEIN FUNCTION	PROTEIN ID	%IDENTITY
**Matched Family**	17827	CP000863	*Acinetobacter baumannii* ACICU, complete genome.	Gammaproteobacteria	hypothetical protein	ACC57587	100.0

**Input Sequence**	fig|469.3674.peg.6803 hypothetical protein [*Acinetobacter* IRMCBCU95U | 469.3674]						
	PROJECT ID	ACCESSION ID	ORGANISMS	CLASS	PROTEIN FUNCTION	PROTEIN ID	%IDENTITY
**Matched Family**	17477	CP000521	*Acinetobacter baumannii* ATCC 17978, complete genome.	Gammaproteobacteria	hypothetical protein	ABO10900	100.0

**Input Sequence**	fig|469.3674.peg.139 FIG00350382: hypothetical protein [*Acinetobacter* IRMCBCU95U | 469.3674]						
	PROJECT ID	ACCESSION ID	ORGANISMS	CLASS	PROTEIN FUNCTION	PROTEIN ID	%IDENTITY
**Matched Family**	17827	CP000863	*Acinetobacter baumannii* ACICU, complete genome.	Gammaproteobacteria	hypothetical protein	ACC57485	100.0

**Input Sequence**	fig|469.3674.peg.218 FIG00352211: hypothetical protein [*Acinetobacter* IRMCBCU95U | 469.3674]						
	PROJECT ID	ACCESSION ID	ORGANISMS	CLASS	PROTEIN FUNCTION	PROTEIN ID	%IDENTITY
**Matched Family**	21111	CP001182	*Acinetobacter baumannii* AB0057, complete genome.	Gammaproteobacteria	conserved hypothetical protein	ACJ40770	100.0

**Input Sequence**	fig|469.3674.peg.6415 FIG00351517: hypothetical protein [*Acinetobacter* IRMCBCU95U | 469.3674]						
	PROJECT ID	ACCESSION ID	ORGANISMS	CLASS	PROTEIN FUNCTION	PROTEIN ID	%IDENTITY
**Matched Family**	17827	CP000863	*Acinetobacter baumannii* ACICU, complete genome.	Gammaproteobacteria	hypothetical protein	ACC58832	100.0

**Input Sequence**	fig|469.3674.peg.1145 YrbA protein [*Acinetobacter* IRMCBCU95U | 469.3674]						
	PROJECT ID	ACCESSION ID	ORGANISMS	CLASS	PROTEIN FUNCTION	PROTEIN ID	%IDENTITY
**Matched Family**	13001	XXX	Proteus mirabilis HI4320	Gammaproteobacteria	XXX		100.0

**Input Sequence**	fig|469.3674.peg.3290 hypothetical protein [*Acinetobacter* IRMCBCU95U | 469.3674]						
	PROJECT ID	ACCESSION ID	ORGANISMS	CLASS	PROTEIN FUNCTION	PROTEIN ID	%IDENTITY
**Matched Family**	17827	CP000863	*Acinetobacter baumannii* ACICU,	Gammaproteobacteria	hypothetical protein	ACC57033	100.0

			complete genome.				

**Input Sequence**	fig|469.3674.peg.99 FIG00350805: hypothetical protein [*Acinetobacter* IRMCBCU95U | 469.3674]						
	PROJECT ID	ACCESSION ID	ORGANISMS	CLASS	PROTEIN FUNCTION	PROTEIN ID	%IDENTITY
**Matched Family**	17827	CP000863	*Acinetobacter baumannii* ACICU, complete genome.	Gammaproteobacteria	hypothetical protein	ACC57514	100.0

**Input Sequence**	fig|469.3674.peg.4184 hypothetical protein [*Acinetobacter* IRMCBCU95U | 469.3674]						
	PROJECT ID	ACCESSION ID	ORGANISMS	CLASS	PROTEIN FUNCTION	PROTEIN ID	%IDENTITY
**Matched Family**	17827	CP000863	*Acinetobacter baumannii* ACICU, complete genome.	Gammaproteobacteria	hypothetical protein	ACC57601	100.0

**Input Sequence**	fig|469.3674.peg.7325 FIG00350569: hypothetical protein [*Acinetobacter* IRMCBCU95U | 469.3674]						
	PROJECT ID	ACCESSION ID	ORGANISMS	CLASS	PROTEIN FUNCTION	PROTEIN ID	%IDENTITY
**Matched Family**	17827	CP000863	*Acinetobacter baumannii* ACICU, complete genome.	Gammaproteobacteria	hypothetical protein	ACC56335	100.0

**Input Sequence**	fig|469.3674.peg.1946 hypothetical protein [*Acinetobacter* IRMCBCU95U | 469.3674]

	PROJECT ID	ACCESSION ID	ORGANISMS	CLASS	PROTEIN FUNCTION	PROTEIN ID	%IDENTITY
**Matched Family**	17827	CP000863	*Acinetobacter baumannii* ACICU, complete genome.	Gammaproteobacteria	hypothetical protein	ACC55579	100.0

**Input Sequence**	fig|469.3674.peg.2362 FIG00350187: hypothetical protein [*Acinetobacter* IRMCBCU95U | 469.3674]						
	PROJECT ID	ACCESSION ID	ORGANISMS	CLASS	PROTEIN FUNCTION	PROTEIN ID	%IDENTITY
**Matched Family**	13001	XXX	Proteus mirabilis HI4320	Gammaproteobacteria	XXX		100.0

**Input Sequence**	fig|469.3674.peg.4164 FIG00351023: hypothetical protein [*Acinetobacter* IRMCBCU95U | 469.3674]						
	PROJECT ID	ACCESSION ID	ORGANISMS	CLASS	PROTEIN FUNCTION	PROTEIN ID	%IDENTITY
**Matched Family**	13001	XXX	Proteus mirabilis HI4320	Gammaproteobacteria	XXX		100.0

**Input Sequence**	fig|469.3674.peg.7295 FIG00351948: hypothetical protein [*Acinetobacter* IRMCBCU95U | 469.3674]						
	PROJECT ID	ACCESSION ID	ORGANISMS	CLASS	PROTEIN FUNCTION	PROTEIN ID	%IDENTITY
**Matched Family**	17477	CP000521	*Acinetobacter baumannii* ATCC 17978, complete genome.	Gammaproteobacteria	hypothetical protein	ABS90029	100.0

Input Sequence	fig|469.3674.peg.3919 hypothetical protein [*Acinetobacter* IRMCBCU95U | 469.3674]						
	PROJECT ID	ACCESSION ID	ORGANISMS	CLASS	PROTEIN FUNCTION	PROTEIN ID	%IDENTITY
Matched Family	28921	XXX	*Acinetobacter baumannii* AYE	Gammaproteobacteria	XXX		100.0

Input Sequence	fig|469.3674.peg.1872 FIG00351277: hypothetical protein [*Acinetobacter* IRMCBCU95U | 469.3674]						
	PROJECT ID	ACCESSION ID	ORGANISMS	CLASS	PROTEIN FUNCTION	PROTEIN ID	%IDENTITY
Matched Family	13001	XXX	Proteus mirabilis HI4320	Gammaproteobacteria	XXX		100.0

Input Sequence	fig|469.3674.peg.6819 Protein SlyX [*Acinetobacter* IRMCBCU95U | 469.3674]						
	PROJECT ID	ACCESSION ID	ORGANISMS	CLASS	PROTEIN FUNCTION	PROTEIN ID	%IDENTITY
Matched Family	13001	XXX	Proteus mirabilis HI4320	Gammaproteobacteria	XXX		100.0

Input Sequence	fig|469.3674.peg.4521 Uncharacterized UPF0033 protein [*Acinetobacter* IRMCBCU95U | 469.3674]						
	PROJECT ID	ACCESSION ID	ORGANISMS	CLASS	PROTEIN FUNCTION	PROTEIN ID	%IDENTITY
Matched Family	13001	XXX	Proteus mirabilis HI4320	Gammaproteobacteria	XXX		100.0

**Input Sequence**	fig|469.3674.peg.6597 hypothetical protein [*Acinetobacter* IRMCBCU95U | 469.3674]						
	PROJECT ID	ACCESSION ID	ORGANISMS	CLASS	PROTEIN FUNCTION	PROTEIN ID	%IDENTITY
**Matched Family**	13001	XXX	Proteus mirabilis HI4320	Gammaproteobacteria	XXX		100.0

**Input Sequence**	fig|469.3674.peg.5242 Lipoprotein [*Acinetobacter* IRMCBCU95U | 469.3674]						
	PROJECT ID	ACCESSION ID	ORGANISMS	CLASS	PROTEIN FUNCTION	PROTEIN ID	%IDENTITY
**Matched Family**	13001	XXX	Proteus mirabilis HI4320	Gammaproteobacteria	XXX		100.0

**Input Sequence**	fig|469.3674.peg.3952 FIG00350507: hypothetical protein [*Acinetobacter* IRMCBCU95U | 469.3674]						
	PROJECT ID	ACCESSION ID	ORGANISMS	CLASS	PROTEIN FUNCTION	PROTEIN ID	%IDENTITY
**Matched Family**	17827	CP000863	*Acinetobacter baumannii* ACICU, complete genome.	Gammaproteobacteria	hypothetical protein	ACC57393	100.0

**Input Sequence**	fig|469.3674.peg.3280 hypothetical protein [*Acinetobacter* IRMCBCU95U | 469.3674]						
	PROJECT ID	ACCESSION ID	ORGANISMS	CLASS	PROTEIN FUNCTION	PROTEIN ID	%IDENTITY
**Matched Family**	13001	XXX	Proteus mirabilis HI4320	Gammaproteobacteria	XXX		100.0

Input Sequence	fig|469.3674.peg.3441 FIG00350577: hypothetical protein [*Acinetobacter* IRMCBCU95U | 469.3674]						
	PROJECT ID	ACCESSION ID	ORGANISMS	CLASS	PROTEIN FUNCTION	PROTEIN ID	%IDENTITY
Matched Family	17827	CP000863	*Acinetobacter baumannii* ACICU, complete genome.	Gammaproteobacteria	hypothetical protein	ACC57113	100.0

Input Sequence	fig|469.3674.peg.6364 Inner membrane protein YjcH, clustering with ActP [*Acinetobacter* IRMCBCU95U | 469.3674]						
	PROJECT ID	ACCESSION ID	ORGANISMS	CLASS	PROTEIN FUNCTION	PROTEIN ID	%IDENTITY
Matched Family	17827	CP000863	*Acinetobacter baumannii* ACICU, complete genome.	Gammaproteobacteria	predicted membrane protein	ACC58806	100.0

Input Sequence	fig|469.3674.peg.7328 FIG00350954: hypothetical protein [*Acinetobacter* IRMCBCU95U | 469.3674]						
	PROJECT ID	ACCESSION ID	ORGANISMS	CLASS	PROTEIN FUNCTION	PROTEIN ID	%IDENTITY
Matched Family	17827	CP000863	*Acinetobacter baumannii* ACICU, complete genome.	Gammaproteobacteria	hypothetical protein	ACC56338	100.0

Input Sequence	fig|469.3674.peg.3905 hypothetical protein [*Acinetobacter* IRMCBCU95U | 469.3674]						
	PROJECT ID	ACCESSION ID	ORGANISMS	CLASS	PROTEIN FUNCTION	PROTEIN ID	%IDENTITY

**Matched Family**	13001	XXX	Proteus mirabilis HI4320	Gammaproteobacteria	XXX		100.0

**Input Sequence**	fig|469.3674.peg.4468 Bsr8028 protein [*Acinetobacter* IRMCBCU95U | 469.3674]						
	PROJECT ID	ACCESSION ID	ORGANISMS	CLASS	PROTEIN FUNCTION	PROTEIN ID	%IDENTITY
**Matched Family**	17477	CP000521	*Acinetobacter baumannii* ATCC 17978, complete genome.	Gammaproteobacteria	hypothetical protein	ABO12657	100.0

**Input Sequence**	fig|469.3674.peg.1883 hypothetical protein [*Acinetobacter* IRMCBCU95U | 469.3674]						
	PROJECT ID	ACCESSION ID	ORGANISMS	CLASS	PROTEIN FUNCTION	PROTEIN ID	%IDENTITY
**Matched Family**	17477	CP000521	*Acinetobacter baumannii* ATCC 17978, complete genome.	Gammaproteobacteria	hypothetical protein	ABO11953	100.0

**Input Sequence**	fig|469.3674.peg.4364 hypothetical protein [*Acinetobacter* IRMCBCU95U | 469.3674]						
	PROJECT ID	ACCESSION ID	ORGANISMS	CLASS	PROTEIN FUNCTION	PROTEIN ID	%IDENTITY
**Matched Family**	17477	CP000521	*Acinetobacter baumannii* ATCC 17978, complete genome.	Gammaproteobacteria	putative signal peptide	ABO12610	100.0

Input Sequence	fig|469.3674.peg.6652 hypothetical protein [*Acinetobacter* IRMCBCU95U | 469.3674]						
	PROJECT ID	ACCESSION ID	ORGANISMS	CLASS	PROTEIN FUNCTION	PROTEIN ID	%IDENTITY
Matched Family	17477	CP000521	*Acinetobacter baumannii* ATCC 17978, complete genome.	Gammaproteobacteria	hypothetical protein	ABS90290	100.0

Input Sequence	fig|469.3674.peg.5962 Cytoplasmic protein, probably associated with glutathione-regulated potassium-efflux [*Acinetobacter* IRMCBCU95U | 469.3674]						
	PROJECT ID	ACCESSION ID	ORGANISMS	CLASS	PROTEIN FUNCTION	PROTEIN ID	%IDENTITY
Matched Family	13001	XXX	Proteus mirabilis HI4320	Gammaproteobacteria	XXX		100.0

Input Sequence	fig|469.3674.peg.4149 FIG00350978: hypothetical protein [*Acinetobacter* IRMCBCU95U | 469.3674]						
	PROJECT ID	ACCESSION ID	ORGANISMS	CLASS	PROTEIN FUNCTION	PROTEIN ID	%IDENTITY
Matched Family	17827	CP000863	*Acinetobacter baumannii* ACICU, complete genome.	Gammaproteobacteria	hypothetical protein	ACC57578	100.0

Input Sequence	fig|469.3674.peg.3363 FIG00352393: hypothetical protein [*Acinetobacter* IRMCBCU95U | 469.3674]						
	PROJECT ID	ACCESSION ID	ORGANISMS	CLASS	PROTEIN FUNCTION	PROTEIN ID	%IDENTITY

**Matched Family**	17827	CP000863	*Acinetobacter baumannii* ACICU, complete genome.	Gammaproteobacteria	hypothetical protein	ACC57076	100.0

**Input Sequence**	fig|469.3674.peg.2564 FIG00351239: hypothetical protein [*Acinetobacter* IRMCBCU95U | 469.3674]						
	PROJECT ID	ACCESSION ID	ORGANISMS	CLASS	PROTEIN FUNCTION	PROTEIN ID	%IDENTITY
**Matched Family**	13001	XXX	Proteus mirabilis HI4320	Gammaproteobacteria	XXX		100.0

**Input Sequence**	fig|469.3674.peg.1913 FIG00349975: hypothetical protein [*Acinetobacter* IRMCBCU95U | 469.3674]						
	PROJECT ID	ACCESSION ID	ORGANISMS	CLASS	PROTEIN FUNCTION	PROTEIN ID	%IDENTITY
**Matched Family**	17477	CP000521	*Acinetobacter baumannii* ATCC 17978, complete genome.	Gammaproteobacteria	hypothetical protein	ABO10699	100.0

**Input Sequence**	fig|469.3674.peg.4233 FIG00352022: hypothetical protein [*Acinetobacter* IRMCBCU95U | 469.3674]						
	PROJECT ID	ACCESSION ID	ORGANISMS	CLASS	PROTEIN FUNCTION	PROTEIN ID	%IDENTITY
**Matched Family**	17477	CP000521	*Acinetobacter baumannii* ATCC 17978, complete genome.	Gammaproteobacteria	hypothetical protein	ABS90211	100.0

Input Sequence	fig|469.3674.peg.5806 hypothetical protein [*Acinetobacter* IRMCBCU95U | 469.3674]						
	PROJECT ID	ACCESSION ID	ORGANISMS	CLASS	PROTEIN FUNCTION	PROTEIN ID	%IDENTITY
**Matched Family**	13001	XXX	Proteus mirabilis HI4320	Gammaproteobacteria	XXX		100.0

Input Sequence	fig|469.3674.peg.7326 FIG00350708: hypothetical protein [*Acinetobacter* IRMCBCU95U | 469.3674]						
	PROJECT ID	ACCESSION ID	ORGANISMS	CLASS	PROTEIN FUNCTION	PROTEIN ID	%IDENTITY
**Matched Family**	17827	CP000863	*Acinetobacter baumannii* ACICU, complete genome.	Gammaproteobacteria	hypothetical protein	ACC56336	100.0

Input Sequence	fig|469.3674.peg.7364 hypothetical protein [*Acinetobacter* IRMCBCU95U | 469.3674]						
	PROJECT ID	ACCESSION ID	ORGANISMS	CLASS	PROTEIN FUNCTION	PROTEIN ID	%IDENTITY
**Matched Family**	17477	CP000521	*Acinetobacter baumannii* ATCC 17978, complete genome.	Gammaproteobacteria	hypothetical protein	ABS90187	100.0

Input Sequence	fig|469.3674.peg.4717 hypothetical protein [*Acinetobacter* IRMCBCU95U | 469.3674]						
	PROJECT ID	ACCESSION ID	ORGANISMS	CLASS	PROTEIN FUNCTION	PROTEIN ID	%IDENTITY
**Matched**	17827	CP000863	*Acinetobacter*	Gammaproteobacteria	hypothetical protein	ACC56361	100.0

Family			*baumannii* ACICU, complete genome.				

Input Sequence	fig|469.3674.peg.7308 hypothetical protein [*Acinetobacter* IRMCBCU95U | 469.3674]						
	PROJECT ID	ACCESSION ID	ORGANISMS	CLASS	PROTEIN FUNCTION	PROTEIN ID	%IDENTITY
Matched Family	17827	CP000863	*Acinetobacter baumannii* ACICU, complete genome.	Gammaproteobacteria	hypothetical protein	ACC56323	100.0

Input Sequence	fig|469.3674.peg.2521 hypothetical protein [*Acinetobacter* IRMCBCU95U | 469.3674]						
	PROJECT ID	ACCESSION ID	ORGANISMS	CLASS	PROTEIN FUNCTION	PROTEIN ID	%IDENTITY
Matched Family	17477	CP000521	*Acinetobacter baumannii* ATCC 17978, complete genome.	Gammaproteobacteria	hypothetical protein	ABO11634	100.0

Input Sequence	fig|469.3674.peg.3931 FIG00350468: hypothetical protein [*Acinetobacter* IRMCBCU95U | 469.3674]						
	PROJECT ID	ACCESSION ID	ORGANISMS	CLASS	PROTEIN FUNCTION	PROTEIN ID	%IDENTITY
Matched Family	17827	CP000863	*Acinetobacter baumannii* ACICU, complete genome.	Gammaproteobacteria	hypothetical protein	ACC57379	100.0

Input Sequence	fig|469.3674.peg.1855 FIG00350416: hypothetical protein [*Acinetobacter* IRMCBCU95U | 469.3674]						
	PROJECT ID	ACCESSION ID	ORGANISMS	CLASS	PROTEIN FUNCTION	PROTEIN ID	%IDENTITY
**Matched Family**	17477	CP000521	*Acinetobacter baumannii* ATCC 17978, complete genome.	Gammaproteobacteria	hypothetical protein	ABS90102	100.0

Input Sequence	fig|469.3674.peg.5400 FIG00350425: hypothetical protein [*Acinetobacter* IRMCBCU95U | 469.3674]						
	PROJECT ID	ACCESSION ID	ORGANISMS	CLASS	PROTEIN FUNCTION	PROTEIN ID	%IDENTITY
**Matched Family**	17477	CP000521	*Acinetobacter baumannii* ATCC 17978, complete genome.	Gammaproteobacteria	hypothetical protein	ABS90255	100.0

Input Sequence	fig|469.3674.peg.515 hypothetical protein [*Acinetobacter* IRMCBCU95U | 469.3674]						
	PROJECT ID	ACCESSION ID	ORGANISMS	CLASS	PROTEIN FUNCTION	PROTEIN ID	%IDENTITY
**Matched Family**	21111	CP001182	*Acinetobacter baumannii* AB0057, complete genome.	Gammaproteobacteria	conserved hypothetical protein	ACJ39638	100.0

Input Sequence	fig|469.3674.peg.5562 hypothetical protein [*Acinetobacter* IRMCBCU95U | 469.3674]						
	PROJECT ID	ACCESSION ID	ORGANISMS	CLASS	PROTEIN FUNCTION	PROTEIN ID	%IDENTITY

**Matched Family**	21111	CP001182	*Acinetobacter baumannii* AB0057, complete genome.	Gammaproteobacteria	hypothetical protein	ACJ42080	100.0

**Input Sequence**	fig|469.3674.peg.120 FIG00353296: hypothetical protein [*Acinetobacter* IRMCBCU95U | 469.3674]						
	PROJECT ID	ACCESSION ID	ORGANISMS	CLASS	PROTEIN FUNCTION	PROTEIN ID	%IDENTITY
**Matched Family**	17827	CP000863	*Acinetobacter baumannii* ACICU, complete genome.	Gammaproteobacteria	hypothetical protein	ACC57499	100.0

**Input Sequence**	fig|469.3674.peg.3269 hypothetical protein [*Acinetobacter* IRMCBCU95U | 469.3674]						
	PROJECT ID	ACCESSION ID	ORGANISMS	CLASS	PROTEIN FUNCTION	PROTEIN ID	%IDENTITY
**Matched Family**	13001	XXX	Proteus mirabilis HI4320	Gammaproteobacteria	XXX		100.0

**Input Sequence**	fig|469.3674.peg.5485 hypothetical protein [*Acinetobacter* IRMCBCU95U | 469.3674]						
	PROJECT ID	ACCESSION ID	ORGANISMS	CLASS	PROTEIN FUNCTION	PROTEIN ID	%IDENTITY
**Matched Family**	21111	CP001182	*Acinetobacter baumannii* AB0057, complete genome.	Gammaproteobacteria	hypothetical protein	ACJ41769	100.0

**Input**	fig|469.3674.peg.5627 hypothetical protein [*Acinetobacter* IRMCBCU95U | 469.3674]

Sequence							
	PROJECT ID	ACCESSION ID	ORGANISMS	CLASS	PROTEIN FUNCTION	PROTEIN ID	%IDENTITY
**Matched Family**	13001	XXX	Proteus mirabilis HI4320	Gammaproteobacteria	XXX		100.0

**Input Sequence**	fig|469.3674.peg.2570 hypothetical protein [*Acinetobacter* IRMCBCU95U | 469.3674]						
	PROJECT ID	ACCESSION ID	ORGANISMS	CLASS	PROTEIN FUNCTION	PROTEIN ID	%IDENTITY
**Matched Family**	13001	XXX	Proteus mirabilis HI4320	Gammaproteobacteria	XXX		100.0

**Input Sequence**	fig|469.3674.peg.2565 Exodeoxyribonuclease VII small subunit (EC 3.1.11.6) [*Acinetobacter* IRMCBCU95U | 469.3674]						
	PROJECT ID	ACCESSION ID	ORGANISMS	CLASS	PROTEIN FUNCTION	PROTEIN ID	%IDENTITY
**Matched Family**	17827	CP000863	*Acinetobacter baumannii* ACICU, complete genome.	Gammaproteobacteria	hypothetical protein	ACC56514	100.0

**Input Sequence**	fig|469.3674.peg.2980 hypothetical protein [*Acinetobacter* IRMCBCU95U | 469.3674]						
	PROJECT ID	ACCESSION ID	ORGANISMS	CLASS	PROTEIN FUNCTION	PROTEIN ID	%IDENTITY
**Matched Family**	13001	XXX	Proteus mirabilis HI4320	Gammaproteobacteria	XXX		100.0

**Input Sequence**	fig|469.3674.peg.167 FIG00350337: hypothetical protein [*Acinetobacter* IRMCBCU95U | 469.3674]						
	PROJECT ID	ACCESSION ID	ORGANISMS	CLASS	PROTEIN FUNCTION	PROTEIN ID	%IDENTITY
**Matched Family**	17827	CP000863	*Acinetobacter baumannii* ACICU, complete genome.	Gammaproteobacteria	hypothetical protein	ACC57474	100.0

**Input Sequence**	fig|469.3674.peg.4767 hypothetical protein [*Acinetobacter* IRMCBCU95U | 469.3674]						
	PROJECT ID	ACCESSION ID	ORGANISMS	CLASS	PROTEIN FUNCTION	PROTEIN ID	%IDENTITY
**Matched Family**	21111	CP001182	*Acinetobacter baumannii* AB0057, complete genome.	Gammaproteobacteria	hypothetical protein	ACJ41836	100.0

**Input Sequence**	fig|469.3674.peg.3542 FIG00353857: hypothetical protein [*Acinetobacter* IRMCBCU95U | 469.3674]						
	PROJECT ID	ACCESSION ID	ORGANISMS	CLASS	PROTEIN FUNCTION	PROTEIN ID	%IDENTITY
**Matched Family**	17827	CP000863	*Acinetobacter baumannii* ACICU, complete genome.	Gammaproteobacteria	hypothetical protein	ACC57168	100.0

**Input Sequence**	fig|469.3674.peg.5182 hypothetical protein [*Acinetobacter* IRMCBCU95U | 469.3674]						
	PROJECT ID	ACCESSION ID	ORGANISMS	CLASS	PROTEIN FUNCTION	PROTEIN ID	%IDENTITY
**Matched**	17827	CP000863	*Acinetobacter*	Gammaproteobacteria	hypothetical protein	ACC58150	100.0

Family			*baumannii* ACICU, complete genome.				

Input Sequence	fig|469.3674.peg.1871 hypothetical protein [*Acinetobacter* IRMCBCU95U | 469.3674]						
	PROJECT ID	ACCESSION ID	ORGANISMS	CLASS	PROTEIN FUNCTION	PROTEIN ID	%IDENTITY
Matched Family	17827	CP000863	*Acinetobacter baumannii* ACICU, complete genome.	Gammaproteobacteria	hypothetical protein	ACC56874	100.0

Input Sequence	fig|469.3674.peg.3362 hypothetical protein [*Acinetobacter* IRMCBCU95U | 469.3674]						
	PROJECT ID	ACCESSION ID	ORGANISMS	CLASS	PROTEIN FUNCTION	PROTEIN ID	%IDENTITY
Matched Family	17827	CP000863	*Acinetobacter baumannii* ACICU, complete genome.	Gammaproteobacteria	hypothetical protein	ACC57075	100.0

Input Sequence	fig|469.3674.peg.4756 hypothetical protein [*Acinetobacter* IRMCBCU95U | 469.3674]						
	PROJECT ID	ACCESSION ID	ORGANISMS	CLASS	PROTEIN FUNCTION	PROTEIN ID	%IDENTITY
Matched Family	21111	CP001182	*Acinetobacter baumannii* AB0057, complete genome.	Gammaproteobacteria	hypothetical protein	ACJ41824	100.0

Input	fig|469.3674.peg.3440 FIG00351524: hypothetical protein [*Acinetobacter* IRMCBCU95U | 469.3674]

Sequence							
	PROJECT ID	ACCESSION ID	ORGANISMS	CLASS	PROTEIN FUNCTION	PROTEIN ID	%IDENTITY
**Matched Family**	17827	CP000863	*Acinetobacter baumannii* ACICU, complete genome.	Gammaproteobacteria	hypothetical protein	ACC57112	100.0

**Input Sequence**	fig|469.3674.peg.4758 hypothetical protein [*Acinetobacter* IRMCBCU95U | 469.3674]						
	PROJECT ID	ACCESSION ID	ORGANISMS	CLASS	PROTEIN FUNCTION	PROTEIN ID	%IDENTITY
**Matched Family**	21111	CP001182	*Acinetobacter baumannii* AB0057, complete genome.	Gammaproteobacteria	hypothetical protein	ACJ42071	100.0

**Input Sequence**	fig|469.3674.peg.5599 hypothetical protein [*Acinetobacter* IRMCBCU95U | 469.3674]						
	PROJECT ID	ACCESSION ID	ORGANISMS	CLASS	PROTEIN FUNCTION	PROTEIN ID	%IDENTITY
**Matched Family**	13001	XXX	Proteus mirabilis HI4320	Gammaproteobacteria	XXX		100.0

**Input Sequence**	fig|469.3674.peg.1334 hypothetical protein [*Acinetobacter* IRMCBCU95U | 469.3674]						
	PROJECT ID	ACCESSION ID	ORGANISMS	CLASS	PROTEIN FUNCTION	PROTEIN ID	%IDENTITY
**Matched Family**	17477	CP000521	*Acinetobacter baumannii* ATCC 17978, complete	Gammaproteobacteria	hypothetical protein	ABS89990	100.0

			genome.				

Input Sequence	fig|469.3674.peg.6805 FIG00354019: hypothetical protein [*Acinetobacter* IRMCBCU95U | 469.3674]						
	PROJECT ID	ACCESSION ID	ORGANISMS	CLASS	PROTEIN FUNCTION	PROTEIN ID	%IDENTITY
**Matched Family**	17827	CP000863	*Acinetobacter baumannii* ACICU, complete genome.	Gammaproteobacteria	hypothetical protein	ACC55765	100.0

Input Sequence	fig|469.3674.peg.4629 FIG00352575: hypothetical protein [*Acinetobacter* IRMCBCU95U | 469.3674]						
	PROJECT ID	ACCESSION ID	ORGANISMS	CLASS	PROTEIN FUNCTION	PROTEIN ID	%IDENTITY
**Matched Family**	13001	XXX	Proteus mirabilis HI4320	Gammaproteobacteria	XXX		100.0

Input Sequence	fig|469.3674.peg.7349 FIG00352875: hypothetical protein [*Acinetobacter* IRMCBCU95U | 469.3674]						
	PROJECT ID	ACCESSION ID	ORGANISMS	CLASS	PROTEIN FUNCTION	PROTEIN ID	%IDENTITY
**Matched Family**	17827	CP000863	*Acinetobacter baumannii* ACICU, complete genome.	Gammaproteobacteria	hypothetical protein	ACC56349	100.0

Input Sequence	fig|469.3674.peg.2874 FIG00350008: hypothetical protein [*Acinetobacter* IRMCBCU95U | 469.3674]						
	PROJECT	ACCESSION	ORGANISMS	CLASS	PROTEIN FUNCTION	PROTEIN	%IDENTITY

	ID	ID				ID	
**Matched Family**	17827	CP000863	*Acinetobacter baumannii* ACICU, complete genome.	Gammaproteobacteria	hypothetical protein	ACC56677	100.0

**Input Sequence**	fig|469.3674.peg.3627 hypothetical protein [*Acinetobacter* IRMCBCU95U | 469.3674]						
	PROJECT ID	ACCESSION ID	ORGANISMS	CLASS	PROTEIN FUNCTION	PROTEIN ID	%IDENTITY
**Matched Family**	17827	CP000863	*Acinetobacter baumannii* ACICU, complete genome.	Gammaproteobacteria	hypothetical protein	ACC57220	100.0

**Input Sequence**	fig|469.3674.peg.1297 FIG00351687: hypothetical protein [*Acinetobacter* IRMCBCU95U | 469.3674]						
	PROJECT ID	ACCESSION ID	ORGANISMS	CLASS	PROTEIN FUNCTION	PROTEIN ID	%IDENTITY
**Matched Family**	17477	CP000521	*Acinetobacter baumannii* ATCC 17978, complete genome.	Gammaproteobacteria	hypothetical protein	ABS89988	100.0

**Input Sequence**	fig|469.3674.peg.7211 hypothetical protein [*Acinetobacter* IRMCBCU95U | 469.3674]						
	PROJECT ID	ACCESSION ID	ORGANISMS	CLASS	PROTEIN FUNCTION	PROTEIN ID	%IDENTITY
**Matched Family**	30993	CP001172	*Acinetobacter baumannii* AB307-0294, complete genome.	Gammaproteobacteria	hypothetical protein	ACJ56002	100.0

Input Sequence	fig|469.3674.peg.4155 hypothetical protein [*Acinetobacter* IRMCBCU95U | 469.3674]						
	PROJECT ID	ACCESSION ID	ORGANISMS	CLASS	PROTEIN FUNCTION	PROTEIN ID	%IDENTITY
Matched Family	17827	CP000863	*Acinetobacter baumannii* ACICU, complete genome.	Gammaproteobacteria	hypothetical protein	ACC57582	100.0

Input Sequence	fig|469.3674.peg.4846 hypothetical protein [*Acinetobacter* IRMCBCU95U | 469.3674]						
	PROJECT ID	ACCESSION ID	ORGANISMS	CLASS	PROTEIN FUNCTION	PROTEIN ID	%IDENTITY
Matched Family	17827	CP000863	*Acinetobacter baumannii* ACICU, complete genome.	Gammaproteobacteria	hypothetical protein	ACC57898	100.0

Input Sequence	fig|469.3674.peg.5036 FIG00351435: hypothetical protein [*Acinetobacter* IRMCBCU95U | 469.3674]						
	PROJECT ID	ACCESSION ID	ORGANISMS	CLASS	PROTEIN FUNCTION	PROTEIN ID	%IDENTITY
Matched Family	17827	CP000863	*Acinetobacter baumannii* ACICU, complete genome.	Gammaproteobacteria	hypothetical protein	ACC57996	100.0

Input Sequence	fig|469.3674.peg.2579 hypothetical protein [*Acinetobacter* IRMCBCU95U | 469.3674]						
	PROJECT	ACCESSION	ORGANISMS	CLASS	PROTEIN FUNCTION	PROTEIN	%IDENTITY

	ID	ID				ID	
**Matched Family**	17827	CP000863	*Acinetobacter baumannii* ACICU, complete genome.	Gammaproteobacteria	hypothetical protein	ACC56525	100.0

Supplementary Table S8: Fragment of plasmid sequence identified in IRMCBCU95U.

Name	Plasmid_sequence_identified_in_IRMCBCU95U
IncFIB(pNDM-Mar)	TATCAAGAGCCTTAAGGCGAAGATAAACCTTATAGTCAATCTGATAGA<br>GCTCCTGAATCCGGGGATCTCCCTCTATCTCAATCTCATTGGTTTTTATG<br>TTGTACCGTGCCCAGTTAATGAGGCCGGTCGTGAGGGTTGAATCTTCAT<br>TGCGGAACTGAAAAGTGACCATTTTCAGTTTAATCATCGAAGCATCGA<br>TACGTTGTTTCATGGGCTTATTCGACCTCGAGGGGTCAAACCCACAATT<br>TTTAAGGAAGGTGCTAAAAGGAAGCTTAATCCTTCCTGAGTAATGGCC<br>ATGGTCATGTAATGAACGGACTATACCAAGCCATACACGGAAATCTGT<br>CCCCATATCAAGACGAGGGCTGGATAGCGATATTTCTTTGTAGCCTTC<br>ACTGGATGCGATCTGCATATGCTGTAAATCGCGGGACACATCCATCGT<br>AAC

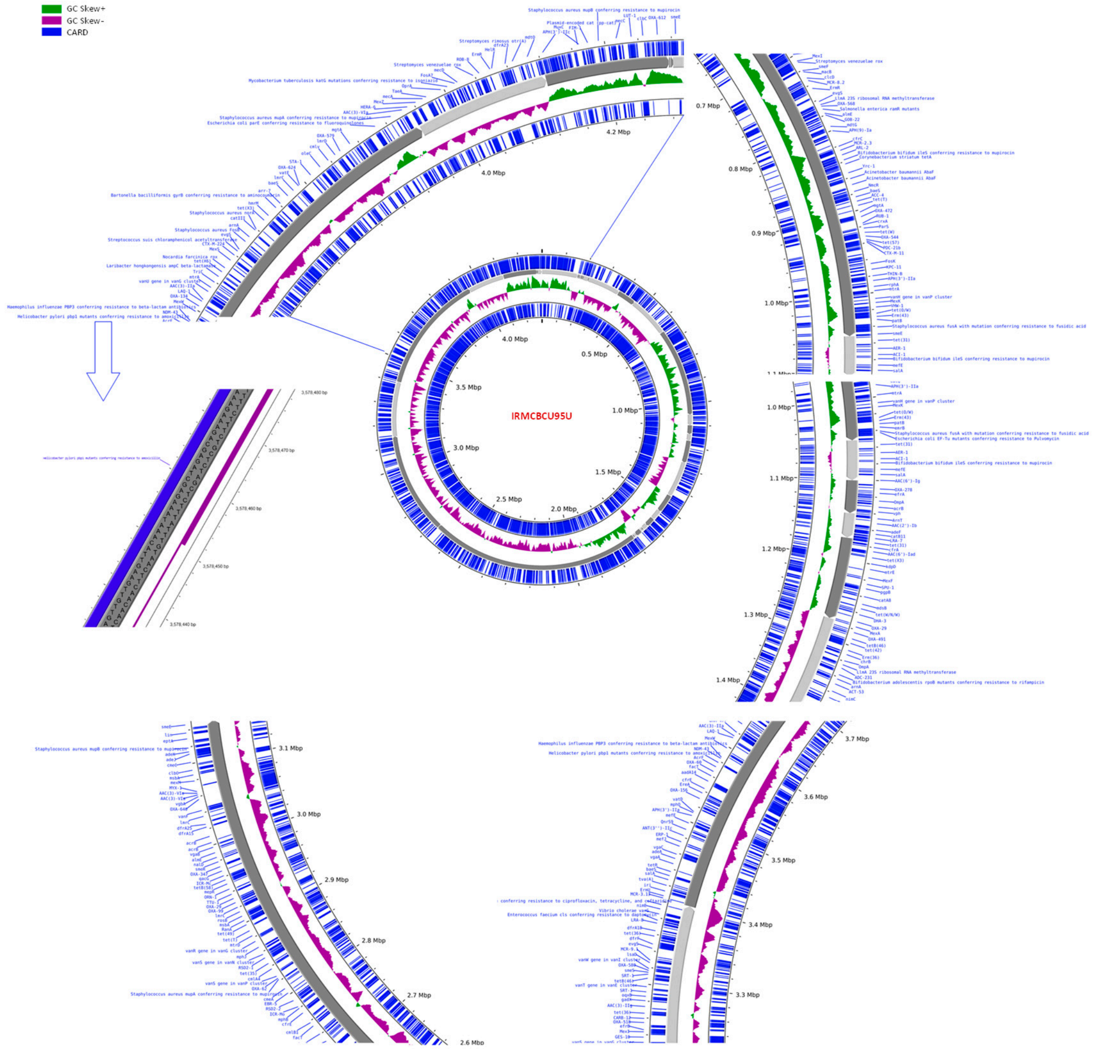

Supplementary Figure S1. Genome map of *Acinetobacter* IRMCBCU95U genome, with selected genomic regions projected to indicate the location of mutated genes.

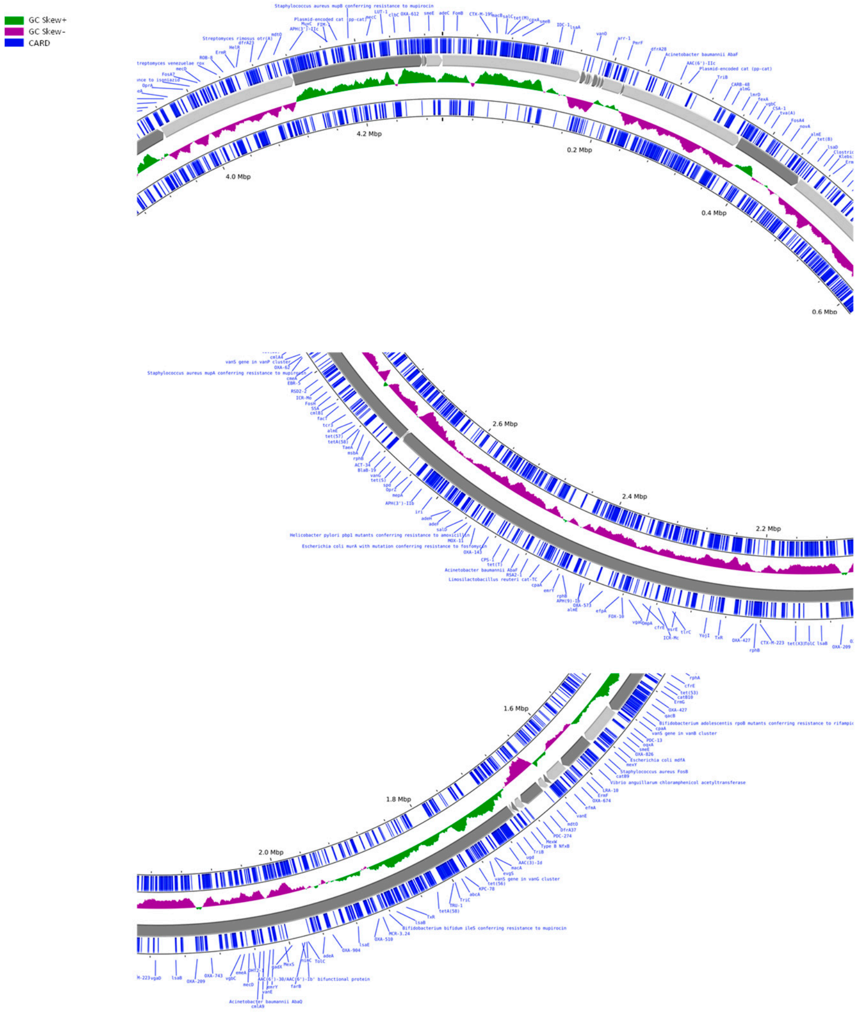

Supplementary Figure S2. *Acinetobacter* IRMCBCU95U Genome map with selected genomic regions indicating the location of mutated genes associated with Mupirocin (Top panel), amoxicillin (Middle panel) and rifampicin (Bottom panel).
